# Microcephalin 1/BRIT1-TRF2 interaction promotes telomere replication and repair, linking telomere dysfunction to primary microcephaly

**DOI:** 10.1038/s41467-020-19674-0

**Published:** 2020-11-17

**Authors:** Alessandro Cicconi, Rekha Rai, Xuexue Xiong, Cayla Broton, Amer Al-Hiyasat, Chunyi Hu, Siying Dong, Wenqi Sun, Jennifer Garbarino, Ranjit S. Bindra, Carl Schildkraut, Yong Chen, Sandy Chang

**Affiliations:** 1 Department of Laboratory Medicine, Yale University School of Medicine, 330 Cedar St., New Haven, CT 06520 USA; 2 State Key Laboratory of Molecular Biology, Shanghai Institute of Biochemistry and Cell Biology, Center for Excellence in Molecular Cell Science, Chinese Academy of Sciences, Shanghai, 200031 China; 3 Tri- Institutional MD/PhD Program, Weill Cornell Medical College, New York, NY 10065 USA; 4 Department of Molecular Biophysics and Biochemistry, Yale University School of Medicine, 330 Cedar St., New Haven, CT 06520 USA; 5 Department of Therapeutic Radiology, Yale University School of Medicine, 330 Cedar St., New Haven, CT 06520 USA; 6 Department of Experimental Pathology, Yale University School of Medicine, 330 Cedar St., New Haven, CT 06520 USA; 7 Department of Cell Biology, Albert Einstein College of Medicine, 1300 Morris Park Avenue, Bronx, NY 10461 USA; 8 Department of Pathology, Yale University School of Medicine, 330 Cedar St., New Haven, CT 06520 USA

**Keywords:** Molecular biology, Telomeres, Neuroscience

## Abstract

Telomeres protect chromosome ends from inappropriately activating the DNA damage and repair responses. Primary microcephaly is a key clinical feature of several human telomere disorder syndromes, but how microcephaly is linked to dysfunctional telomeres is not known. Here, we show that the microcephalin 1/BRCT-repeats inhibitor of hTERT (MCPH1/BRIT1) protein, mutated in primary microcephaly, specifically interacts with the TRFH domain of the telomere binding protein TRF2. The crystal structure of the MCPH1–TRF2 complex reveals that this interaction is mediated by the MCPH1 _330_YRLSP_334_ motif. TRF2-dependent recruitment of MCPH1 promotes localization of DNA damage factors and homology directed repair of dysfunctional telomeres lacking POT1-TPP1. Additionally, MCPH1 is involved in the replication stress response, promoting telomere replication fork progression and restart of stalled telomere replication forks. Our work uncovers a previously unrecognized role for MCPH1 in promoting telomere replication, providing evidence that telomere replication defects may contribute to the onset of microcephaly.

## Abstract (web summary)

Primary microcephaly is a clinical feature of several human telomere disorder syndromes. Here the authors reveal a role of Microcephalin 1 in promoting telomere replication and repair.

## Introduction

Primary microcephaly (MCPH) is a recessive neurodevelopmental disorder characterized by reduced cerebral cortex volume and mental retardation, affecting up to 1 in 30,000 newborns worldwide^1^. To date, several genes have been linked to MCPH, many of which are expressed in the ventricular zone of the developing brain during neurogenesis, consistent with their roles in neural progenitor cell (NPC) proliferation^2^. Some of these genes are involved in DNA damage response (DDR) pathways, implicating DDR and cell cycle checkpoints deficiencies in brain developmental defects. For example, MCPH is a clinical feature of both Seckel syndrome, which is caused by mutations in the ataxia-telangiectasia and Rad3-related (ATR) gene, and Nijmegen breakage syndrome, linked to mutations in the MRE11-RAD50-NBS1 (MRN) complex component NBS1^3,4^. Moreover, downstream neighbor of SON (DONSON) gene mutations have been identified as another cause of neurogenesis defects^5,6^. DONSON is a component of the DNA replication complex and is involved in stabilization of replication forks and cell cycle checkpoint activation^5^. Cells from patients with DONSON mutations display high levels of replication stress and DNA damage. These results suggest that abrogation of DNA damage and DNA replication functions impair NPCs proliferation during development^5^.

Microcephalin 1/BRCT-repeats inhibitor of hTERT expression (MCPH1/BRIT1) is the first gene identified in MCPH^7–10^, and several loss-of-function MCPH1 mutations have been found in MCPH patients^11–14^. MCPH1 plays important roles in DDR upon induction of double-strand breaks (DSBs)^15–17^. It contains two BRCA1 C-terminus (BRCT) domains at its C-terminus that mediate its interaction with the DNA damage marker γ-H2AX. This interaction facilitates the localization of MCPH1 to DSBs, where it promotes the recruitment of several DDR proteins involved in both ataxia-telangiectasia mutated (ATM)- and ATR-dependent DNA damage signaling^16,18^. MCPH1 patient cells display elevated nuclear fragmentation in response to replication stress-induced DNA damage, suggesting defects in DNA repair in the absence of MCPH1^19^. Indeed, MCPH1 recruits the BRCA1–BRCA2–RAD51 complex to DNA damage sites for homology-directed repair (HDR), an error-free pathway that repairs nucleolytically processed DNA ends using homologous sister chromatids as templates^15,17,20^. More recent findings suggest that MCPH1 also participates in classical non-homologous end joining (C-NHEJ)-mediated repair, which ligates two DNA ends with little to no processing^17,21^. The role of MCPH1 in DNA repair, as well as its importance in regulating centrosome integrity and both the intra-S and the G_2_/M cell-cycle checkpoints^15,22^, reflect its importance in maintaining genome stability. In support of this observation, MCPH1 patient cells exhibit premature chromosome condensation (PCC) defects in DNA damage-induced G_2_/M checkpoint arrest as well as prolonged persistence of ionizing radiation-induced γ-H2AX foci^19,23–25^. Consequently, MCPH1 deficiency results in chromosomal aberrations and genomic instability, contributing to malignant transformation and tumorigenesis^16,20,26^. In recent years, MCPH1 mutations have been observed in breast, ovarian, prostate, and lung cancers, highlighting a link between MCPH1 dysfunction and tumorigenesis^16,22,27^. In addition, MCPH1 knockout mice develop increased genome instability and cancer susceptibility^26^, suggesting that MCPH1 functions as a tumor suppressor to maintain genome integrity.

Genome integrity is also maintained by telomeres, G-rich repetitive DNA–protein complexes that cap all eukaryotic chromosome ends^28,29^. Inappropriate activation of DDR and DNA repair pathways at telomeres are inhibited by a six-protein complex termed shelterin. The shelterin components TRF1 and the TRF2-RAP1 heterodimer specifically recognize double stranded telomeric repeats, while POT1 binds the single stranded (ss) 3’ overhang. TPP1 interacts with POT1 and promotes its recruitment to telomeres, while TIN2 bridges POT1-TPP1 to TRF1 and TRF2-RAP1^30,31^. Removal of shelterin components results in telomere uncapping, generating “dysfunctional” telomeres that trigger a DDR mediated by the kinases ATM and ATR^32,33^. DNA damage repair activity at dysfunctional telomeres can lead to chromosome end-to-end fusions and increased genomic instability^34–36^. Individual shelterin proteins evolved to protect telomeres from engaging in distinct DNA repair pathways. TRF2 protects telomeres from activating ATM-Checkpoint kinase 2 (CHK2)-dependent C-NHEJ^33,37^, while TRF2-RAP1 and POT1-TPP1 prevent ATR-Checkpoint kinase 1 (CHK1)-mediated HDR at telomeres^32,38,39^. Finally, TRF2-RAP1 and POT1-TPP1 cooperate to inhibit ATR-CHK1-dependent Alternative NHEJ (A-NHEJ), a microhomology-mediated error-prone repair pathway commonly found in human cancers^36,40,41^.

A previous report showed that MCPH1 interacts with the TRF Homology (TRFH) domain of TRF2 and localizes at dysfunctional telomeres^42^. However, how MCPH1 functions at dysfunctional telomeres is unclear. In this report, we show that TRF2-mediated recruitment of MCPH1 to dysfunctional telomeres is required for HDR. Additionally, we highlight a previously unrecognized role for MCPH1 in promoting telomere replication in a TRF2-dependent manner. Our work unravels an essential role for MCPH1 in telomere replication and repair, connecting primary microcephaly with telomere dysfunction.

## Results

### Human MCPH1 interacts with the TRF2 TRFH domain through a canonical telomere binding motif

**Fig. 1:**
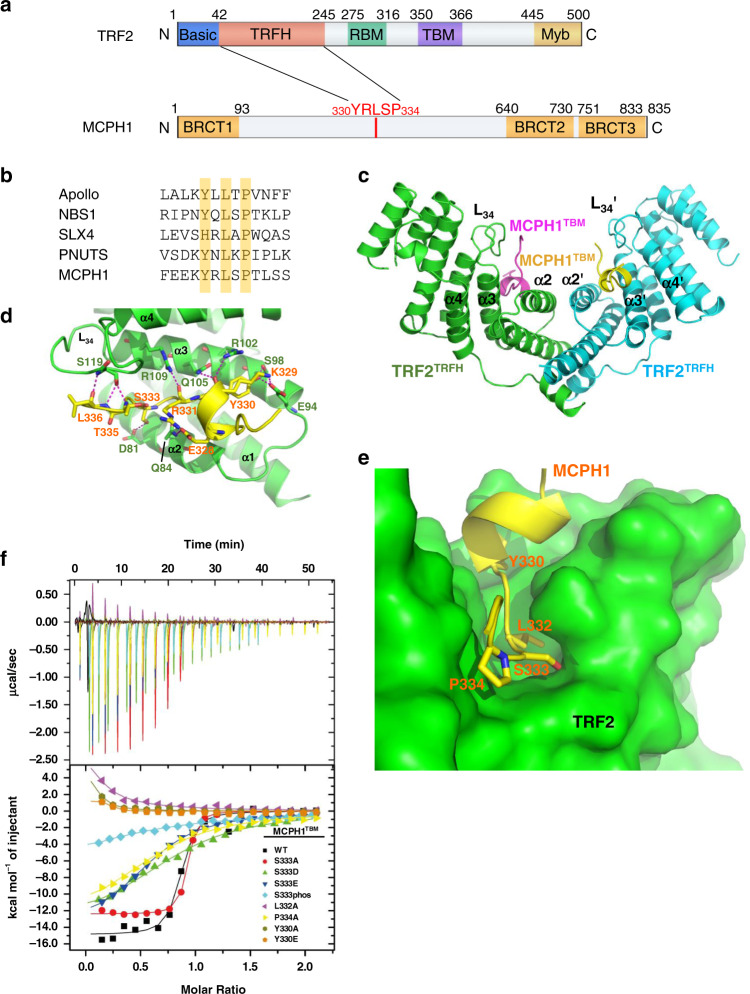
Structure of the human TRF2^TRFH^–MCPH1^TBM^ complex. **a** Schematic representation of human TRF2 and MCPH1 domains, showing the interaction domains. **b** Comparison of MCPH1^TBM^ sequence with those of known TRF2-interacting protein. The conserved amino acid Y/H-X-L-X-P consensus sequence is highlighted. **c** Dimeric TRF2–MCPH1 structure is shown in a ribbon representation (TRF2, green/cyan; MCPH1, magenta/yellow). **d** TRF2 and MCPH1 are depicted in green and yellow, respectively, and the residues involved in their interaction are shown. Hydrogen bonding: magenta dashed lines. **e** MCPH1^TBM^ (in yellow) is buried inside a hydrophobic pocket formed by TRF2 helices α2 and α3 (in green). **f** ITC measurement of the interactions between TRF2^TRFH^ and different MCPH1^TBM^ mutant peptides. S333phos is a phosphorylated S333 peptide synthesized using a phosphorylated serine as starting material. Equilibrium dissociation constant (*K*_D_) values derived from ITC data are shown in Table 2.

Analysis of the human MCPH1 amino acid sequence revealed the presence of a _330_**Y**R**L**S**P**_334_ sequence typical of the TRFH-binding motif (TBM) Y/F/H-X-L-X-P (where X is any amino acid) found in proteins that interact with TRF2, including Apollo/hSNM1B, PNUTS, SLX4, and NBS1^42–45^ (Fig. 1a, b). To understand the structural basis of this interaction, we solved the crystal structure of TRF2^TRFH^–MCPH1^TBM^ complex at a resolution of 2.15 Å by molecular replacement (Fig. 1c–e, and Table 1). MCPH1^TBM^ is well ordered as evidenced by good electron density in the crystals in the final atomic model (Supplementary Fig. 1a). As predicted, the binding mode of MCPH1^TBM^ to TRF2^TRFH^ closely resembles the interaction between Apollo^TBM^ and TRF2^TRFH^ ^43^. MCPH1^TBM^ binds to TRF2^TRFH^ in an extended conformation with a short helix at the N-terminus across the concave face of each TRF2 subunit, and involves an extensive set of interactions, including both Van der Waals contacts and electrostatic polar interactions (Fig. 1c, d). The most critical interaction interface is the central portion of MCPH1^TBM^ (_330_YRLSP_334_). MCPH1^Y330^ fits into a hydrophobic cleft formed by the aliphatic side chain of TRF2^L101^ and TRF2^R102^, and the hydroxyl group of MCPH1^Y330^ forms two hydrogen bonds with TRF2^S98^ and TRF2^E94^ (Supplementary Fig. 1b). MCPH1^L332^ is completely buried in a deep pocket surrounded by hydrophobic residues from α2 and α3 of TRF2, and MCPH1^P334^ stacks with the aromatic ring of TRF2^F120^ (Fig. 1e and Supplementary Fig. 1c). Besides these three invariant residues, the flanking residues also contribute to TRFH-binding through hydrogen-bonding interactions. The backbone carbonyl of MCPH1^R331^ forms a hydrogen bond with TRF2^R109^ (Fig. 1d). The hydroxyl group of MCPH1^S333^ forms a hydrogen bond with TRF2^D81^, and thus phosphorylation of this serine residue is predicted to disrupt the interaction between TRF2 and MCPH1 (Fig. 1e and Supplementary Fig. 1c).

**Table 1:** Data collection and refinement statistics.

	MCPH1^TBM^-TRF2^TRFH^
*Data collection*	
Space group	*P2*_*1*_*2*_*1*_*2*_*1*_
Cell dimensions	
a, b, c (Å)	60.737, 74.941, 98.418
α, β, γ (°)	90, 90, 90
Resolution (Å)	2.15
R_sym_ (Å) (high res. Shell)	0.077 (0.599)
I/σI (high res. Shell)	25.2 (3.0)
Completeness (%) (high res. Shell)	99.9 (99.6)
Redundancy (high res. Shell)	13.0 (11.8)
*Refinement*	
Resolution (Å)	31.89–2.15
No. reflections	24,962
R_work_/R_free_ (%)	18.9/23.5
No. atoms	
TRF2	3196
MCPH1	239
Solvent	160
B-factors (Å^2^)	
TRF2	39.92
MCPH1	47.30
Solvent	43.52
R.m.s. deviations	
Bond lengths (Å)	0.005
Bond angles (°)	0.645
Ramachandran plot	
Preferred regions (%)	97.79
Allowed regions (%)	2.21
Outliers regions (%)	0

Isothermal titration calorimetry (ITC) confirmed the MCPH1^TBM^–TRF2^TRFH^ interaction with an equilibrium dissociation constant (*K*_D_) of 0.24 μM (Fig. 1f and Table 2). Mutating any one of the three conserved residues in the TRFH-interacting motif (Y330, L332, or P334) either completely abolished this interaction or greatly reduced it (Fig. 1f and Table 2), confirming the importance of these hydrophobic contacts in  mediating the MCPH1^TBM^–TRF2^TRFH^ interaction.

**Table 2:** Equilibrium dissociation constant (*K*_D_) values.

MCPH1^TBM^	*K*_D_ (μM)
WT	0.24 ± 0.10
Y330A	N.D.
Y330E	N.D.
L332A	N.D.
S333A	0.16 ± 0.02
S333D	21 ± 4
S333E	14 ± 1
S333PHOS	79 ± 23
P334A	24 ± 4

*K*_D_ values, calculated from ITC data, for binding of MCPH1^TBM^ and several MCPH1^TBM^ mutant peptides with TRF2^TRFH^. ND: not detected.

The MCPH1 _330_YRLSP_334_ TBM motif resembles the NBS1 _429_YQLSP_433_ TMB motif^45^. We previously reported that the phosphorylation status of NBS1 serine 432 modulates NBS1’s telomeric localization and DDR in a cell cycle-dependent manner^45^. Structurally, MCPH1 S333 fits snugly into a hydrophobic cleft of TRF2^TRFH^ (Fig. 1e) and phosphorylation of S333 should disrupt the TRF2–MCPH1 interaction. Therefore, we asked whether MCPH1’s interaction with TRF2^TRFH^ domain might be modulated by serine 333 phosphorylation. As expected, the synthesized phosphorylated MCPH1^TBM^ peptide (S333phos) interacted poorly with TRF2^TRFH^ (*K*_D_: 79 μM). We also generated MCPH1^TBM^ peptides where S333 was mutated to either alanine (A) or phosphomimetic aspartic acid (D) or glutamic acid (E) and measured their binding to TRF2^TRFH^ using ITC. While MCPH1^S333A^ had a greater affinity than wild-type (WT) MCPH1 for TRF2^TRFH^ (*K*_D_: 0.16 μM), MCPH1^S333D^ and MCPH1^S333E^ displayed more than 50-fold weaker interactions with TRF2^TRFH^ (*K*_D_: 21 μM and 14 μM, respectively), suggesting that the phosphorylation status of MCPH1^S333^ could modulate MCPH1^TBM^ binding to the TRF2^TRFH^ domain (Table 2).

### Human MCPH1’s telomeric localization depends on its interaction with TRF2

**Fig. 2:**
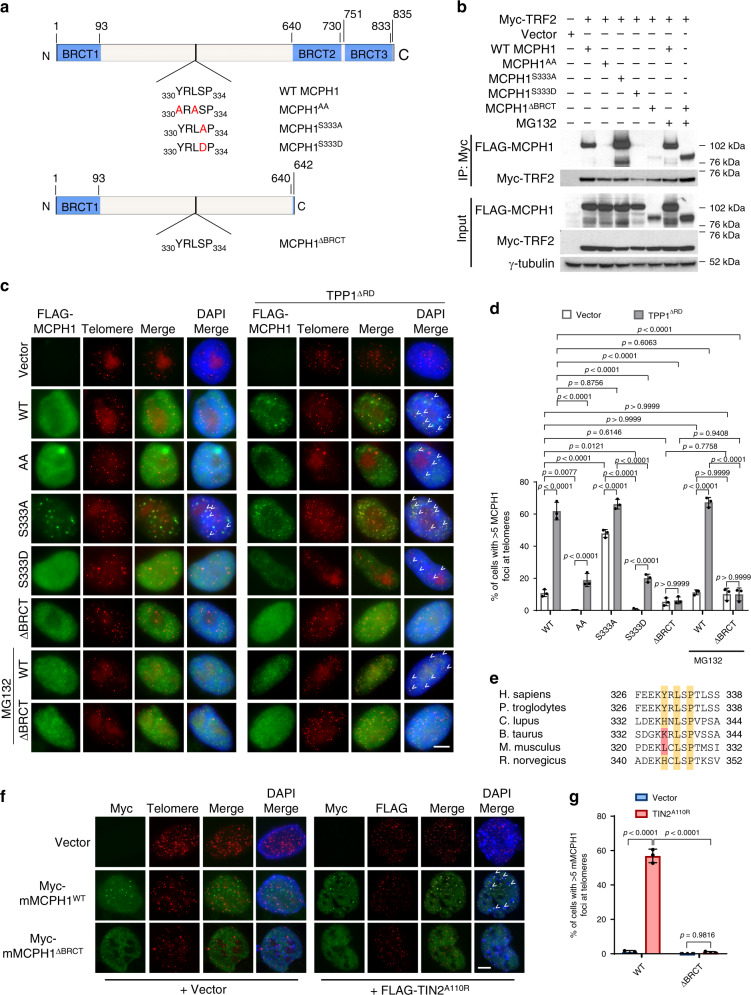
MCPH1^S333^ phosphorylation modulates MCPH1–TRF2 interaction. **a** Schematic representation of the human MCPH1 constructs generated. The TBM sequence for each construct is shown and amino acid substitutions are depicted in red. **b** Co-IP with anti-Myc antibody-conjugated agarose beads from lysates of 293T cells expressing Myc-tagged TRF2 and either FLAG-tagged WT MCPH1, FLAG-MCPH1^TBM^ mutants or FLAG-MCPH1^ΔBRCT^. γ-tubulin was used as a loading control. The blot shown is representative of four independent experiments. **c** Immunostaining-PNA FISH in HeLa cells overexpressing either empty vector or one of the FLAG-tagged WT MCPH1, MCPH1^AA^, MCPH1^S333A^, MCPH1^S333D^, MCPH1^ΔBRCT^ constructs and either empty vector or HA-TPP1^ΔRD^. MCPH1 proteins were detected using an anti-FLAG antibody (green) while telomeres were detected with a Cy3-OO-(CCCTAA)_4_ PNA probe (red). 4,6-diamidino-2-phenylindole (DAPI) was used to stain nuclei (blue). Representative images from three independent experiments are shown. White arrowheads indicate MCPH1 foci co-localizing with the telomere signals. Scale bar: 5 μm. **d** Quantification of the percentage of cells with >5 MCPH1-positive foci at telomeres from **c**. Data represent the mean ± standard deviation (SD) from three independent experiments. At least 200 cells were scored for each sample. Significance was determined with one-way analysis of variance (ANOVA) followed by Tukey’s multiple comparison test. *P* values are shown. **e** Comparison of MCPH1^TBM^ amino acidic sequence across several mammalian species. The conserved residues are highlighted in yellow, while the residues in red differ from the canonical Y/H-X-L-X-P amino acid sequence. **f** Immunostaining-PNA FISH in MEFs overexpressing either Myc-WT MCPH1 or Myc-MCPH1^ΔBRCT^ together with either empty vector or FLAG-TIN2^A110R^. Myc-MCPH1 proteins were detected with a Myc antibody (green), while telomeres were detected with either a telomeric PNA probe or a FLAG antibody that recognizes FLAG-TIN2^A110R^ (in red). Nuclei were stained with DAPI (blue). Representative images from three independent experiments. Scale bar: 5 μm. **g** Quantification of the percentage of cells with >5 MCPH1-positive foci at telomeres from **f**. Data are representative of the mean of three independent experiments ± SD. A minimum of 200 cells for each sample were scored. Statistical analysis: one-way ANOVA followed by Tukey’s multiple comparison test.

To confirm our ITC data, we overexpressed Myc-TRF2 and one of the following FLAG-tagged MCPH1 constructs in 293T cells: WT MCPH1, MCPH1^Y330A, L332A^ (MCPH1^AA^), MCPH1^S333A^, MCPH1^S333D^ and MCPH1^ΔBRCT^ (Fig. 2a). MCPH1^ΔBRCT^ is a human breast cancer mutation lacking the C-terminal BRCT domains and is defective in DNA damage response^16^. Co-immunoprecipitation (Co-IP) experiments revealed a robust interaction between WT MCPH1 and TRF2, while the binding of MCPH1^AA^ to TRF2 was completely abolished, confirming that TRF2–MCPH1 interaction is dependent on the MCPH1^TBM^ (Fig. 2b). In agreement with our ITC data, mutating MCPH1^S333^ to alanine increased its interaction with TRF2, while mutating it to the phosphomimetic aspartic acid (MCPH1^S333D^) disrupted this interaction. Mutating the essential TRF2^TRFH^ residue F120 to alanine^43,45^ also prevented MCPH–TRF2 interaction (Supplementary Fig. 2a). We also did not detect any interaction between MCPH1 and TRF1, confirming that MCPH1^TBM^ specifically interacts with TRF2^TRFH^ (Supplementary Fig. 2b). Stabilizing MCPH1^ΔBRCT^ with the proteasome inhibitor MG132 resulted in its robust interaction with TRF2 (Fig. 2b), revealing that the MCPH1–TRF2 interaction is not dependent upon MCPH1’s C-terminal BRCT domains.

Next, we analyzed the localization of FLAG-tagged WT and mutant MCPH1 in HeLa cells by immunostaining and telomere PNA-FISH, using a FLAG-specific antibody and a (CCCTAA)_4_-PNA probe to visualize telomeres (Fig. 2c). WT MCPH1 formed telomeric foci in only ~11% of the cells examined, while no telomeric localization was found in cells expressing the MCPH1^AA^ mutant (Fig. 2d), suggesting that a limited amount of WT MCPH1 is recruited by TRF2 to functional telomeres. MCPH1^S333A^ localized to telomeres in ~48% of the cells examined, while telomeric localization of MCPH1^S333D^ was almost undetectable, supporting the hypothesis that the phosphorylation status of MCPH1^S333^ determines its interaction with TRF2. In the presence of MG132, MCPH1^ΔBRCT^ localized to telomeres with an efficiency similar to WT MCPH1. In agreement with these results, we found significantly reduced telomeric localization of both endogenous and FLAG-tagged MCPH1 after TRF2 depletion, confirming that MCPH1 recruitment to telomeres is TRF2-mediated (Supplementary Fig. 2c–h). We next analyzed MCPH1 localization at dysfunctional telomeres by expressing the dominant negative TPP1^ΔRD^ mutant that lacks the POT1 recruiting domain, resulting in the formation of unprotected single-stranded telomeric overhangs^32^. Telomeric localization of both WT MCPH1 and MCPH1^S333A^ increased significantly (from ~11% to ~62% and from ~48% to ~66%, respectively) in HeLa cells overexpressing TPP1^ΔRD^ (Fig. 2c, d). Interestingly, in ~20% of cells examined, both MCPH1^AA^ and MCPH1^S333D^ were detected on dysfunctional telomeres, suggesting that MCPH1 can also interact with dysfunctional telomeres independent of TRF2. We found that these mutants recognize dysfunctional telomeres as damaged DNA through interaction with γ-H2AX^18^, since the MCPH1^ΔBRCT^ mutant that lacks the BRCT domains necessary for γ-H2AX binding^46^ does not show any preference for localization to dysfunctional telomeres over functional telomeres (Fig. 2c, d). Our data suggest that MCPH1 localizes to functional telomeres only through its interaction with TRF2, while it localizes to dysfunctional telomeres lacking POT1-TPP1 by interacting with both TRF2 and γ-H2AX. In contrast, WT MCPH1, MCPH1^S333A^ and MCPH1^S333D^ all localized to genomic DSBs at similar levels, suggesting that localization of MCPH1 to genomic DSBs is dependent only on its interaction with γ-H2AX (Supplementary Fig. 2i, j).

Interestingly, the MCPH1^TBM^ is only partially conserved in murine *Mcph1*, with a leucine residue substituting for a tyrosine in position 324 (Fig. 2e). Co-IP experiments showed no detectable interaction between Myc-mMCPH1 and FLAG-mTRF2 (Supplementary Fig. 3a). However, Myc-mMCPH1 localized to telomeres in WT mouse embryo fibroblasts (MEFs) only upon telomere dysfunction induced by either TPP1^ΔRD^ or TIN2^A110R^ expression^31,32,36^ (Fig. 2f, g, Supplementary Fig. 3b–d) presumably through interaction with γ-H2AX. Supporting of this notion, we did not detect MCPH1 foci at telomeres in *H2AX*^*-/-*^ MEFs or when we overexpressed in WT MEFs a truncated mMCPH1 mutant lacking the C-terminal BRCT domains (Myc-mMCPH1^ΔBRCT^) (Fig. 2f, g, Supplementary Fig. 3e, f). Taken together, these results suggest that mMCPH1 does not directly interact with TRF2 to localize to telomeres but can localize to dysfunctional telomeres through interaction with γ-H2AX.

### MCPH1 promotes the recruitment of DDR factors to telomeres lacking POT1-TPP1

**Fig. 3:**
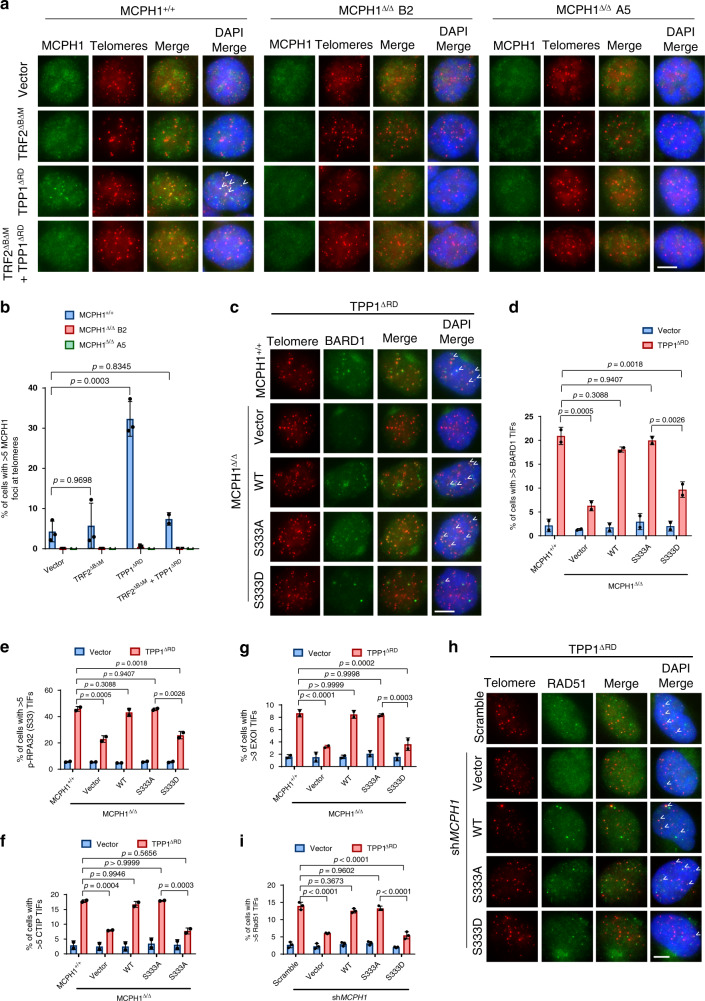
MCPH1 promotes the recruitment of DDR factors at dysfunctional telomeres lacking POT1-TPP1 in a TRF2-dependent manner. **a** Immunostaining for MCPH1 telomeric localization in MCPH1^+/+^ HCT116 and two CRISPR/Cas9 MCPH1^Δ/Δ^ HCT116 clones (B2 and A5) overexpressing the indicated constructs. MCPH1 localization at telomeres was assessed using an anti-MCPH1 antibody (green) and telomere were detected through PNA-FISH (red). Representative images from either three (MCPH1^+/+^ and MCPH1^Δ/Δ^ B2 + vector, TRF2^ΔBΔM^ or TPP1^ΔRD^) or two (MCPH1^Δ/Δ^ A5 and samples with TRF2^ΔBΔM^ + TPP1^ΔRD^) independent experiments. **b** Percentage of cells with >5 MCPH1-positive foci at telomeres from (**a**). Data represent mean values ± SD. *n* = 3 for MCPH1^+/+^ and MCPH1^Δ/Δ^ B2 + vector, TRF2^ΔBΔM^ and TPP1^ΔRD^; *n* = 2 for MCPH1^Δ/Δ^ A5 and samples with TRF2^ΔBΔM^ + TPP1^ΔRD^. A minimum of 200 cells were scored for each sample. **c** BARD1 TIF analysis in WT and MCPH1^Δ/Δ^ cells reconstituted with either empty vector, WT MCPH1, MCPH1^S333A^ or MCPH1^S333D^ and overexpressing either empty vector or FLAG-TPP1^ΔRD^. Representative images from two independent experiments. **d** Percentage of cells with >5 BARD1-positive TIFs from (**c**). The means from two independent experiments ± SD are shown. At least 200 cells were scored for each sample. **e**–**g** Quantification of the percentage of cells with >5 p-RPA32 (S33) (**e**) and CTIP (**f**) TIFs and with >3 EXOI (**g**) TIFs in WT and MCPH1^Δ/Δ^ cells reconstituted with the indicated constructs and overexpressing either empty vector or FLAG-TPP1^ΔRD^ (see also Supplementary Fig. 6f–h). Data represent the mean ± SD from two independent experiments. At least 200 cells were scored for each sample. **h** RAD51 TIF analysis in TPP1^ΔRD^-expressing U2OS cells treated with either scrambled or *MCPH1* shRNA and reconstituted with either empty vector, WT MCPH1, MCPH1^S333A^ or MCPH1^S333D^. Representative images from three independent experiments. **i** Quantification of the percentage of cells with >5 RAD51-positive TIFs shown in (**h**). Data represent the mean values ± SD, *n* = 3. At least 200 cells were scored for each sample. The statistical analysis for **b**, **d**–**g** and **i** was performed using one-way ANOVA followed by Tukey’s multiple comparison test. Scale bars for **a**, **c**, **h**: 5 μm.

Given its interaction with TRF2 and localization to telomeres, we asked what roles MCPH1 plays when recruited to telomeres. First, we analyzed MCPH1’s role in both ATM- and ATR-dependent DNA damage signaling at telomeres. Using CRISPR/Cas9 editing, we generated two MCPH1^Δ/Δ^ HCT116 cell lines (clone B2 and clone A5). We successfully verified the loss of MCPH1 protein in these two clones by Western blot analysis (Supplementary Fig. 4a) and the absence of MCPH1-positive foci by immunofluorescence (Fig. 3a, b). MCPH1-deleted cells displayed prophase-like nuclei suggestive of the PCC phenotype previously observed in primary microcephaly patients bearing MCPH1 mutations^25,47^ (Supplementary Fig. 4b, c). Moreover, MCPH1^Δ/Δ^ cells displayed increased telomere length compared to MCPH1^+/+^ cells (Supplementary Fig. 4d), consistent with MCPH1’s role in inhibiting telomerase^9,10^. We induced telomere dysfunction in these cells by expressing TRF2^ΔBΔM^, a dominant negative mutant that removes endogenous TRF2 from telomeres, inducing ATM-dependent DNA damage signaling^48^. We also expressed TPP1^ΔRD^ to activate an ATR-dependent DDR^32^ or both mutants together (TRF2^ΔBΔM^/TPP1^ΔRD^) to activate both ATM/ATR-dependent signaling^40^ (Supplementary Fig. 4e). Analysis of γ-H2AX Telomere Dysfunction-Induced Foci (TIFs)^49^ confirmed the induction of telomere dysfunction in MCPH1^Δ/Δ^ clones (Supplementary Fig. 4f and 4i). γ-H2AX levels were not affected by MCPH1 status, since γ-H2AX localization to DNA damage sites occurs independently of MCPH1^16^. We next used immunostaining to analyze endogenous MCPH1 localization in MCPH1^+/+^ cells (Fig. 3a). Telomeric localization of endogenous MCPH1 was significantly increased in TPP1^ΔRD^-expressing MCPH1^+/+^ cells, but not in cells expressing TRF2^ΔBΔM^ or both TRF2^ΔBΔM^ and TPP1^ΔRD^, suggesting that MCPH1 preferentially localized to telomeric ends lacking POT1-TPP1 (Fig. 3b). In agreement with this hypothesis, analysis in MCPH1^Δ/Δ^ cell lines revealed a significantly reduced amount of 53BP1 and BARD1 TIFs after TPP1^ΔRD^ expression compared to MCPH1^+/+^ cells, but not after expression of either TRF2^ΔBΔM^ or both TRF2^ΔBΔM^ and TPP1^ΔRD^ (Supplementary Fig. 4g, h and 4j, k). In addition, we observed reduced localization of phosphorylated RPA32, CTIP and EXOI at telomeres in MCPH1-deleted cells expressing TPP1^ΔRD^, suggesting that MCPH1 is required to promote nuclease-dependent end resection to generate long tracts of ssDNA that can engage HDR^50^ (Supplementary Fig. 4l–o).

To confirm these results in another cell type, we knocked down MCPH1 using sh*MCPH1* in the telomerase negative U2OS cell (Supplementary Fig. 5a–c). In agreement with our data, only TPP1^ΔRD^ expression significantly increased the telomeric localization of endogenous MCPH1 (Supplementary Fig. 5d, e). While the number of γ-H2AX TIFs was unaffected by MCPH1 depletion in both TRF2^ΔBΔM^- and TPP1^ΔRD^-expressing cells, we found significant reduction in both 53BP1 and BARD1 TIFs in TPP1^ΔRD^-expressing cells after MCPH1 knockdown (Supplementary Fig. 5f–k), suggesting that MCPH1 has similar roles at dysfunctional telomeres of both telomerase-positive and negative cell lines. Moreover, TPP1^ΔRD^-induced RAD51 foci at telomeres were reduced by ~50% in MCPH1-depleted cells (Supplementary Fig. 5l, m). Together, these results suggest that MCPH1 is involved in the DDR at telomeres lacking POT1-TPP1, promoting CTIP and EXOI-mediated end resection and recruitment of p-RPA32, BARD1, and RAD51 to telomeres bearing unprotected 3’ single stranded overhangs.

Our previous data suggested that MCPH1^S333^ phosphorylation negatively affects the interaction of MCPH1 with TRF2 and reduces the localization of MCPH1 to both functional and dysfunctional telomeres (Fig. 2b–d). Therefore, we asked if the phosphorylation-regulated MCPH1–TRF2 interaction is required for the DDR at dysfunctional telomeres. To address this question, we reconstituted the MCPH1^Δ/Δ^ B2 cell line with either empty vector, WT MCPH1, MCPH1^S333A^, or MCPH1^S333D^ (Supplementary Fig. 6a). Reconstitution of WT MCPH1, MCPH1^S333A^, and MCPH1^S333D^ rescued the PCC phenotype in MCPH1^Δ/Δ^ cells, indicating that chromosome condensation does not require MCPH1’s localization to telomeres (Supplementary Fig. 6b). Overexpression of TPP1^ΔRD^ in reconstituted MCPH1^Δ/Δ^ cells revealed similar levels of γ-H2AX TIFs, confirming that telomere dysfunction was induced in all cell lines (Supplementary Fig. 6c–e). However, while MCPH1^Δ/Δ^ cells reconstituted with either WT MCPH1 or MCPH1^S333A^ displayed BARD1, p-RPA32, CTIP, and EXOI TIFs at similar levels to MCPH1^+/+^ cells, MCPH1^Δ/Δ^ cells reconstituted with either empty vector or with MCPH1^S333D^ showed a significant reduction in their ability to recruit these factors to telomeres (Fig. 3c–g and Supplementary Fig. 6f–h). Similarly, the reconstitution of MCPH1-depleted U2OS cells with either shRNA resistant WT MCPH1 or MCPH1^S333A^, but not with MCPH1^S333D^, restored TPP1^ΔRD^-induced RAD51 TIFs (Fig. 3h, i). Overall, these results suggest that MCPH1 promotes the recruitment of DNA damage factors to dysfunctional telomeres lacking POT1-TPP1 in a TRF2-dependent manner.

### MCPH1–TRF2 interaction promotes HDR at dysfunctional telomeres lacking POT1-TPP1

**Fig. 4:**
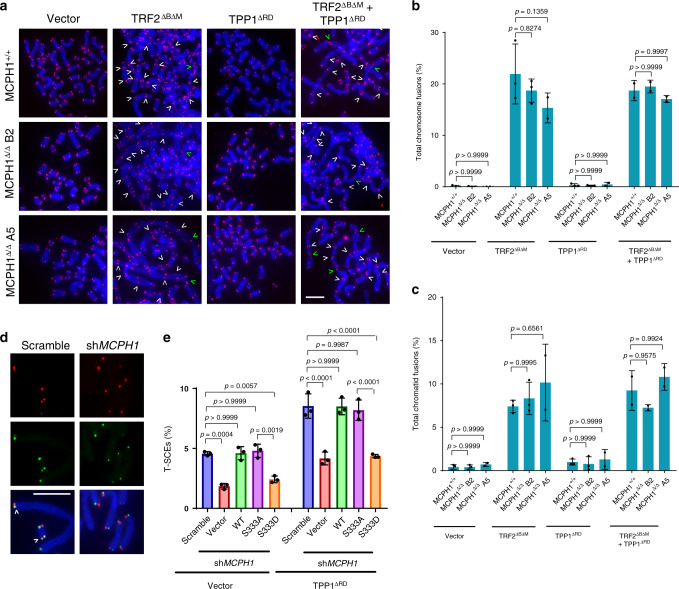
MCPH1–TRF2 interaction is required to promote HDR at telomeres lacking POT1-TPP1. **a** Telomeric PNA-FISH staining of metaphase spreads from MCPH1^+/+^, MCPH1^Δ/Δ^ B2, and MCPH1^Δ/Δ^ A5 HCT116 cells overexpressing either empty vector, TRF2^ΔBΔM^, TPP1^ΔRD^, or both TRF2^ΔBΔM^ and TPP1^ΔRD^. Telomeres were detected using a PNA probe (red) and DAPI was used to stain the chromosomes (blue). Representative images from either three (MCPH1^+/+^ and MCPH1^Δ/Δ^ B2 + vector, TRF2^ΔBΔM^, or TPP1^ΔRD^) or two (MCPH1^Δ/Δ^ A5 and samples with TRF2^ΔBΔM^ + TPP1^ΔRD^) independent experiments. White and green arrowheads indicate chromosome and chromatid fusions, respectively. Scale bar: 5 μm. **b**, **c** Quantification of the percentage of chromosome (**b**) and chromatid (**c**) fusions observed in metaphase spreads shown in (**a**). Data represent the mean values ± SD. *n* = 3 for MCPH1^+/+^ and MCPH1^Δ/Δ^ B2 + vector, TRF2^ΔBΔM^, and TPP1^ΔRD^; *n* = 2 for MCPH1^Δ/Δ^ A5 and samples with TRF2^ΔBΔM^ + TPP1^ΔRD^. A minimum of 30 metaphases for each sample were examined per experiment. Significance was determined using one-way ANOVA followed by Tukey’s multiple comparison test. **d** Representative images of telomere sister chromatid exchanges (T-SCEs) (arrowheads) from three independent CO-FISH experiments on metaphase spreads of U2OS cells expressing TPP1^ΔRD^ and either scrambled or *MCPH1* shRNA. Sister chromatid telomeres were labeled with a FAM-OO-(TTAGGG)_4_ PNA probe (green) and with a Cy3-OO-(CCCTAA)_4_ PNA probe (red) to detect telomeres generated by leading and lagging strand replication, respectively. Scale bar: 5 μm. **e** Quantification of the percentage of T-SCEs observed on metaphase spreads of U2OS cells expressing either empty vector or TPP1^ΔRD^ after MCPH1 depletion and reconstitution with the indicated constructs. Data are representative of the mean of three independent experiments ± SD. A minimum of 40 metaphases were analyzed per experiment. The indicated *p* values were calculated using one-way ANOVA followed by Tukey’s multiple comparison test.

We next analyzed the impact of MCPH1 deletion on DNA repair at telomeres through telomeric PNA-FISH analysis on metaphase spreads from MCPH1^+/+^ cells and from both MCPH1^Δ/Δ^ clones. Removal of endogenous TRF2 from telomeres, through overexpression of TRF2^ΔBΔM^, induced prominent C-NHEJ-mediated telomere fusions in both MCPH1^+/+^ and MCPH1^Δ/Δ^ cells (Fig. 4a–c and Supplementary Fig. 4e), while TPP1^ΔRD^ overexpression did not lead to telomeric fusions (Fig. 4a–c). Removal of both TRF2 and POT1-TPP1 promotes A-NHEJ-mediated repair of dysfunctional telomeres^40^. However, we did not observe any reduction in A-NHEJ-mediated chromosome and chromatid fusions in MCPH1^Δ/Δ^ cells when compared with MCPH1^+/+^ cells (Fig. 4a–c). These results suggest that MCPH1 deletion does not affect C-NHEJ or A-NHEJ dependent DNA repair at dysfunctional telomeres. Next, we sought to determine whether MCPH1 is required for HDR of telomeres lacking POT1-TPP1. A hallmark of HDR of telomeres is the generation of telomere sister chromatid exchanges (T-SCEs), which can be visualized via chromosome orientation FISH (CO-FISH) on metaphase spreads^51^. We performed CO-FISH in MCPH1 depleted U2OS cells and found a significant reduction of the number of T-SCEs observed in cells overexpressing either empty vector or TPP1^ΔRD^, suggesting that in the absence of MCPH1, HDR is compromised at dysfunctional telomeres (Fig. 4d, e). Reconstitution of MCPH1-depleted cells with either WT MCPH1 or MCPH1^S333A^ restored T-SCEs to levels similar to MCPH1^+/+^ cells, while reconstitution of MCPH1^S333D^ did not (Fig. 4e). Taken together, our results suggest that TRF2 recruits MCPH1 to dysfunctional telomeres lacking POT1-TPP1 to promote HDR.

### MCPH1 interacts with TRF2 in S phase to promote telomere replication

**Fig. 5:**
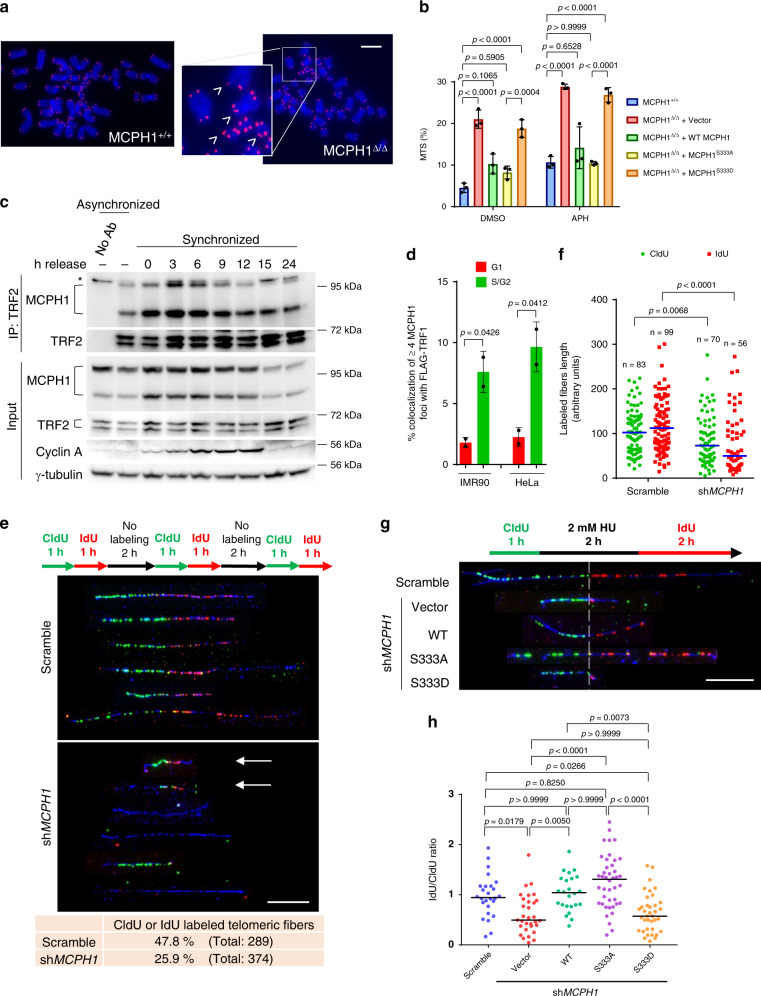
MCPH1 interacts with TRF2 in response to replication stress to promote replication fork progression at telomeres. **a** Representative images from three independent experiments of telomeric PNA-FISH on chromosome spreads of WT and MCPH1^Δ/Δ^ HCT116 cells to detect multiple telomeric signals (MTS) (arrowheads). Scale bar: 5 μm. **b** Mean values ± SD of the percentage of MTS visualized by PNA-FISH in the indicated cell lines treated with either DMSO or 0.25 μM aphidicolin (APH). *n* = three independent experiments. At least 50 metaphases were scored. One-way ANOVA followed by Tukey’s multiple comparison test. **c** Co-IP with anti-TRF2 antibody from lysates of synchronized U2OS cells harvested at the indicated time points. Cyclin A was used as a control for cell cycle progression. Representative blots from two independent experiments. No Ab: no antibody control; *non-specific band. See also Supplementary Fig. 7c. **d** Percentage of cells showing ≥4 MCPH1/FLAG-TRF1 co-localizations in IMR-90 and HeLa cells expressing CDT1 (G_1_) and Geminin (S/G_2_). See also Supplementary Fig. 7d. Mean values ± SD from two independent experiments are shown. At least 200 nuclei were scored. Two-sided Student’s *t* test. **e** SMARD analysis of telomeric DNA fibers in U2OS treated with either Scrambled or *MCPH1* shRNAs. Top: scheme of the CldU (green) and IdU (red) pulse label timing. Middle: representative images of telomeric fibers (telomeric DNA depicted in blue) from two independent experiments. Bottom: quantification of either CldU- or IdU-positive telomeric fibers. Scale bar: 10 μm. **f** Quantification of the length of CldU and IdU tracks from a representative experiment. Blue line: median. Two-sided Mann–Whitney test. **g** SMARD analysis of telomeric replication forks restart in U2OS cells treated with either Scrambled or *MCPH1* shRNA and reconstituted with the indicated constructs. Top: pulse labeling timing scheme with hydroxyurea (HU)-induced replication block. Bottom: Representative images from two independent experiments. The dashed line separates the CldU-labeled portion (regular replication) and the IdU-labeled portion (replication restart). Scale bar: 10 μm. **h** Quantification of the IdU/CldU length ratio for the fibers labeled with both halogenated nucleotides in one representative experiment. Black line: median. Kruskal–Wallis test with Dunn’s multiple comparison test.

While analyzing telomere signals on metaphase spreads of HCT116 cell lines, we noticed that both MCPH1^Δ/Δ^ clones displayed high levels of fragile telomeres (Supplementary Fig. 7a, b). Fragile telomeres appear as multiple telomeric signals (MTS) at chromatid ends and are indicative of telomere replication defects^52^. It has been previously shown that MCPH1 is involved in the replication stress response, promoting the recruitment of topoisomerase-binding protein 1 (TopBP1) to stalled replication forks^53^. To determine whether MCPH1 plays a role in telomere replication, we quantified the number of fragile telomeres in MCPH1^Δ/Δ^ cells reconstituted with either empty vector, WT MCPH1, MCPH1^S333A^, or MCPH1^S333D^. The elevated MTS levels observed in MCPH1^Δ/Δ^ cells decreased to WT levels after reconstitution with either WT MCPH1 or MCPH1^S333A^ (Fig. 5a, b). Expressing MCPH1^S333D^ was unable to reduce MTS to WT levels, suggesting that the MCPH1–TRF2 interaction is required to counteract replication stress at telomeres. Treatment with the DNA polymerase inhibitor aphidicolin (APH) further exacerbated the fragile telomere phenotype observed in both WT and reconstituted cell lines, suggestive of increased replication fork stalling at telomeres (Fig. 5b). To determine if the MCPH1–TRF2 interaction is cell-cycle regulated, we performed Co-IP experiments using an anti-TRF2 antibody in cell cycle synchronized U2OS cells at 3-hour time points after removal of double thymidine block. We verified proper cell cycle synchronization at each time point through propidium iodide flow cytometry analysis (Supplementary Fig. 7c) and further confirmed synchronization using immunoblot analysis to detect Cyclin A expression, which progressively increased from S to G_2_ and decreased during the M phase (Fig. 5c). Co-IP analysis revealed that MCPH1 interaction with TRF2 peaked at the 3-hour time point, suggesting that the MCPH1–TRF2 interaction occurs in S phase and gradually decreases while the cells enter the G_2_ phase of the cell cycle (Fig. 5c). To confirm this result, we analyzed MCPH1 telomeric localization in G_1_ and S/G_2_ cells with the FUCCI system, which uses the expression of cell-cycle specific fluorescent proteins to distinguish cells at different phases of the cell-cycle^54^. In both IMR-90 and HeLa cells, we found that MCPH1 co-localizes with FLAG-tagged TRF1 mainly in Geminin-positive cells (S/G_2_) rather than cells expressing CDT1 (G_1_), suggesting that MCPH1 localizes at telomeres during S/G_2_ (Fig. 5d and Supplementary Fig. 7d). Moreover, we analyzed the telomeric localization of endogenous MCPH1 using immunostaining and PNA-FISH in synchronized IMR-90 and HeLa cells, measuring Cyclin A staining intensity to verify cell cycle synchronization (Supplementary Fig. 7e, f and h, i). We found that MCPH1 telomeric localization peaks after 3 hours from the release of the double thymidine block and then gradually decreases following a pattern that is compatible with our Co-IP data in U2OS (Supplementary Fig. 7g, j).  Together, these results suggest that MCPH1 is recruited to telomeres preferentially during the S phase.

To determine whether MCPH1 plays a role in telomere replication, we performed single molecule analysis of replicated DNA (SMARD) on U2OS cells, which possess the long telomeres necessary for this assay, and used a (CCCTAA)_4_ PNA probe to detect telomeric DNA fibers^52,55^. The cells were transfected with either scrambled or *MCPH1* shRNA and sequentially pulse-labeled three times with CldU and IdU for 1 h each, followed by a 2 h chase without halogenated nucleotides^52^ (Fig. 5e). As expected, CldU and IdU tracks of telomeric fibers from WT cells had comparable length, suggesting that the fork progression rate was consistent through time. However, we found that replicating telomeric DNA molecules from MCPH1-depleted cells displayed CldU and IdU segments that on average were 2-fold shorter than those observed in control cells (Fig. 5e, f). The presence of very short CldU and IdU tracks at the telomeres of MCPH1-depleted cells (Fig. 5e, arrows) suggests defects in replication fork progression at telomeres, likely due to increased replication fork stalling. To verify this hypothesis, we used SMARD analysis to look at telomeric replication fork restart after hydroxyurea (HU) treatment. For this assay, replication was blocked with HU after the CldU treatment, and then cells were labeled with IdU after HU removal (Fig. 5g). In these conditions, the IdU/CldU length ratio is indicative of the efficiency of replication fork restart after HU removal. MCPH1 depletion resulted in a significantly lower IdU/CldU ratio compared to control cells, suggesting that in the absence of MCPH1 replication fork restart is delayed (Fig. 5g, h and Supplementary Fig. 7k, l). Reconstitution with either WT MCPH1 or MCPH1^S333A^ restored the IdU/CldU ratio to levels comparable to those of the control cells, while MCPH1^S333D^ showed IdU/CldU values similar to MCPH1-depleted cells (Fig. 5h). These results suggest that MCPH1 is recruited to telomeres in response to replication stress to promote stalled replication fork restart in a TRF2-dependent manner.

Many HDR factors play important roles in maintaining the integrity of stalled forks, preventing fork collapse that would otherwise lead to the generation of DSBs, and promoting replication fork restart^56–58^. Replication stress at telomeres is counteracted by TRF1, which recruits the helicase BLM to unwind DNA secondary structures that can be formed by the G-rich telomeric repeats^52,59^. To determine if MCPH1 localized to telomeres under replication stress, we depleted TRF1 in MCPH1^+/+^ and MCPH1^Δ/Δ^ cells reconstituted with either empty vector, WT MCPH1, MCPH1^S333A^, or MCPH1^S333D^ to induce replication fork stalling at telomeres. MCPH1 localization to telomeres lacking TRF1 requires interaction with TRF2, since MCPH1^S333D^ telomeric foci in TRF1-depleted cells were dramatically reduced in comparison to WT MCPH1 and MCPH1^S333A^ (Supplementary Fig. 7m, n). Together, these results suggest that MCPH1 localizes at stalled replication forks at telomeres in a TRF2-dependent manner, counteracting telomeric replication stress to promote replication fork progression through telomeric repeats.

### MCPH1 promotes the restart of stalled replication forks and counteracts genomic replication stress

**Fig. 6:**
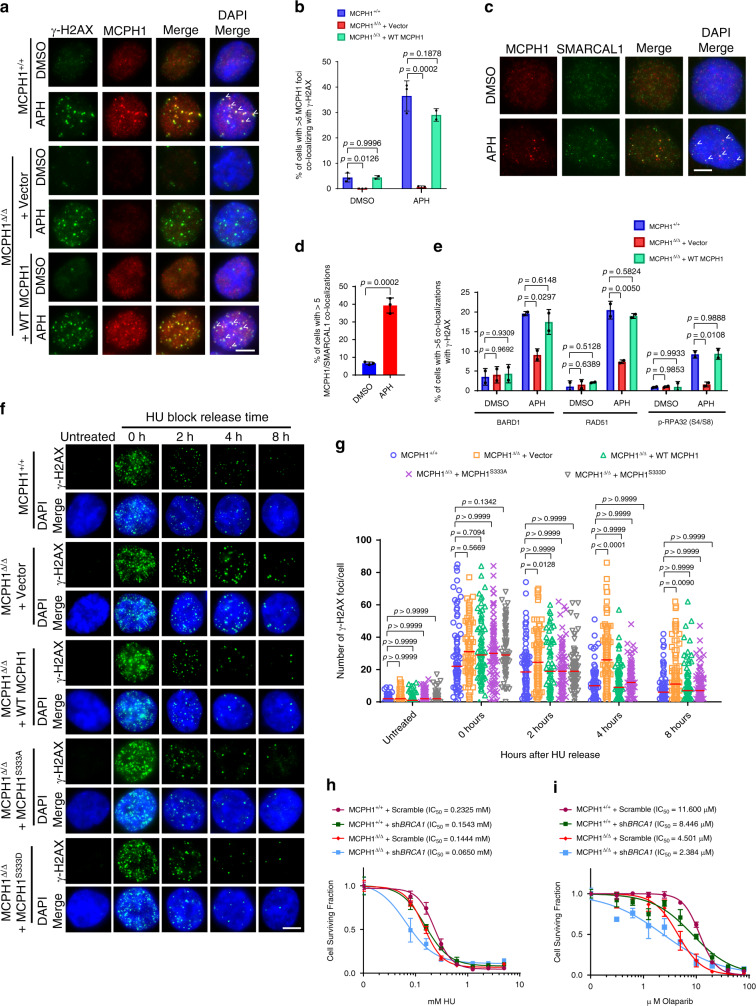
MCPH1 is required for recruitment of HDR factors to stalled replication forks to promote fork restart. **a** MCPH1 (red) and γ-H2AX (green) immunostaining in the indicated cells lines treated with either DMSO or 0.25 μM APH. Representative images from either three (MCPH1^+/+^ and MCPH1^Δ/Δ^ + vector cells) or two (MCPH1^Δ/Δ^ + WT MCPH1 cells) independent experiments. **b** Mean values ± SD of the percentage of cells with >5 MCPH1/γ-H2AX co-localizations from (**a**). *n* = 3 for MCPH1^+/+^ and MCPH1^Δ/Δ^ + vector cells, *n* = 2 for MCPH1^Δ/Δ^ + WT MCPH1 cells. At least 200 nuclei were scored. One-way ANOVA followed by Tukey’s multiple comparison test. **c** MCPH1 (red) and SMARCAL1 (green) immunostaining in U2OS cells treated with either DMSO or 0.25 μM APH. Representative images from three independent experiments. **d** Mean values ± SD of the percentage of cells with >5 co-localizing MCPH1/SMARCAL1 foci from (**c**). *n* = 3., at least 200 cells scored. Two-sided Student’s *t* test. **e** Percentage of cells with >5 BARD1, RAD51 or p-RPA32 (S4/S8) foci co-localizing with γ-H2AX in the indicated cell lines treated with either DMSO or 0.25 μM APH. Representative images are shown in Supplementary Fig. 8a. Data represent mean values ± SD, *n* = 2. At least 200 nuclei were examined. One-way ANOVA followed by Tukey’s multiple comparison test. **f** Representative images from two independent time course experiments to assess γ-H2AX foci (green) resolution in the indicated cell lines after release of HU block. Cells were incubated with 2 mM HU for 24 h before replacing the media and then fixed at the indicated time points. Untreated cells were used as control. **g** Number of γ-H2AX foci observed in 50–100 cells from each sample shown in (**f**). Data from one representative experiment. Red line: median. Kruskal–Wallis test followed by Dunn’s multiple comparison test. **h**, **i** Cell viability assay of MCPH1^+/+^ and MCPH1^Δ/Δ^ cells treated with increasing amount of HU (**h**) or Olaparib (**i**), with or without concomitant BRCA1 depletion. Data from one of three independent experiments. Statistical significance is shown in Supplementary Table 1 (HU) and 2 (Olaparib). Scale bars for **a**, **c**, **f**: 5 μm.

To better understand MCPH1’s role at stalled replication forks, we analyzed the genomic localization of MCPH1 in HCT116 cells treated with aphidicolin. Stalled replication forks accumulate γ-H2AX, and we detected MCPH1 foci co-localizing with γ-H2AX foci in both MCPH1^+/+^ and MCPH1^Δ/Δ^ cells reconstituted with WT MCPH1 (Fig. 6a, b). We also verified MCPH1 co-localization with the annealing helicase SMARCAL1, which localizes to stalled replication forks to maintain fork integrity^60,61^ (Fig. 6c, d). These results suggest that MCPH1 is recruited to stalled replication forks throughout the genome. We next addressed MCPH1’s role in the recruitment of HDR factors at APH-induced stalled replication forks by visualizing their co-localization with γ-H2AX. Compared to MCPH1^+/+^ cells, we found that the localization of BARD1, RAD51, and p-RPA32 to stalled replication forks was significantly reduced in MCPH1^Δ/Δ^ cells. Reconstitution with WT MCPH1 restored BARD1, RAD51, and p-RPA32 foci formation to near WT levels (Fig. 6e and Supplementary Fig. 8a), suggesting that MCPH1 is required to promote the recruitment of HDR factors to ssDNA found at stalled replication forks. Consistent with a role of MCPH1 in promoting end resection, we found a reduction of both CtIP and EXOI recruitment to stalled forks in U2OS cells after MCPH1 depletion (Supplementary Fig. 8b). MCPH1-depleted cells also showed a reduction of SMARCAL1 recruitment to stalled replication forks (Supplementary Fig. 8c), consistent with RPA’s known role in promoting SMARCAL1 localization to stalled forks^60^.

Our data suggest that MCPH1 facilitates the recruitment of HDR factors to stalled forks but is not strictly required for this process. Indeed, loss of MCPH1 reduced the formation of foci for HDR factors at stalled forks but did not completely abolish it. To elucidate this further, we analyzed the kinetics of stalled replication fork restart in MCPH1^+/+^ and MCPH1^Δ/Δ^ cells by monitoring the localization of BRCA1, which accumulates at stalled forks to promote fork protection and restart^62–64^. We found an increased accumulation of BRCA1 foci after HU treatment over 4 h in MCPH1^+/+^ cells. In contrast, MCPH1^Δ/Δ^ cells showed significantly  fewer BRCA1-positive foci at each time point examined, suggesting a delayed recruitment of BRCA1 to stalled replication forks in the absence of MCPH1 (Supplementary Fig. 8d). HU treatment results in stalled replication forks that accumulate γ-H2AX foci. These foci gradually disappeared over time after HU removal, presumably due to replication fork restart^65^. While MCPH1^+/+^ cells displayed a rapid reduction in the number of γ-H2AX foci after HU removal, γ-H2AX foci in MCPH1^Δ/Δ^ cells persisted and only started to decrease 8 h after HU release (Fig. 6f, g). These results suggest that in the absence of MCPH1, replication fork restart is delayed. Reconstitution of MCPH1^Δ/Δ^ cells with either WT MCPH1, MCPH1^S333A^ or MCPH1^S333D^ restored the kinetics of γ-H2AX foci resolution to levels similar to those observed in MCPH1^+/+^ cells, indicating that MCPH1 role at genomic stalled replication forks is not dependent on its ability to interact with TRF2 (Fig. 6f, g). Consistent with these results, MCPH1^Δ/Δ^ cells showed increased sensitivity to replication stress-inducing reagents, including HU and Olaparib, when compared to MCPH1^+/+^ cells (Fig. 6h, i and Supplementary Tables 1 and 2). Interestingly, depletion of BRCA1 in MCPH1^Δ/Δ^ cells further reduced cell survival after HU and Olaparib treatment, suggesting a synergistic role for both MCPH1 and BRCA1 in suppressing replication stress. Overall, our results highlight MCPH1’s role in recruiting HDR factors to stalled replication forks to promote fork restart, counteracting genomic replication stress.

## Discussion

**Fig. 7:**
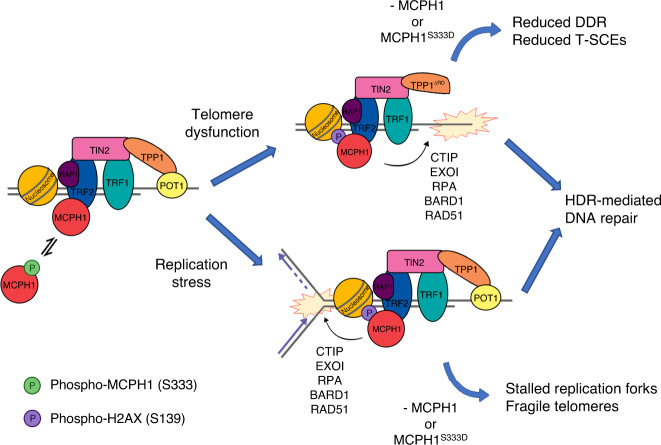
Schematic depicting MCPH1’s role at telomeres. MCPH1 interacts with TRF2 when S333 is de-phosphorylated, while this interaction is disrupted upon S333 phosphorylation. In unperturbed conditions, MCPH1 exists in equilibrium between the phosphorylated and the de-phosphorylated forms and its telomeric localization increases in S phase. Removal of POT1 through the overexpression of TPP1^ΔRD^ increases MCPH1 localization to telomeres that is dependent upon the interaction with both TRF2 and γ-H2AX. MCPH1 binding to TRF2 promotes the recruitment of HDR and end resection factors to initiate HDR at telomeres lacking POT1-TPP1. Cells lacking MCPH1 or expressing MCPH1^S333D^ display reduced recruitment of HDR factors and reduced T-SCEs, suggestive of HDR defects. Similarly, induction of replication stress at telomeres increases MCPH1 telomeric localization, promoted by its interaction with TRF2. In the absence of MCPH1 or upon overexpression of the phosphomimetic mutant MCPH1^S333D^, replication forks stalling at telomeres increases and telomere replication is impaired, resulting in telomere fragility. These observations suggest that telomeric localization of MCPH1 is required for proper telomere replication by promoting stalled replication fork restart.

In this study, we have identified and characterized the roles of MCPH1 at telomeres. We have shown that MCPH1 specifically interacts with TRF2^TRFH^ on functional telomeres, consistent with a previous report^42^, and that this interaction may be modulated by the phosphorylation of MCPH1^S333^. MCPH1 interacts with both TRF2 (via the TBM) and γ-H2AX (via the C-terminal BRCT domains) to localize to dysfunctional telomeres lacking POT1-TPP1, to promote DDR and DNA repair through HDR, analogous to its well-known role at genomic DNA damage sites^15–18,20,21^. It has been hypothesized that MCPH1 promotes the recruitment of DDR factors at DSBs by interacting with the ATP-dependent SWI-SNF chromatin remodeling complex and generating a more accessible chromatin environment^17^. However, while MCPH1 localization to genomic DSBs and to dysfunctional telomeres occurs through its interaction with γ-H2AX^18^, MCPH1’s localization to functional telomeres is strictly dependent on its interaction with TRF2. MCPH1-mediated DDR factors recruitment, end resection, and HDR of telomeres lacking POT1-TPP1 require binding to both TRF2 and γ-H2AX, suggesting an additional means to regulate MCPH1 function at dysfunctional telomeres compared to genomic DSBs (Fig. 7, top). While MCPH1 has been reported to contribute to C-NHEJ repair at DSBs^17,21^, our data show that MCPH1 loss does not compromise C-NHEJ-mediated repair of dysfunctional telomeres lacking TRF2 (Fig. 4a–c). However, we cannot rule out the possibility that MCPH1 is not involved in TRF2^ΔBΔM^-induced chromosome fusions simply because there are no TRF2 molecules at telomeres for it to interact with, as indicated by the poor MCPH1 localization to telomeres in these cells. Similarly, in the absence of MCPH1, we did not detect any significant difference in A-NHEJ-mediated repair of dysfunctional telomeres lacking both TRF2 and POT1-TPP1 (Fig. 4a–c).

Beside its role in promoting HDR at dysfunctional telomeres, we found a previously unrecognized function of MCPH1 necessary to repress replication stress at telomeres in a TRF2-dependent manner (Fig. 7, bottom). Telomeres experience elevated replication stress, since the repetitive nature of telomeric DNA and its propensity to form secondary structures can easily stall the replication fork^66^. In the absence of MCPH1, telomeric replication forks proceed at a slower rate and restart of stalled replication forks is delayed. In addition, MCPH1 co-localizes with SMARCAL1 in response to replication stress and readily localizes to TRF1-depleted telomeres, further suggesting that MCPH1 recognizes stalled replication forks both at genomic DNA and at telomeres. Our data also show that MCPH1 promotes end resection and recruitment of HDR factors to genomic stalled replication forks in order to restart replication. In MCPH1 deficient cells, restart of stalled replication forks is not abolished, but takes longer time, suggesting the contribution of a MCPH1-independent replication pathway that requires BRCA1. Our telomere SMARD analysis shows that, similar to its role at stalled forks in genomic DNA, MCPH1 promotes restart of stalled replication forks at telomeres in a TRF2-dependent manner (Fig. 5g, h), facilitating fork progression through telomeric repeats. In the absence of MCPH1, replication stress at telomeres likely results in increased replication fork stalling and the activation of an ATR-dependent DDR that can lead to either cellular senescence or apoptosis. The sensitivity of MCPH1-deficient cell lines to PARP inhibition and to HU treatment has clinical implications for the treatment of tumors harboring MCPH1 mutations. Olaparib has been successfully used to treat BRCA1-deficient tumors since the combined effects of PARP inhibition and BRCA1 deficiency can mediate synthetic lethality in many cancers^67,68^. Our data shows that MCPH1 knockout caused even higher sensitivity to Olaparib than BRCA1 knockdown, suggesting that PARP inhibition coupled with MCPH1 deficiency may be synthetic-lethal. Interestingly, COSMIC database revealed three MCPH1^TBM^ specific missense mutations (R331C, S333C, and P334L) in cancer patients^69–71^, further suggesting the notion that disruption of the MCPH1–TRF2 interaction may promote tumorigenesis.

Our data show that human MCPH1 interacts with TRF2^TRFH^ through a canonical TBM that is not conserved in mouse cells, similarly to what has been previously shown for SLX4^72^. TBM-containing proteins compete for the same surface on the TRF2^TRFH^ domain, and we have previously shown that post-translational modifications of specific TBM amino acid residues can coordinate the interaction of several proteins involved in DNA repair in a cell cycle dependent manner^45^. Similar to NBS1^TBM^ ^45^, phosphorylation of MCPH1^TBM^ at S333 sterically prevents it from fitting within the hydrophobic pocket of TRF2^TRFH^. In vivo phosphorylation of MCPH1^S333^ has been previously identified in large-scale phosphorylation proteomics screenings^73–76^. Our data reveal an increase in the MCPH1–TRF2 interaction during S-phase, suggesting that MCPH1^S333^ is likely de-phosphorylated during S-phase to promote TRF2-mediated recruitment to replicating telomeres. These results highlight a central role of TRF2^TRFH^ in coordinating both DNA damage signaling repression and replication fork progression at functional telomeres. While it has been hypothesized that TRF1 is the only shelterin protein involved in repressing replication stress at telomeres^30^, increasing evidence suggest that TRF2 also contributes to telomere replication. Indeed, TRF2 is responsible for recruiting several factors required for proper telomere replication, including RTEL1^77,78^, the replisome proteins Claspin, PCNA, DONSON, Timeless^40,79,80^, the nuclease Apollo/SNM1B^81–83^, and now MCPH1. Moreover, TRF2 has been shown to recruit Topoisomerase 2α at telomeres and to regulate DNA topology both at telomeres and centromeres^84–86^. Together, these data suggest that both TRF1 and TRF2 play important roles in coordinating replication of telomeric DNA.

MCPH1 loss-of-function mutations have been linked to primary microcephaly^8^, but the underlying molecular mechanism by which these mutations influence pathogenesis is unknown. Several studies tried to address this problem generating MCPH1-deficient mice, even though these mice did not always develop microcephaly^20,87–91^. Mice with microcephaly displayed a reduction in the size of the cerebral cortex at birth associated with increased NPCs apoptosis, suggesting that MCPH1 plays a role in the survival of cortical progenitors^88–90^. In these mice, NPCs exhibit radiation hypersensitivity^20,89^, consistent with a heightened requirement for the HDR pathway in these cells^92^. A strikingly similar phenotype has been observed in TopBP1-deficient mice, in which NPCs accumulate replication stress-induced DNA damage^93^. Moreover, mutations in DONSON are associated with replication and cell-cycle checkpoint defects, which lead to microcephalic dwarfism^5^. We have recently shown that DONSON forms a complex with the replisome proteins Claspin and Proliferating Cell Nuclear Antigen (PCNA), and that this complex interacts with TRF2 to promote telomere replication and the generation of the 3’ ss overhang after replication^40^. This complex also participates in both A-NHEJ and HDR pathways at dysfunctional telomeres^40^. Our data suggest that replication stress at telomeres is a major consequence of MCPH1 deletion and speculate that telomere fragility associated with MCPH1 mutations might contribute to the neuronal developmental defects. Due to their highly repetitive nature and their propensity to adopt aberrant secondary structures, including G-quadruplexes, telomeres represent significant challenges for the DNA replication machinery^66^. Replication stress at telomeres can lead to fork collapse and generation of DSBs, resulting in chromosomal aberrations and rearrangements^94^. Indeed, microcephaly is often associated with rearrangements and deletions in telomeric and subtelomeric regions, suggesting that telomeric defects may negatively affect neuronal development^95^. Moreover, primary microcephaly is also a key clinical feature of the Hoyeraal-Hreidarsson syndrome, a telomere disorder caused by mutations in shelterin or in telomerase, the ribonucleoprotein complex that synthesizes the telomeric repeats^96,97^. Reduced telomere length and telomerase deficiency adversely affect NPC proliferation, resulting in the depletion of this cellular population^98,99^. These results suggest that proper telomere maintenance is essential to maintain the proliferative capacity of NPCs. In support of this notion, telomere end-protection is critical for NPC survival during brain development, as removal of either TRF2 or POT1a elicits DNA damage signaling and massive cell death in neuronal stem cells of developing murine brains^100–102^. Taken together, these observations suggest that proper telomere function is required for proliferation of neuronal progenitors and neurogenesis, and further support our hypothesis that telomere dysfunction may underlie neural development defects in MCPH1 patients bearing mutated MCPH1. Interestingly, a recent study reported that MCPH1 localizes only in the cytoplasm of murine NPCs, while it also expresses in the nuclei of progenitor cells in developing human brains^90^. This observation suggests that MCPH1 could have additional nuclear functions in human NPCs during development, and it correlates with our data showing the telomeric role of MCPH1 only in human cells.

## Methods

### Protein crystallization, data collection, and structure determination

TRF2^TRFH^ (residues 42–245) and MCPH1^TBM^ (residues 322–342) peptides were expressed in *E. coli* Rosetta cells using a modified pET28b vector with a SUMO protein fused at the N-terminus after the 6XHis tag^43,45^. After induction for 16 h with 0.1 mM IPTG at 25 °C, the cells were harvested by centrifugation and the pellets were resuspended in lysis buffer (50 mM Tris-HCl, pH 8.0, 50 mM NaH_2_PO_4_, 400 mM NaCl, 3 mM imidazole, 10% glycerol, 1 mM PMSF, 0.1 mg/ml lysozyme, 2 mM 2-mercaptoethanol, and home-made protease inhibitor cocktail). The cells were then lysed by sonication and the cell debris was removed by ultracentrifugation. The supernatant was mixed with Ni-NTA agarose beads (Qiagen) and rocked for 6 h at 4 °C before elution with 250 mM imidazole. Then Ulp1 protease was added to remove the His-SUMO tag. After Ulp1 digestion, the TRF2^TRFH^ and the MCPH1^TBM^ peptides were further purified by gel-filtration chromatography on Hiload Superdex75 column (GE Healthcare) equilibrated with buffer A (25 mM Tris-HCl, pH 8.0, 150 mM NaCl and 5 mM DTT) and buffer B (100 mM ammonium bicarbonate), respectively. TRF2^TRFH^ and MCPH1^TBM^ were mixed at a molar ratio of 1:2 prior to crystallization. The complex was crystallized in the buffer with 100 mM CHES-NaOH, pH 9.5, 30% PEG400 at 293K. The crystals were harvested in the same buffer with 20% glycerol. The 2.15 Å dataset was collected at the beamline BL19U1 of the Shanghai Synchrotron Radiation Facility and processed using HKL3000^103^. The crystal belongs to *P2*_*1*_*2*_*1*_*2*_*1*_ space group. The structure of TRF2^TRFH^–MCPH^TBM^ was solved by molecular replacement with Phaser^104^ using TRF2^TRFH^ (PDB ID: 1H6P) structure as the searching model. Crystallography refinement was performed with Phenix^105^ together with manual model building in Coot^106^.

### Isothermal titration calorimetry

The equilibrium dissociation constants of the WT and mutant TRF2^TRFH^–MCPH1^TBM^ interactions were determined using a MicroCal iTC_200_ calorimeter (Malvern Panalytical). MCPH1^TBM^ peptides, synthesized by GenScript China, at 1.0 mM concentration in the syringe were titrated into TRF2^TRFH^ at 0.1 mM concentration in the sample cell. The enthalpies of binding between the TRF2^TRFH^ domain and the MCPH1^TBM^ were measured at 20 °C in 25 mM Tris-HCl (pH 8.0) and 150 mM NaCl. ITC data were subsequently analyzed and fitted using Origin 7 software (OriginLab) with blank injections of peptides into buffer subtracted from the experimental titrations prior to data analysis.

### Plasmids and reagents

MCPH1 point mutations were generated by PCR from pCMV6-FLAG-hMCPH1 or pCMV6-FLAG-hMCPH1^ΔBRCT^ constructs^16^. For viral constructs, WT and mutant MCPH1 sequences were sub-cloned in the retrovirus vector pQXCIP puro (Clontech). pYX-ASC-mMCPH1 was purchased from Transomic and mMCPH1 sequence was sub-cloned in pQXCIP puro with a Myc-tag at the N-terminus. pSuper.retro MCPH1 shRNA^16^ was used to knockdown MCPH1 in U2OS cells. pLKO.1 *TRF1* (TRCN0000040162) and pLKO.1 *TRF2* (TRCN0000280026) shRNAs were purchased from Sigma. pCDNA3.1 Myc-hTRF2 and pCDNA3.1 Myc-hTRF2^F120A^ were used for Co-IP with hMCPH1^38,45^. pBabe puro Myc-hTRF2^ΔBΔM^ and pQXCIP puro hTPP1^ΔRD^ (either FLAG- or HA-tagged) were used to remove endogenous TRF2 and POT1-TPP1, respectively^45^. pQXCIP puro FLAG-mTIN2^A110R^ was used to induce telomere dysfunction in MEFs^31^. pQXCIP puro HA-RPA32 was used to overexpress RPA32 in HCT116. mKO1-hCDT1 and mAG1-hGeminin were used to detect G_1_ and S/G_2_ cells, respectively^54^. pLPC FLAG-hTRF1 was used for Co-IP with MCPH1 and to detect telomeres in FUCCI-transfected cells. Rabbit monoclonal antibody against MCPH1/BRIT1 was purchased from Cell Signaling Technology (#4120, 1:1000 dilution). Antibodies that recognize phosphorylated γ-H2AX (Millipore #05-636, 1:1000 dilution), 53BP1 (Santa Cruz #sc-22760, 1:1000 dilution), BARD1 (Santa Cruz #sc-11438, 1:1000 dilution), BRCA1 (Santa Cruz #sc-6954, 1:1000 dilution), RAD51 (Santa Cruz #sc-8349, 1:500 dilution), phosphorylated RPA32 (S4/S8) (Bethyl #A300-245A, 1:500 dilution), phosphorylated RPA32 (S33) (Bethyl #A300-246A, 1:1000 dilution), CTIP (Santa Cruz #sc-22838, 1:1000 dilution), EXOI (Santa Cruz #sc-33194, 1:500 dilution) and SMARCAL1 (Santa Cruz #sc-376377, 1:500 dilution) were used for the DNA damage assays. Mouse monoclonal anti‐TRF2 (Millipore #05‐521, 1:1000 dilution) and Protein A/Protein G Sepharose beads (GE Healthcare #17-6002-35) were used to pull down endogenous TRF2 in U2OS cells. Mouse anti-Cyclin A antibody (Santa Cruz #sc-239, 1:500 dilution) was used as a control for cell synchronization. Anti‐epitope tag antibodies were purchased from Sigma (anti‐FLAG #F3165, 1:2000 dilution) or Millipore (anti‐Myc #05‐724, 1:2000 dilution). Mouse anti-γ-tubulin antibody (Sigma #T6557, 1:5000 dilution) was used for the internal control in western blots. Secondary antibodies for western blot: peroxidase-linked anti-mouse IgG (Amersham NXA931V, 1:5000 dilution), peroxidase-linked anti-rabbit IgG (Amersham NA934V, 1:5000 dilution). Secondary antibodies for immunostaining were purchased from Invitrogen and used at a 1:2000 dilution: Alexa Fluor 488 anti-mouse (A11001), Alexa Fluor 568 anti-mouse (A11004), Alexa Fluor 488 anti-rabbit (A11008), Alexa Fluor 594 anti-rabbit (A11012). Thymidine, Propidium Iodide, aphidicolin, hydroxyurea, MG132 and doxorubicin were purchased from Sigma. Olaparib was purchased from Selleckchem.

### Cell lines and generation of MCPH1^Δ/Δ^ cell lines using CRISPR/Cas9

293T, HeLa, IMR-90, WT MEFs and *H2AX*^*-/-*^ MEFs were cultured in DMEM supplemented with 10% FBS and maintained in 5% CO_2_ at 37 °C. HCT116 and U2OS cells were cultured in McCoy’s 5 A medium. To obtain the MCPH1^Δ/Δ^ cell lines, the MCPH1 sgRNA oligonucleotide (Supplementary Table 3) was inserted into the lentiCRISPRv2 vector^107^. For viral infection, DNA constructs were transfected into 293T cells using Fugene 6 (Promega) and packaged into retro or lentiviral particles. Viral supernatant was collected 48–72 hours after transfection, filtered and directly used to infect the target cells.

### Western blot analysis

Trypsinized cells were lysed in urea lysis buffer (8 M urea, 50 mM Tris-HCl, pH 7.4, and 150 mM β-mercaptoethanol). The lysates were denatured and then resolved on SDS-PAGE gel. The separated proteins were then blotted on a nitrocellulose plus membrane (Amersham), blocked with blocking solution (5% non-fat dry milk in PBS/0.1% Tween-20) for at least 1 h and incubated with appropriate primary antibody in blocking solution at least 2 h at room temperature or overnight at 4 °C. The membranes were washed 3 × 5 min with PBS/0.1% Tween-20 and incubated with appropriate secondary antibody in blocking solution for 1 h at room temperature. Chemiluminescence detection was performed using an ECL Western Blotting Detection kit from GE Healthcare. Uncropped western blots are shown in Source data file.

### Co-immunoprecipitation

293T cells grown in 100 mm plates were co-transfected with either FLAG-MCPH1 or untagged MCPH1 constructs and either Myc-hTRF2, Myc-hTRF2^F120A^ or FLAG-hTRF1 and vector controls. FLAG-MCPH1^ΔBRCT^-expressing cells were treated with 12.5 μM of the proteasome inhibitor MG132 to stabilize this mutant. The same treatment was performed in FLAG-WT MCPH1 expressing cells as a control. 48 hours after transfection, cells were harvested and lysed in BC300 buffer (20 mM HEPES, pH 7.5, 300 mM KCl, 10% glycerol, 1 mM EDTA, 0.5% (v/v) NP-40). Supernatants were incubated with Myc antibody-conjugated agarose beads (Sigma) for 4 h and the supernatant was removed after centrifugation at 400 × *g* for 2 min. Beads were washed thrice with BC300 buffer and eluted proteins analyzed by SDS-PAGE.

### Immunofluorescence and fluorescent in situ hybridization

Cells grown on coverslips were fixed for 10 min in 2% (w/v) sucrose and 2% (v/v) paraformaldehyde at room temperature followed by PBS washes. Coverslips were blocked in 0.2% (w/v) fish gelatin and 0.5% (w/v) BSA in PBS. Cells were incubated with primary antibodies and after PBS washes, cells were incubated with appropriate Alexa fluor secondary antibodies followed by washes in PBS + 0.1% Triton. IF-FISH was carried out using a 5′-Cy3-OO-(CCCTAA)_4_-3′ PNA telomere probe (PNA Bio). DNA was stained with DAPI, and digital images captured using Metamorph (Molecular Devices) with a Nikon Eclipse 800 microscope and an Andore CCD camera. Raw quantification data of immunofluorescence experiments are shown in Source data file.

### Cell cycle analysis

Exponentially growing U2OS, IMR-90, and HeLa cells were subjected to 2 mM thymidine containing medium for 14 h followed by three PBS washes and release into fresh medium for 11 h. Cells were arrested a second time in 2 mM thymidine for 14 h and then washed in PBS thrice before release into fresh medium and either harvested for Co-IP or fixed for immunostaining at 3 h-time points for the next 24 h. A portion of the cells was fixed in 70% ice-cold ethanol for at least 24 h at −20 °C, washed twice with PBS and then resuspended in 1 ml PBS containing 50 μg/ml of Propidium Iodide and 100 μg/ml of RNAse A. After incubation at 4 °C overnight, the samples were analyzed on a BD LSR Fortessa cytometer. For Co-IP, cell lysates were incubated with an anti-TRF2 antibody overnight and with protein G and protein A sepharose beads for 4 h at 4 °C to pull down endogenous TRF2. Immunostaining was performed as described above. The FUCCI system was used to distinguish cycling G1 and S/G2 cells, based on the expression of the fluorescent proteins mKO1-hCDT1 and mAG1-hGeminin, respectively^54^. FUCCI-transfected cells were fixed for immunofluorescence and stained as described above.

### Chromosome analysis by telomere PNA-FISH and CO-FISH

Cells were treated with 0.5 µg/ml of Colcemid before harvest. Cells were pelleted by centrifugation at 600 × *g* for 8 min. Cell pellets were resuspended in 0.06 M KCl, incubated for 15 min at room temperature and washed three times with methanol: acetic acid (3:1 ratio). Metaphase spreads were prepared on microscope slides, treated with 0.5 mg/ml of RNAse A (Sigma) for 10 minutes at 37 °C and fixed with 3% formalin in PBS for 10 min at room temperature. The samples were denatured at 85 °C for 3 min and telomere PNA-FISH performed incubating the slides with a 5′-Cy3-OO-(CCCTAA)_4_-3′ probe (PNA Bio) in hybridization buffer (0.5 μg/ml tRNA, 1 mg/ml BSA, 0.06 × SSC, 70% formamide) at room temperature overnight in a humid chamber^39,108^. For CO-FISH, cells were incubated with 10 μM BrdU for 12 h, treated with 0.5 μg/ml of Colcemid for 4 h and harvested. Formalin-fixed metaphase spreads were stained with 0.5 μg/ml of Hoechst 33258 (Sigma) in 2 × SSC for 15 min at room temperature before being exposed to UV light equivalent to 5.4 × 10^3^ J/m^2^. After digestion with 200 U of Exonuclease III (Promega), the samples were denatured at 85 °C for 3 min and incubated sequentially with 5′-Cy3-OO-(CCCTAA)_4_-3′ and 5′-FAM-CO-(TTAGGG)_4_-3′ probes as described above. Images were captured as described above. The percentage of telomere aberrations (telomeric fusions, T-SCEs, fragile telomeres) observed is defined as: total number of telomere aberrations in 30–50 metaphase spreads analyzed divided by the total number of chromosomes examined × 100%. Raw quantification data are included in Source data file.

### Quantitative reverse transcription PCR analysis

U2OS cells transfected with either Scrambled or *MCPH1* shRNA were lysed and total RNA was extracted using TRIzol reagent (Invitrogen). Reverse transcription was performed using iScript cDNA Synthesis kit (Bio-Rad). The iTaq Universal SYBR Green Supermix (Bio-Rad) was used for quantitative PCR with three different sets of primers for MCPH1 while actin-specific primers were used for the internal control (Supplementary Table 3). The qPCR run was performed using an Applied Biosystems StepOnePlus Real-Time PCR System. The fold change and the standard error were calculated from the triplicate C_t_ values using the 2^−ΔΔCt^ method. Raw Ct values and data analysis are shown in Source data file.

### Telomere length analysis

For in-gel detection of telomere length a total of 1–2 × 10^6^ cells were suspended in PBS, mixed 1:1 with 2% agarose in 1× PBS and cast into plugs. The plugs were digested overnight at 50 °C with 1 mg/ml Proteinase K (Roche) in 10 mM sodium phosphate (pH 7.2), 0.5 mM EDTA and 1% sodium lauryl sarcosine. The DNA in the plugs was then digested with *Hinf*I/*Rsa*I at 37 °C overnight. The next morning plugs were washed once in 1 × TE and equilibrated with 0.5 × TBE before loading them onto a 1% agarose gel in 0.5 × TBE. The gel was run at 85 V for 4 h and dried. The gel was denatured in 0.6 M NaCl/0.2 M NaOH for 1 h at room temperature, neutralized in 1.5 M NaCl/0.5 M Tris pH 7.4 for 1 h at room temperature and washed with water for 30 min. The dried gel was pre-hybridized with Church mix for 2 h at 55 °C and hybridized overnight at 55 °C in Church mix with a ^32^P-labeled (CCCTAA)_4_ probe. After hybridization, the gel was washed three times for 30 min with 4 × SSC/0.1% SDS at 37 °C, thrice with 4 × SSC/0.1% SDS at 55 °C and exposed to a phosphoimager screen overnight before scanning with a Typhoon Trio imager system. The gel was subsequently denatured and hybridized again using a ^32^P-labeled Alu specific probe.

### SMARD assay

The telomere SMARD assay was performed as described by Sfeir et al.^52^ with minor changes. U2OS cells transfected with either Scrambled or *MCPH1* shRNA were sequentially labeled with 25 μM CldU and 25 μM IdU, 1 h each, with three PBS washes in between followed by incubation in fresh media without CldU/IdU for 2 h. The labeling process was repeated three times before harvesting the cells. To analyze replication fork restart, transfected U2OS cells were labeled with 25 μM CldU for 1 h, washed three times with PBS and then treated for 2 h with 2 mM HU in fresh media to block replication. The block was released by removing the media and washing the cells three times with PBS, then cells were kept in fresh media containing 25 μM IdU for 2 h before harvesting. A total of 2 × 10^6^ cells were suspended in PBS, mixed 1:1 with 2% low melt agarose in 0.5 × TBE and cast into plugs. The plugs were then digested overnight at 50 °C with 1 mg/ml Proteinase K (Roche) in 10 mM sodium phosphate (pH 7.2), 0.5 mM EDTA and 1% sodium lauryl sarcosine. DNA in plugs was subsequently digested by *Hinf*I/*Rsa*I overnight at 37 °C. The next morning, plugs were washed once with 1 × TE and equilibrated with 0.5 × TBE. The plugs were loaded onto a 1% low melt agarose gel in 0.5 × TBE and run for 3 h at 35 V at room temperature. After the run, the gel was stained with ethidium bromide to detect and isolate the high molecular weight band corresponding to telomere fragments. The gel slices containing telomeric fragments were melted in agarose digestion buffer (TE pH 8, 100 mM NaCl, 0.1% β-mercaptoethanol) for 20 min at 68 °C and digested with β-agarase I at 45 °C for 4 h. DNA fibers were stretched on microscope slides coated with (3-aminopropyl)triethoxysilane (Alfa Aesar), denatured with alkali-denaturing buffer (0.1 N NaOH in 70% ethanol and 0.1% β-mercaptoethanol) for 12 min and fixed by adding 0.5% glutaraldehyde for 5 minutes. Telomeric DNA was detected through hybridization with a 5′-Biotin-OO-(CCCTAA)_4_-3′ PNA probe (PNA Bio), followed by three sequential incubation with Streptavidin AlexaFluor 405 (Invitrogen #S32351, 1:250 dilution) and anti-streptavidin antibody (Vector Laboratories #BA-0500, 1:50 dilution). Halogenated nucleotides were detected using Rat anti-BrdU (Abcam #ab6326, 1:50 dilution) for CldU and Mouse anti-BrdU (BD Biosciences #347580, 1:50 dilution) for IdU, followed by incubation with Cy3.5-conjugated Goat anti-Mouse (Abcam #ab6946, 1:250 dilution) and Cy5-conjugated Goat anti-Rat (Abcam #ab6565, 1:250 dilution). Digital images for the SMARD assay were captured using the software NIS-Elements BR (Nikon) with a Nikon Eclipse 80i microscope and an Andor CCD camera. Fibers length was analyzed using the ImageJ software. Measured length values are included in Source data file.

### Clonogenic survival assay

Cells were seeded in 96-well plates (200 cells/plate) and incubated with regular media (untreated) or with various concentrations of either HU or Olaparib in triplicate for 120 h. Cells were stained in Crystal Violet staining solution (0.5% Crystal Violet in 20% methanol) for 20 min, washed four times with distilled water and air dried at room temperature overnight. After incubation with methanol for 20 min, the optical density at 570 nm (OD_570_) of each well was measured with a plate reader. The fraction of surviving cells for each concentration of either HU or Olaparib was determined comparing the average OD_570_ of treated cells with the average OD_570_ of untreated cells. Normalized OD values are shown in Source data file.

### Reporting summary

Further information on research design is available in the Nature Research Reporting Summary linked to this article.

## Supplementary information

Supplementary Information

Reporting Summary

## Data Availability

Coordinate and structure factor have been deposited in the Protein Data Bank under accession code 7C5D. The authors declare that all other data supporting the findings of this study are available within the paper and its Supplementary information files. Source data are provided with this paper.

## Source data

Source Data

## Materials availability

Further information and requests for resources and reagents should be directed to and will be fulfilled by Dr. Sandy Chang (s.chang@yale.edu). All plasmids and cell lines generated in this study are available on request without restrictions.
