# Supplementary material for: Microcephalin 1/BRIT1-TRF2 interaction promotes telomere replication and repair, linking telomere dysfunction to primary microcephaly
Source: Nat Commun. 2020 Nov 17;11:5861. doi: 10.1038/s41467-020-19674-0 (PMC7672075; doi:10.1038/s41467-020-19674-0)
Supplement: Supplementary file 1 — Supplementary Information [file 41467_2020_19674_MOESM1_ESM.pdf]

Supplementary Figure 1

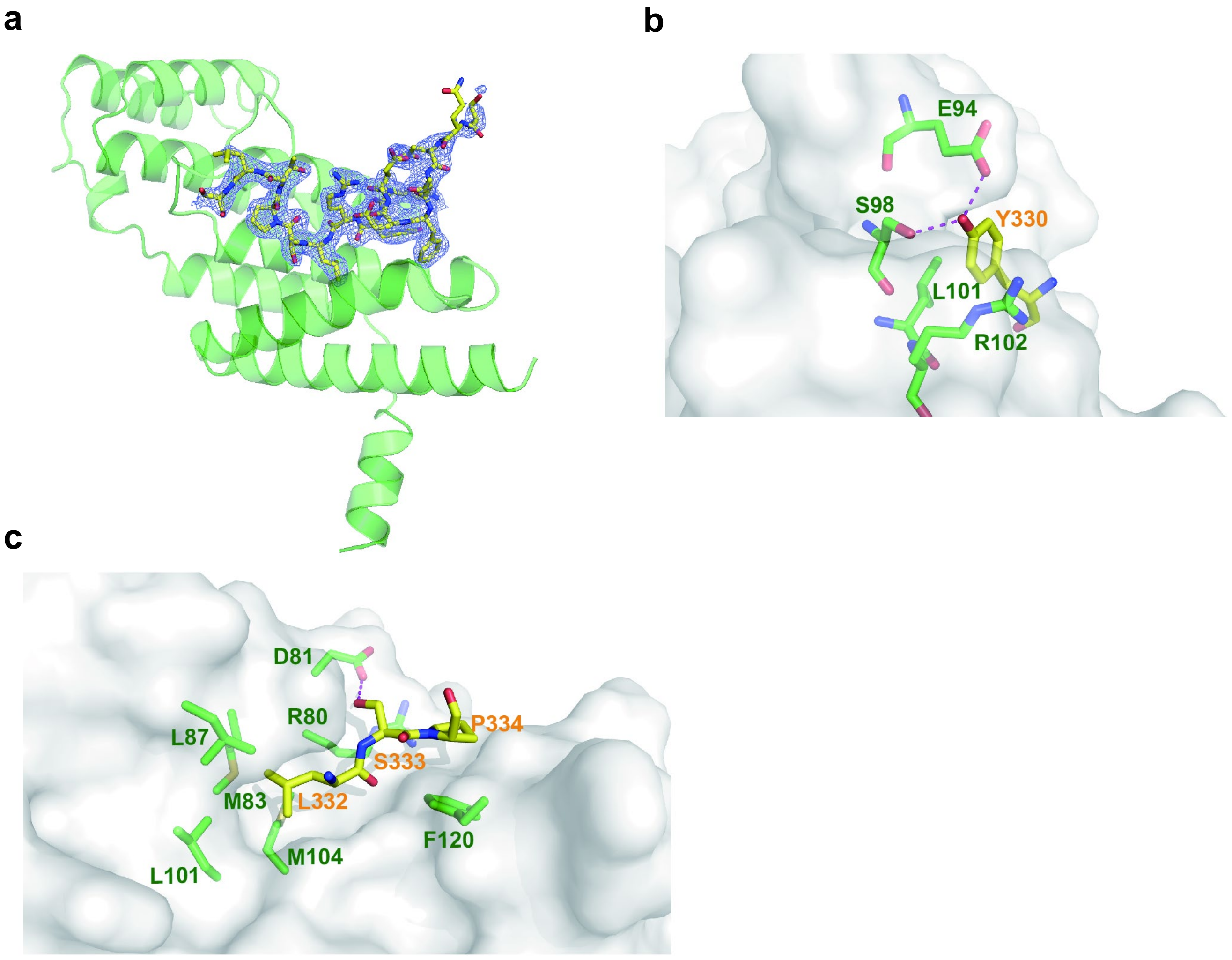

Supplementary Figure 1: Analysis of the MCPH1^{TBM}-TRF2^{TRFH} crystal structure. (a) Electron density map (2Fo-Fc) of BRIT1^{TBM} (yellow) in complex with TRF2^{TRFH} (green). **(b-c)** Detailed molecular interactions involved in MCPH1^{TBM} (yellow) and TRF2^{TRFH} (green) binding. The panels show the interactions that occur between MCPH1^{TBM} amino acid residues Y330 (**b**) and $_{332}$LSP$_{334}$ (**c**) and TRF2^{TRFH}. Hydrogen bonding: magenta dashed lines.

Supplementary Figure 2

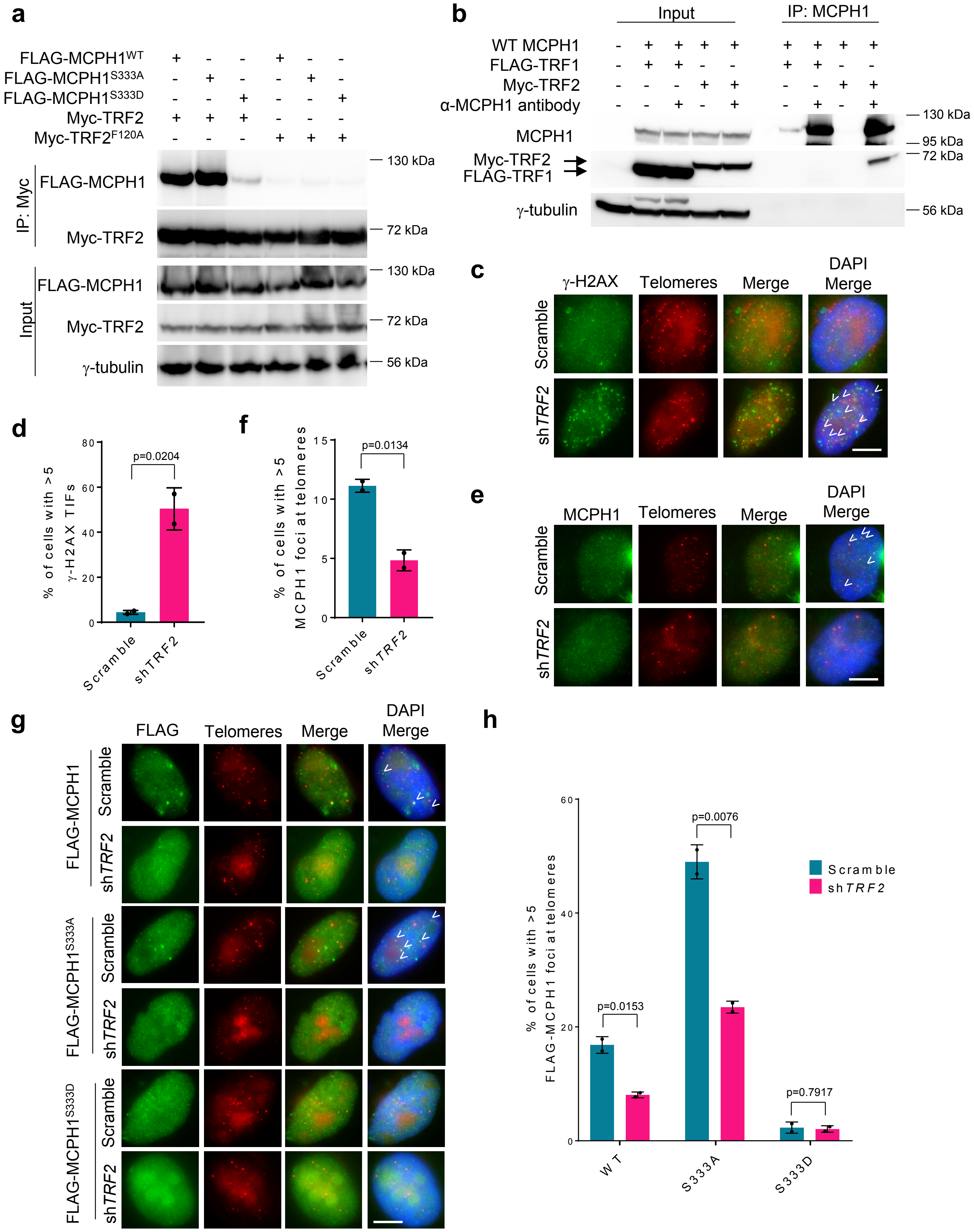

i

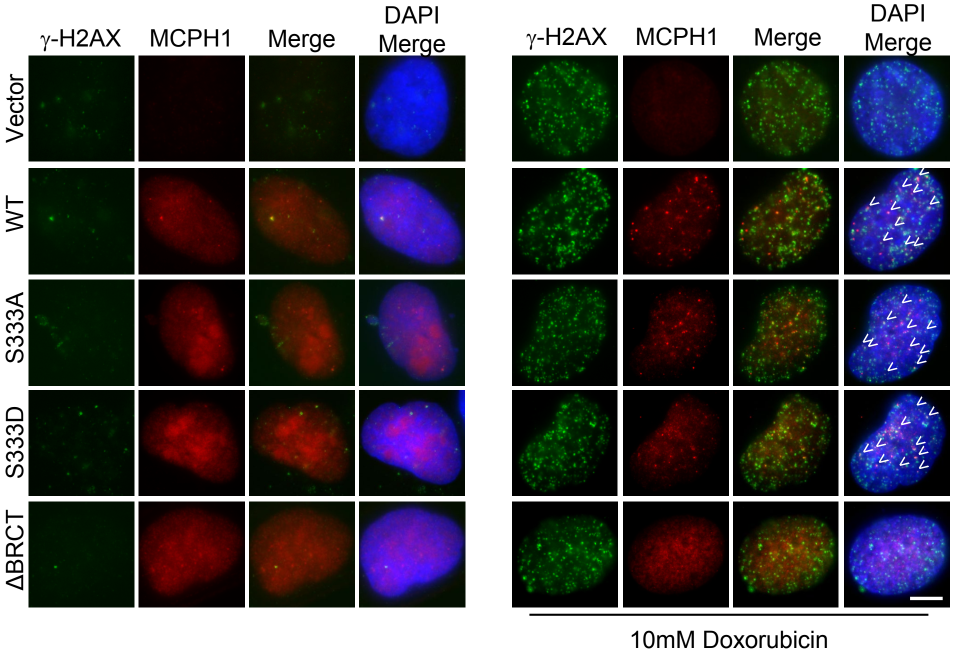
γ-H2AX
MCPH1
Merge
DAPI Merge
Vector
WT
S333A
S333D
ΔBRCT
10mM Doxorubicin

j

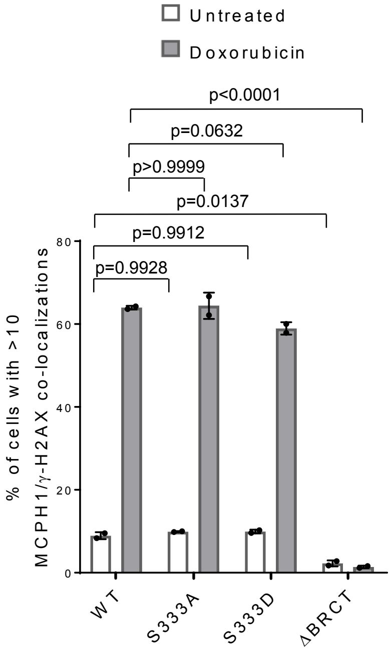
Untreated
Doxorubicin
p<0.0001
p=0.0632
p>0.9999
p=0.0137
p=0.9912
p=0.9928
% of cells with >10 MCPH1/γ-H2AX co-localizations
0
20
40
60
80
WT
S333A
S333D
ΔBRCT

Supplementary Figure 2: MCPH1 localization at telomeres is TRF2-dependent while its localization at genomic DSBs is not. (**a**) Co-immunoprecipitation with anti-Myc antibody-conjugated agarose beads from lysates of 293T cells expressing either Myc-TRF2 or Myc-TRF2^{F120A} and either FLAG-WT MCPH1 or FLAG-MCPH1^{TBM} mutants. Representative blots from two independent experiments. (**b**) Co-immunoprecipitation with an anti-MCPH1 antibody followed by incubation with Protein A/Protein G agarose beads from lysates of 293T cells expressing either FLAG-TRF1 or Myc-TRF2 and a non-tagged WT MCPH1 construct. Samples incubated only with the beads (no antibody) were used as a negative control. Representative blot from two independent experiments. (**c**) TIF assay to confirm TRF2 depletion through analysis of γ-H2AX TIFs in HeLa cells treated with either Scramble or *TRF2* shRNA. An anti-γ-H2AX antibody (green) and a telomeric DNA probe (red) were used. Representative images from two independent experiments. Scale bar: 5 μm. (**d**) Quantification of the percentage of cells showing >5 γ-H2AX TIFs from **c**. Data show the mean ± standard deviation (SD) from two independent experiments. At least 200 nuclei were analyzed for each sample. Two-sided Student's t test. (**e**) Immunofluorescence-FISH to visualize the localization of endogenous MCPH1 at telomeres in HeLa cells treated with either Scramble or *TRF2* shRNA, using a MCPH1 specific antibody (green) and a telomeric PNA probe (red). Representative images from two independent experiments. Scale bar: 5 μm. (**f**) Quantification of the percentage of cells with >5 MCPH1 foci at telomeres from **e**. Data represent the mean of two independent experiments ± SD. At least 200 nuclei were scored for each sample. Two-sided Student's t test. (**g**) IF-FISH to analyze MCPH1 telomeric localization in HeLa cells overexpressing the indicated FLAG-tagged MCPH1 constructs with or without concomitant TRF2 depletion. A FLAG antibody (green) was used to detect FLAG-MCPH1, while telomeres were detected with a PNA probe (red). Representative images from two independent experiments. Scale bar: 5 μm. (**h**) Quantification of the percentage of cells showing >5 MCPH1 foci at telomeres in **g**. Data show the mean ± SD from two independent experiments. At least 200 nuclei were analyzed for each sample. One-way analysis of variance (ANOVA) followed by Tukey's multiple

comparison test. (**i**) Immunostaining for γ-H2AX and MCPH1 co-localization in U2OS cells expressing either empty vector, WT MCPH1, $MCPH1^{S333A}$, $MCPH1^{S333D}$ or $MCPH1^{\Delta BRCT}$. Antibodies were used to detect γ-H2AX (green) and MCPH1 (red), nuclei were stained with DAPI (blue). Treatment with 10 mM doxorubicin for 2 hours was used to induce genomic DSBs. Representative images from two independent experiments. Scale bar: 5 µm. (**j**) Quantification of the percentage of cells showing >10 γ-H2AX/MCPH1 co-localizations from **i**. Data represent the mean of two independent experiments ± standard deviation. A minimum of 200 nuclei per sample were examined in each experiment. One-way ANOVA followed by Tukey's multiple comparison test.

Supplementary Figure 3

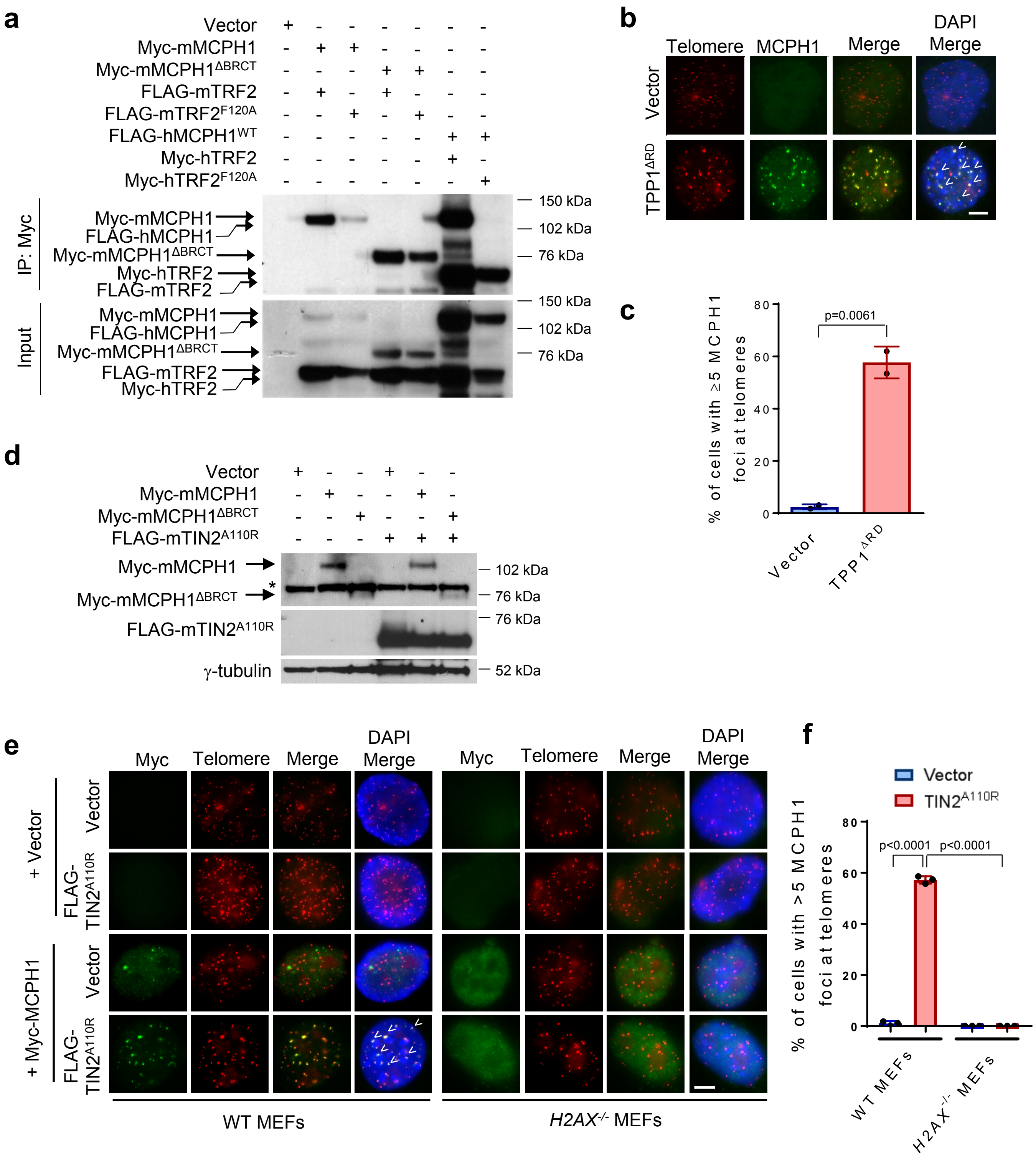

Supplementary Figure 3: Murine MCPH1 localizes to dysfunctional telomeres through interaction with γ-H2AX. (**a**) Co-immunoprecipitation performed on lysates of 293T cells expressing FLAG-mTRF2 and either Myc-mMCPH1 or Myc-mMCPH1$^{\Delta BRCT}$. Myc-mMCPH1 was immunoprecipitated using α-Myc coated beads and the presence of FLAG-mTRF2 was detected by Western Blot. TRF2^{F120A} was used as a negative control, since it is not able to interact with proteins containing a TBM. Human Myc-TRF2 and FLAG-MCPH1 were used as a positive control of the Co-IP. Anti-FLAG and anti-Myc antibodies were used with subsequent incubations without stripping the blot, allowing detection of all the proteins at the same time. Representative blots from two independent experiments. (**b**) Immunostaining to detect endogenous MCPH1 in WT MEFs expressing either empty vector or TPP1$^{\Delta RD}$. MCPH1 was detected using an anti-MCPH1 antibody (green), telomeres were labeled with a Cy3-OO-(CCCTAA)$_4$ PNA probe (red) and nuclei were stained with DAPI (blue). Representative images from two independent experiments. Scale bar: 5 μm. (**c**) Quantification of the percentage of MEFs displaying at least 5 MCPH1 foci at telomeres from **b**. Data represent the mean of two independent experiments ± SD. At least 200 cells were scored for each sample. Two-sided Student's t test. (**d**) Immunoblot to detect the expression of the constructs Myc-WT mMCPH1 and Myc-mMCPH1$^{\Delta BRCT}$, with or without concomitant expression of FLAG-mTIN2^{A110R}, in WT MEFs. The asterisk indicates a non-specific band detected by the α-Myc antibody. Representative blot from two independent experiments. (**e**) Immunostaining to detect MCPH1 telomeric localization in both WT and *H2AX*$^{-/-}$ MEFs expressing either empty vector or FLAG-mTIN2^{A110R} in combination with either empty vector or Myc-mMCPH1. Myc-mMCPH1 constructs were detected with an anti-Myc specific antibody (green), telomeres were visualized with PNA-FISH (red), nuclei were stained with DAPI (blue). Representative images from two independent experiments. Scale bar: 5 μm. (**f**) Quantification of the percentage of cells with >5 MCPH1 foci at telomeres observed in **e**. Data represent the mean of two independent experiments ± SD. For each sample, at least 200 cells were scored per experiment. One-way ANOVA followed by Tukey's multiple comparison test.

Supplementary Figure 4

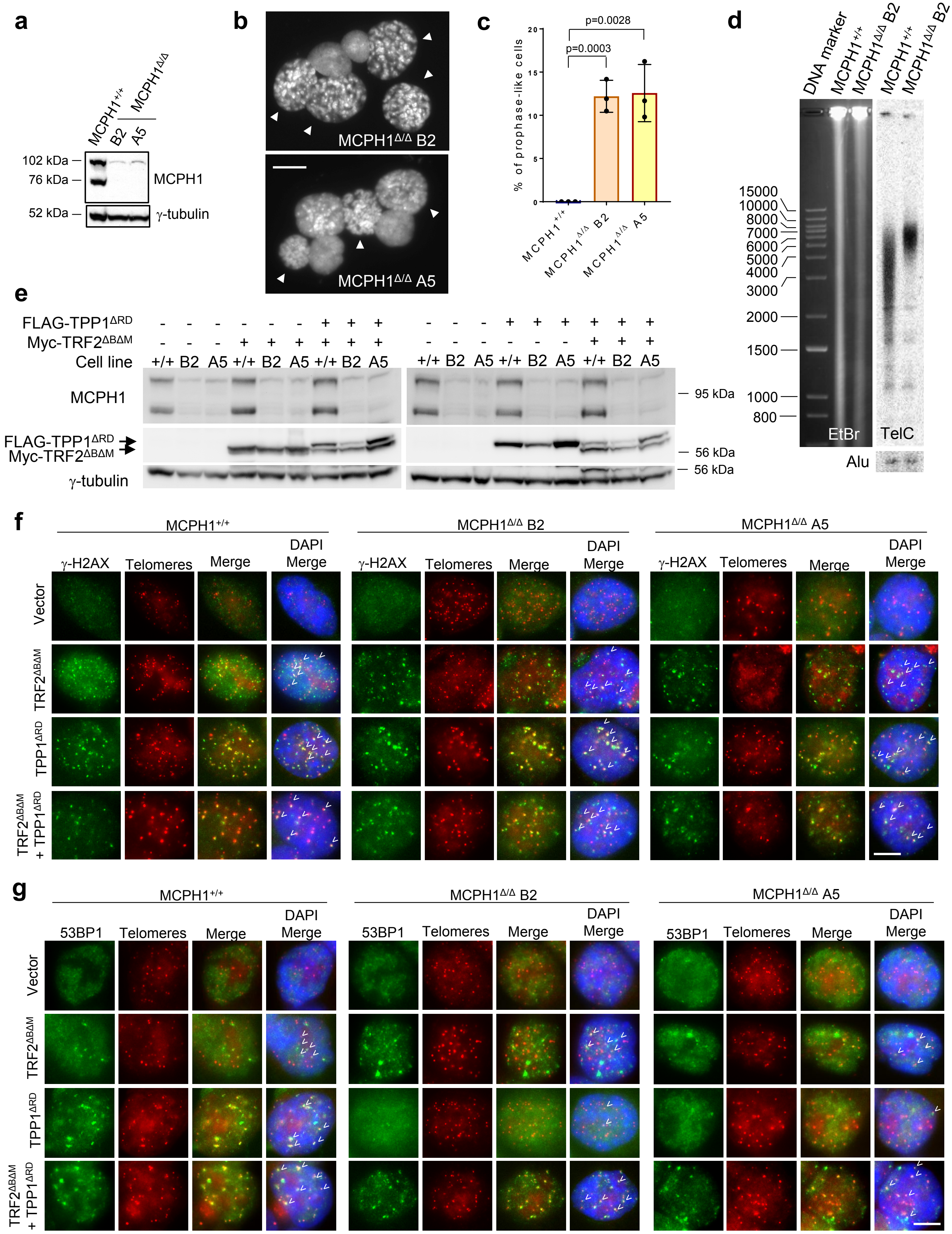

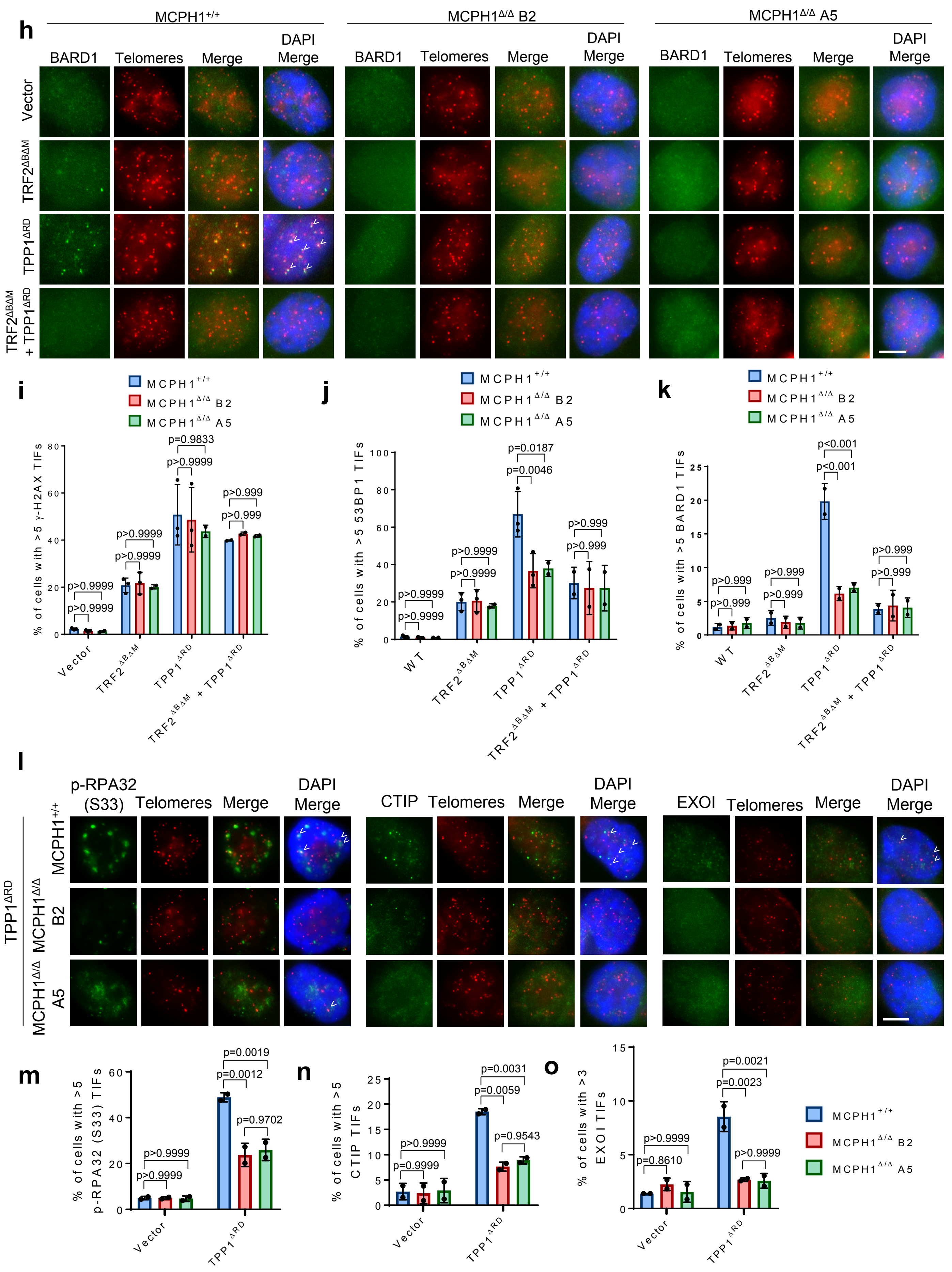
h
MCPH1+/+
MCPH1Δ/Δ B2
MCPH1Δ/Δ A5
BARD1
Telomeres
Merge
DAPI Merge
Vector
TRF2ΔBΔM
TPP1ΔRD
TRF2ΔBΔM + TPP1ΔRD
i
j
k
MCPH1+/+
MCPH1Δ/Δ B2
MCPH1Δ/Δ A5
% of cells with >5 γ-H2AX TIFs
% of cells with >5 53BP1 TIFs
% of cells with >5 BARD1 TIFs
Vector
WT
TRF2ΔBΔM
TPP1ΔRD
TRF2ΔBΔM + TPP1ΔRD
p>0.9999
p=0.9833
p>0.999
p=0.0187
p=0.0046
p<0.001
l
p-RPA32 (S33)
CTIP
EXOI
TPP1ΔRD
MCPH1+/+
MCPH1Δ/Δ B2
MCPH1Δ/Δ A5
m
n
o
% of cells with >5 p-RPA32 (S33) TIFs
% of cells with >5 CTIP TIFs
% of cells with >3 EXOI TIFs
p=0.0019
p=0.0012
p=0.9702
p=0.0031
p=0.0059
p=0.9543
p=0.0021
p=0.0023
p=0.8610
Vector
TPP1ΔRD

Supplementary Figure 4: Two CRISPR/Cas9 generated MCPH1$^{\Delta/\Delta}$ cell lines show defects in the DDR at dysfunctional telomeres lacking POT1-TPP1. (**a**) Immunoblot analysis of MCPH1 expression in HCT116 cells not infected with MCPH1 sgRNA and in two CRISPR/Cas9 MCPH1$^{\Delta/\Delta}$ clones (named B2 and A5). γ-tubulin was used as a loading control. This Western blot was performed once to validate the CRISPR/Cas9 generated cell lines, but loss of MCPH1 expression in these cell lines was confirmed multiple times in subsequent experiments. (**b**) Representative images of prophase-like nuclei (arrowheads) observed in the two MCPH1$^{\Delta/\Delta}$ cell lines in three independent experiments. The nuclei were visualized through DAPI staining. Scale bar: 10 µm. (**c**) Quantification of the percentage of prophase-like nuclei on total cells observed in **b**. Data represent the mean of three independent experiments ± SD. A minimum of 200 cells was examined in each experiment. Two-sided Student's t test. (**d**) Telomere restriction fragment (TRF) Southern blot to analyze telomere length in MCPH1$^{+/+}$ and MCPH1$^{\Delta/\Delta}$ HCT116 (clone B2). The samples were run in an agarose gel (EtBr) followed by in-gel hybridization with a radiolabeled $(CCCTAA)_4$ probe (TelC) to detect the total telomeric repeats. Re-hybridization with an Alu repetitive DNA probe was used to quantify the total DNA amount. (**e**) Immunoblot analysis of protein expression in MCPH1$^{+/+}$ cells and the two MCPH1$^{\Delta/\Delta}$ clones (B2, A5) expressing either empty vector, Myc-TRF2$^{\Delta B\Delta M}$, FLAG-TPP1$^{\Delta RD}$ or both Myc-TRF2$^{\Delta B\Delta M}$ and FLAG-TPP1$^{\Delta RD}$. Representative blots from two independent experiments. See also Figure 3a-b. (**f-h**) TIF assay to detect γ-H2AX (**f**), 53BP1 (**g**) and BARD1 (**h**) foci at telomeres in MCPH1$^{+/+}$ and the two MCPH1$^{\Delta/\Delta}$ clones expressing either empty vector, Myc-TRF2$^{\Delta B\Delta M}$, FLAG-TPP1$^{\Delta RD}$ or both Myc-TRF2$^{\Delta B\Delta M}$ and FLAG-TPP1$^{\Delta RD}$. Antibodies were used to detect γ-H2AX, 53BP1, and BARD1 foci (green) co-localizing with the signal of a Cy3-OO-$(CCCTAA)_4$ PNA probe (red). DAPI was used to stain the nuclei (blue). Representative images from either three (panels **f** and **g**: MCPH1$^{+/+}$ and MCPH1$^{\Delta/\Delta}$ B2 cells + either vector, TRF2$^{\Delta B\Delta M}$ or TPP1$^{\Delta RD}$) or two (panels **f** and **g**: MCPH1$^{\Delta/\Delta}$ A5 cells and samples with TRF2$^{\Delta B\Delta M}$ + TPP1$^{\Delta RD}$; panel **h**) independent experiments. Scale bars: 5 µm. (**i-k**) Quantification of the percentage of nuclei with >5 γ-H2AX (**i**), 53BP1 (**j**) and BARD1 (**k**)

TIFs observed in **f-h**. Data represent the mean values ± SD from either n=3 (panels **i** and **j**: MCPH1$^{+/+}$ and MCPH1$^{\Delta/\Delta}$ B2 cells + either vector, TRF2$^{\Delta B\Delta M}$ or TPP1$^{\Delta RD}$) or n=2 (panels **i** and **j**: MCPH1$^{\Delta/\Delta}$ A5 cells and samples with TRF2$^{\Delta B\Delta M}$ + TPP1$^{\Delta RD}$; panel **k**) independent experiments. A minimum of 200 cells were scored in each experiment. One-way ANOVA followed by Tukey's multiple comparison test. (**l**) TIF assay to detect p-RPA32 (S33), CTIP and EXOI foci at telomeres in MCPH1$^{+/+}$ and the two MCPH1$^{\Delta/\Delta}$ clones expressing either empty vector or FLAG-TPP1$^{\Delta RD}$. Antibodies were used to detect p-RPA32 (S33), CTIP and EXOI foci (green), telomeres were detected with a telomeric PNA probe probe (red) and DAPI was used to stain the nuclei (blue). RPA32 was overexpressed to detect TIFs. Representative images from two independent experiments. Scale bar: 5 µm. (**m-o**) Quantification of the percentage of nuclei with >5 p-RPA32 (S33) (**m**) and CTIP (**n**) TIFs and with >3 EXOI (**o**) TIFs observed in **l**. Data representing the mean ± SD from two independent experiments. A minimum of 200 cells were scored in each experiment. One-way ANOVA followed by Tukey's multiple comparison test.

Supplementary Figure 5

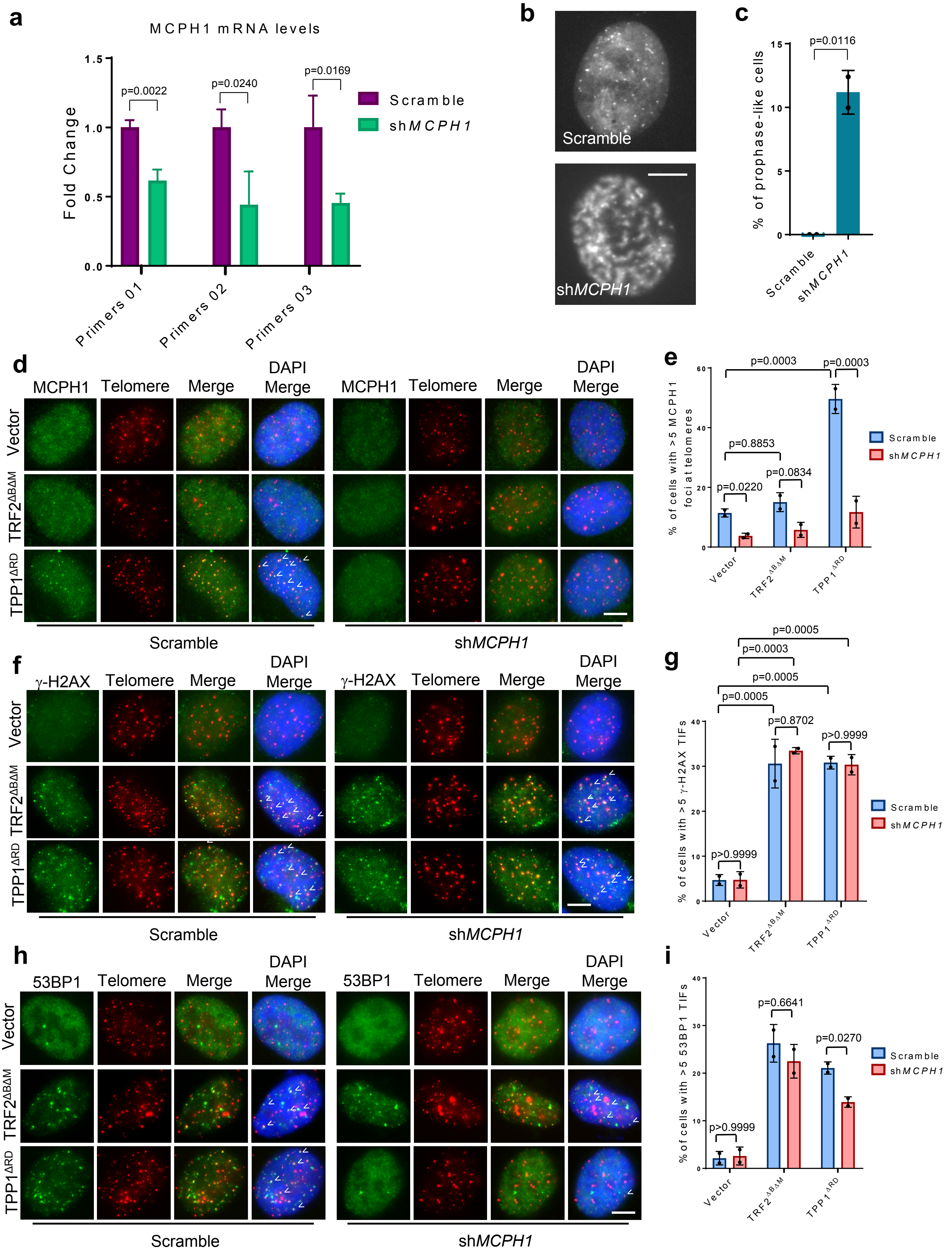

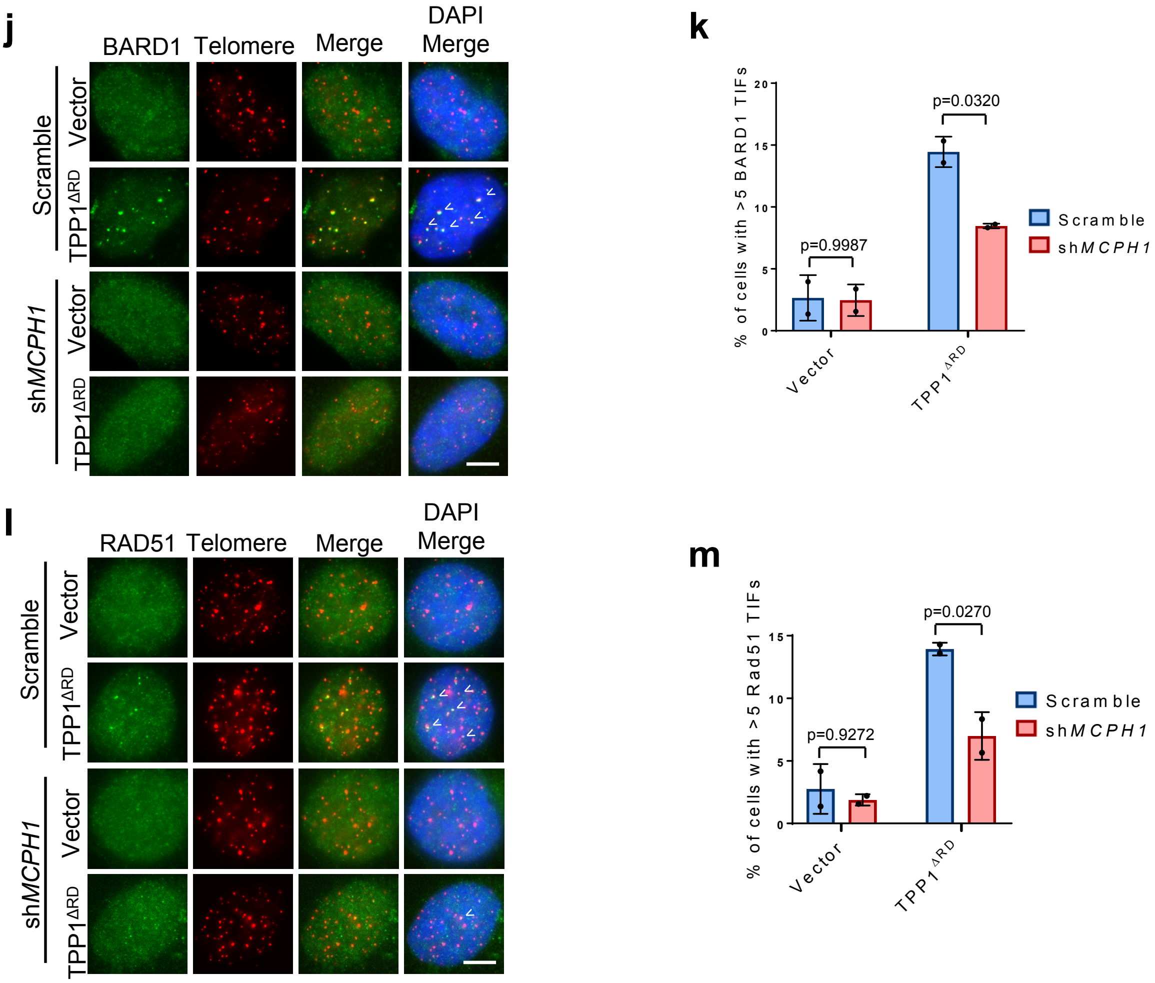
j
BARD1
Telomere
Merge
DAPI Merge
Scramble
Vector
TPP1ΔRD
shMCPH1
Vector
TPP1ΔRD
k
% of cells with >5 BARD1 TIFs
0
5
10
15
20
p=0.9987
p=0.0320
Vector
TPP1ΔRD
Scramble
shMCPH1
l
RAD51
Telomere
Merge
DAPI Merge
Scramble
Vector
TPP1ΔRD
shMCPH1
Vector
TPP1ΔRD
m
% of cells with >5 Rad51 TIFs
0
5
10
15
p=0.9272
p=0.0270
Vector
TPP1ΔRD
Scramble
shMCPH1

Supplementary Figure 5: MCPH1 localizes at telomeres lacking POT1-TPP1 and promotes recruitment of downstream DNA damage signaling and DNA repair factors in U2OS cells. (**a**) RT-qPCR analysis to assess MCPH1 expression in U2OS treated with either scrambled or *MCPH1* shRNA. The amplification was performed with three different sets of primers. Data shows the fold change ± standard error in MCPH1 mRNA levels calculated from the Ct values obtained in triplicate samples using the $2^{-\Delta\Delta Ct}$ method. Actin levels were used for normalizations. Data from one of two independent experiments. (**b**) Representative images of prophase-like nuclei in MCPH1-depleted U2OS cells from two independent experiments. Nuclei were visualized through DAPI staining. Scale bar: 5µm. (**c**) Quantification of the percentage of prophase-like nuclei observed in **b**. Data represent the mean of two independent experiments ± SD. At least 200 nuclei were scored in each experiment. Two-sided Student's t test. (**d**) Immunostaining to address MCPH1 telomeric localization in U2OS cells expressing either empty vector, Myc-TRF2$^{\Delta B\Delta M}$ or FLAG-TPP1$^{\Delta RD}$ and either scrambled or *MCPH1* shRNA. Cells were stained with an anti-MCPH1 antibody to detect the endogenous protein (green), telomeres were visualized by PNA-FISH (red) and DAPI was used to stain the nuclei (blue). Representative images from two independent experiments. Scale bar: 5 µm. (**e**) Quantification of the percentage of cells with >5 MCPH1-positive foci at telomeres observed in **d**. Data represent the mean of two independent experiments ± SD. At least 200 nuclei were scored per experiment. One-way ANOVA followed by Tukey's multiple comparison test. (**f**) TIF assay to visualize γ-H2AX foci at telomeres in U2OS cells expressing either empty vector, Myc-TRF2$^{\Delta B\Delta M}$ or FLAG-TPP1$^{\Delta RD}$ and either scrambled or *MCPH1* shRNA. An antibody was used to detect γ-H2AX (green), telomeres were visualized by PNA-FISH (red) and DAPI was used to stain the nuclei (blue). Representative images from two independent experiments. Scale bar: 5 µm. (**g**) Quantification of the percentage of cells with >5 γ-H2AX TIFs observed in **f**. Data represent the mean of two independent experiments ± SD. At least 200 nuclei were scored per experiment. One-way ANOVA followed by Tukey's multiple comparison test. (**h**) TIF

assay to visualize 53BP1 foci at telomeres in U2OS cells expressing either empty vector, Myc-TRF2$^{\Delta B\Delta M}$ or FLAG-TPP1$^{\Delta RD}$ and either scrambled or *MCPH1* shRNA. An antibody was used to detect 53BP1 (green), telomeres were visualized by PNA-FISH (red) and DAPI was used to stain the nuclei (blue). Representative images from two independent experiments. Scale bar: 5 µm. (**i**) Quantification of the percentage of cells with >5 53BP1 TIFs observed in **h**. Data represent the mean of two independent experiments ± SD. At least 200 nuclei were scored per experiment. One-way ANOVA followed by Tukey's multiple comparison test. (**j**) TIF assay to visualize BARD1 foci at telomeres in U2OS cells expressing either empty vector or FLAG-TPP1$^{\Delta RD}$ and either scrambled or *MCPH1* shRNA. An antibody was used to detect BARD1 (green), telomeres were visualized by PNA-FISH (red) and DAPI was used to stain the nuclei (blue). Representative images from two independent experiments. Scale bar: 5 µm. (**k**) Quantification of the percentage of cells with >5 BARD1 TIFs observed in **j**. Data represent the mean of two independent experiments ± SD. At least 200 nuclei were scored per experiment. One-way ANOVA followed by Tukey's multiple comparison test. (**l**) TIF assay to visualize RAD51 foci at telomeres in U2OS cells expressing either empty vector or FLAG-TPP1$^{\Delta RD}$ and either scrambled or *MCPH1* shRNA. An antibody was used to detect RAD51 (green), telomeres were visualized by PNA-FISH (red) and DAPI was used to stain the nuclei (blue). Representative images from two independent experiments. Scale bar: 5 µm. (**m**) Quantification of the percentage of cells with >5 RAD51 TIFs observed in **l**. Data represent the mean of two independent experiments ± SD. At least 200 nuclei were scored per experiment. One-way ANOVA followed by Tukey's multiple comparison test.

Supplementary Figure 6

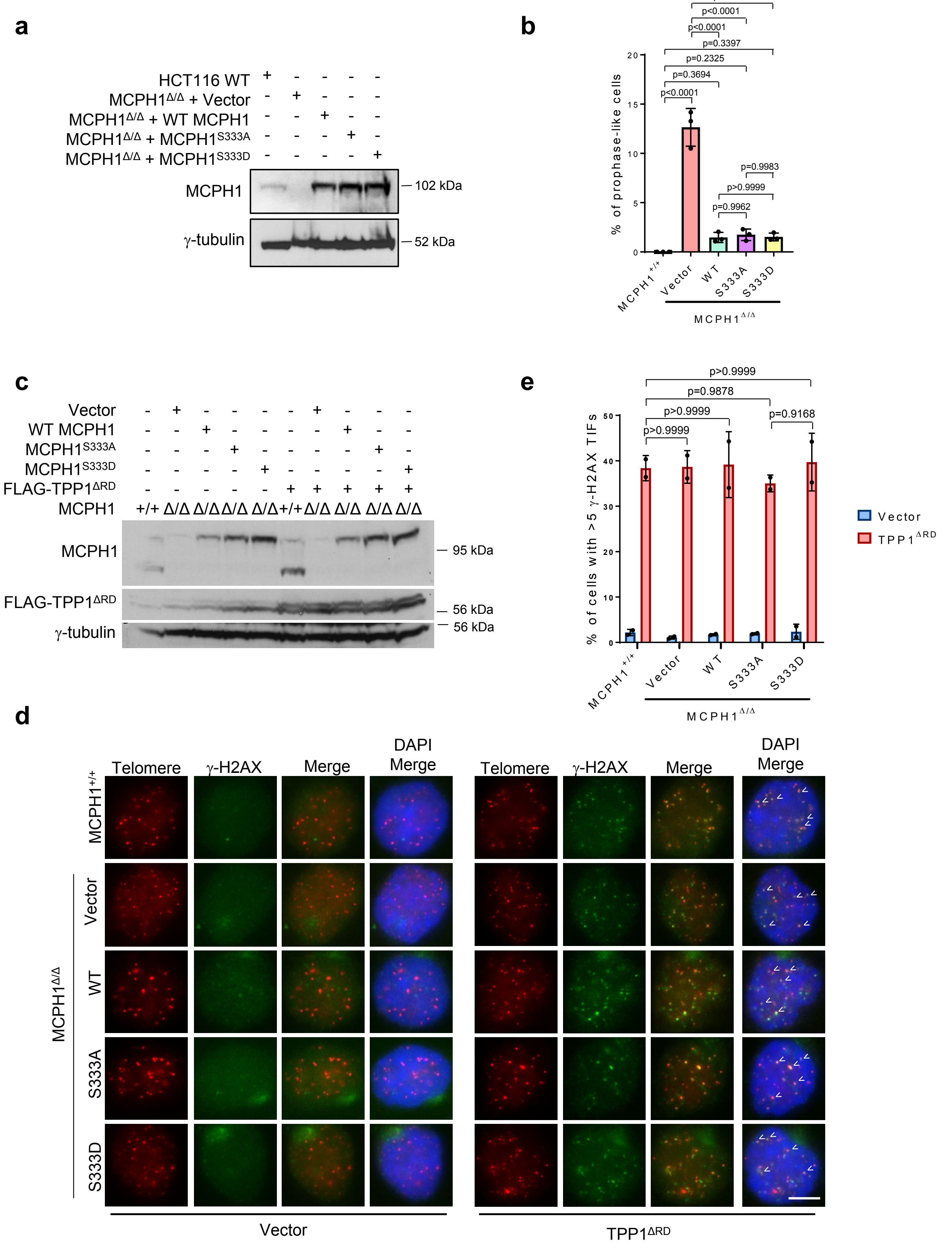

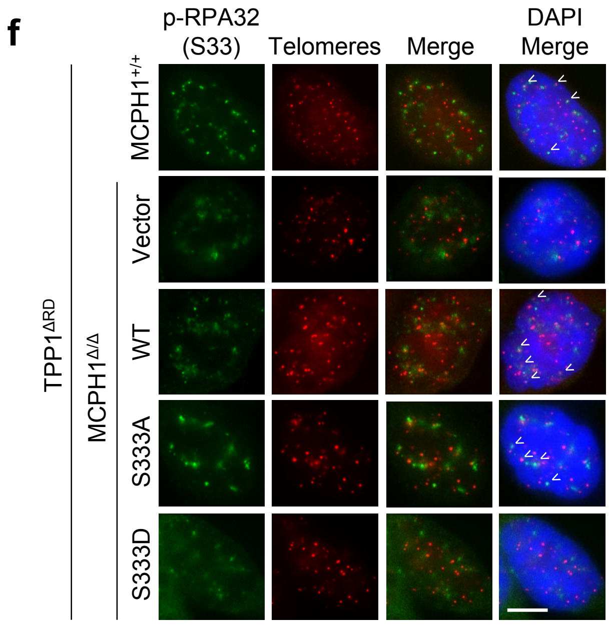
f
p-RPA32 (S33)
Telomeres
Merge
DAPI Merge
TPP1ΔRD
MCPH1+/+
MCPH1Δ/Δ
Vector
WT
S333A
S333D

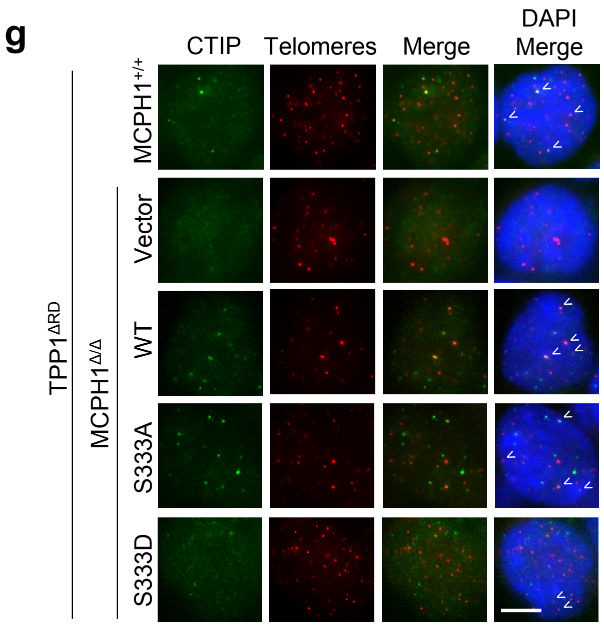
g
CTIP
Telomeres
Merge
DAPI Merge
TPP1ΔRD
MCPH1+/+
MCPH1Δ/Δ
Vector
WT
S333A
S333D

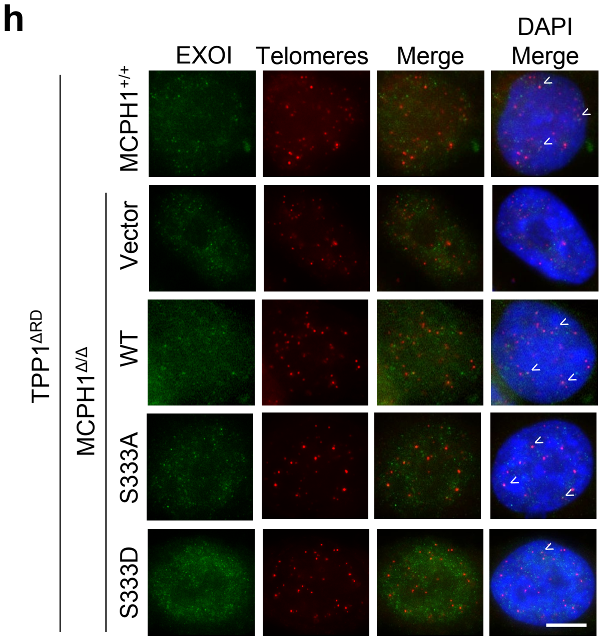
h
EXOI
Telomeres
Merge
DAPI Merge
TPP1ΔRD
MCPH1+/+
MCPH1Δ/Δ
Vector
WT
S333A
S333D

Supplementary Figure 6: Mutations in MCPH1^{S333} affect end resection at telomeres lacking POT1-TPP1. (**a**) Immunoblot for MCPH1 expression in WT and MCPH1$^{\Delta/\Delta}$ B2 HCT116 reconstituted with either empty vector, WT MCPH1, MCPH1^{S333A} or MCPH1^{S333D}. γ-tubulin was used as a loading control. Representative blot from three independent experiments. (**b**) Quantification of the percentage of prophase-like nuclei observed in MCPH1$^{+/+}$ cells and in MCPH1$^{\Delta/\Delta}$ cells after reconstitution with either empty vector, WT MCPH1, MCPH1^{S333A} or MCPH1^{S333D}. The data are representative of the mean ± SD from three independent experiments. A minimum of 100 nuclei were scored per sample. The statistical significance was determined using one-way ANOVA followed by Tukey's multiple comparison test. (**c**) Immunoblot analysis of protein expression in MCPH1$^{+/+}$ and MCPH1$^{\Delta/\Delta}$ B2 cells reconstituted with either empty vector, WT MCPH1, MCPH1^{S333A} or MCPH1^{S333D} and expressing either empty vector or FLAG-TPP1$^{\Delta RD}$. Representative blot from two independent experiments. See also Figure 3. (**d**) TIF assay to detect γ-H2AX foci at telomeres in MCPH1$^{+/+}$ and MCPH1$^{\Delta/\Delta}$ cells reconstituted with either empty vector, WT MCPH1, MCPH1^{S333A} or MCPH1^{S333D} and expressing either empty vector or FLAG-TPP1$^{\Delta RD}$. An antibody was used to detect γ-H2AX (green), telomeres were visualized by PNA-FISH (red) and cell nuclei were stained with DAPI (blue). Representative images from two independent experiments. Scale bar: 5 μm. (**e**) Quantification of the percentage of cells with >5 γ-H2AX TIFs as shown in **d**. Data are representative of the mean from two independent experiments ± SD. A minimum of 200 cells were examined per experiment. One-way ANOVA followed by Tukey's multiple comparison test. (**f-h**) Analysis of p-RPA32 (S33) (**f**), CTIP (**g**) and EXOI (**h**) TIFs in MCPH1$^{+/+}$ and MCPH1$^{\Delta/\Delta}$ cells reconstituted with either empty vector, WT MCPH1, MCPH1^{S333A} or MCPH1^{S333D} and expressing either empty vector or FLAG-TPP1$^{\Delta RD}$. An antibody was used to detect p-RPA32 (S33) (**f**), CTIP (**g**) and EXOI (**h**) (green), telomeres were visualized by PNA-FISH (red) and cell nuclei were stained with DAPI (blue). RPA32 was overexpressed to detect TIFs. The images are representative of two independent experiments. Scale bars: 5 μm. Quantifications are shown in Figs. 3e-g.

Supplementary Figure 7

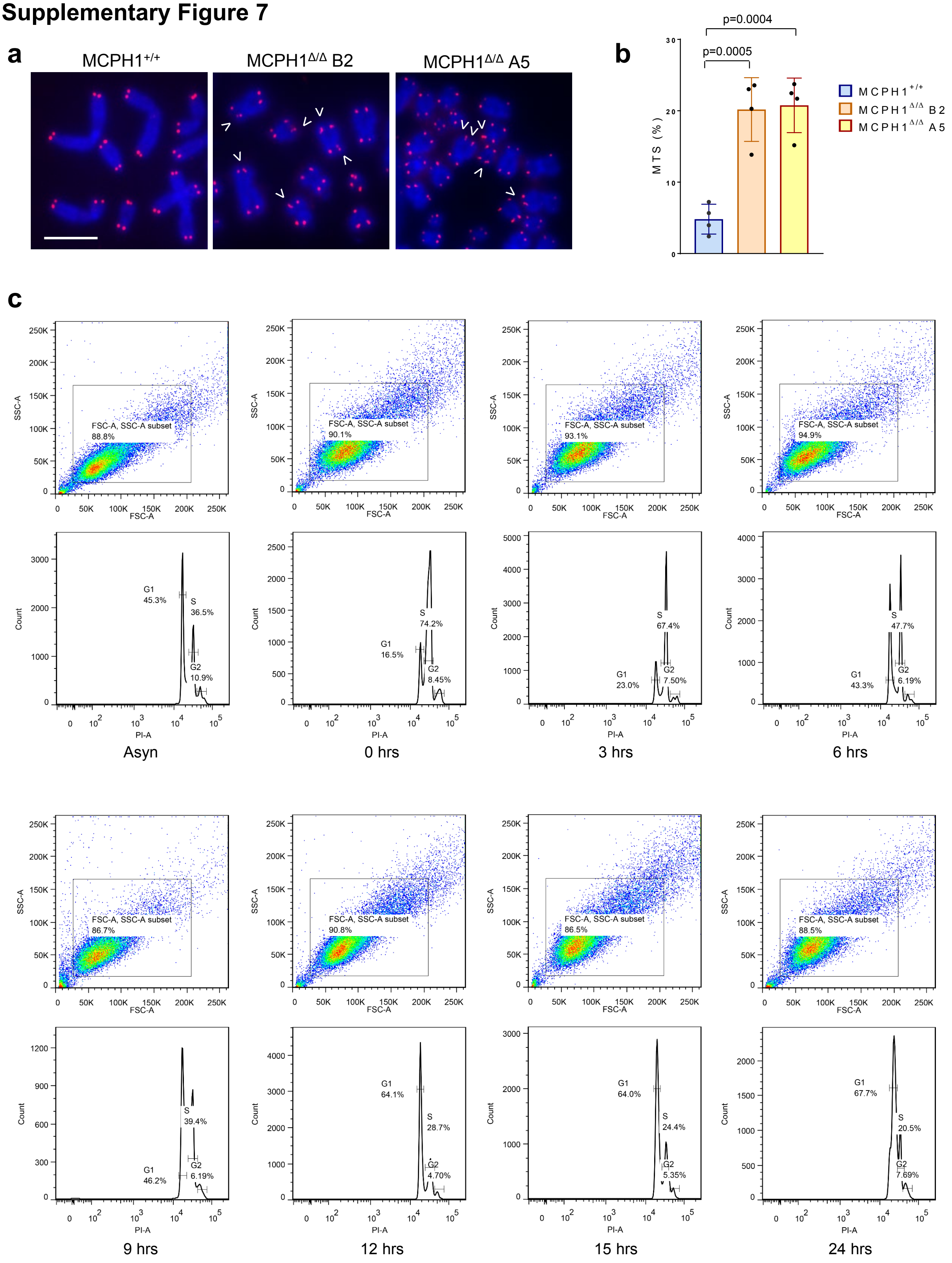

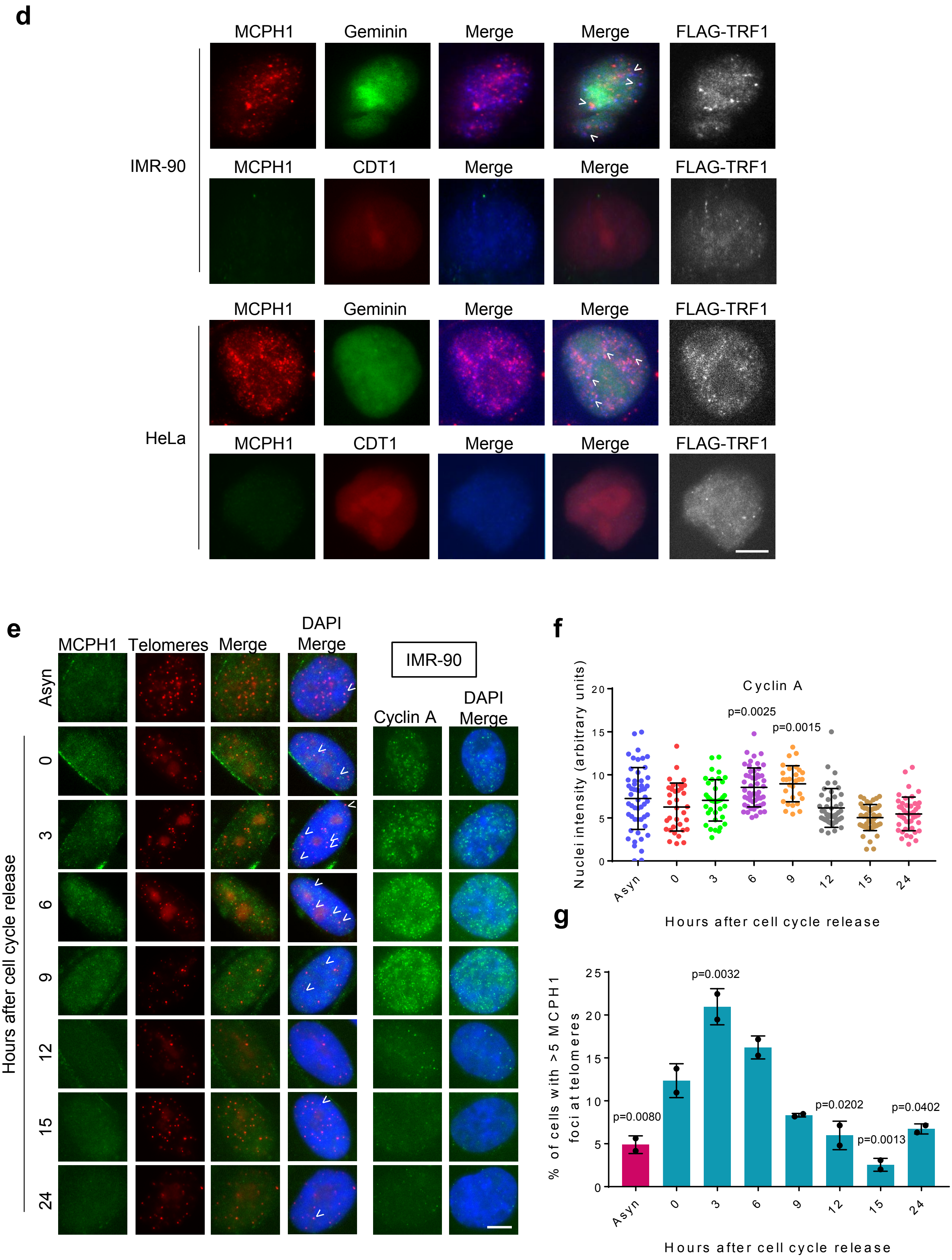

d
MCPH1
Geminin
Merge
Merge
FLAG-TRF1
IMR-90
MCPH1
CDT1
Merge
Merge
FLAG-TRF1
MCPH1
Geminin
Merge
Merge
FLAG-TRF1
HeLa
MCPH1
CDT1
Merge
Merge
FLAG-TRF1
e
MCPH1
Telomeres
Merge
DAPI Merge
IMR-90
Cyclin A
DAPI Merge
Asyn
Hours after cell cycle release
0
3
6
9
12
15
24
f
Cyclin A
Nuclei intensity (arbitrary units)
p=0.0025
p=0.0015
Asyn
0
3
6
9
12
15
24
Hours after cell cycle release
g
% of cells with >5 MCPH1 foci at telomeres
p=0.0080
p=0.0032
p=0.0202
p=0.0013
p=0.0402
Asyn
0
3
6
9
12
15
24
Hours after cell cycle release

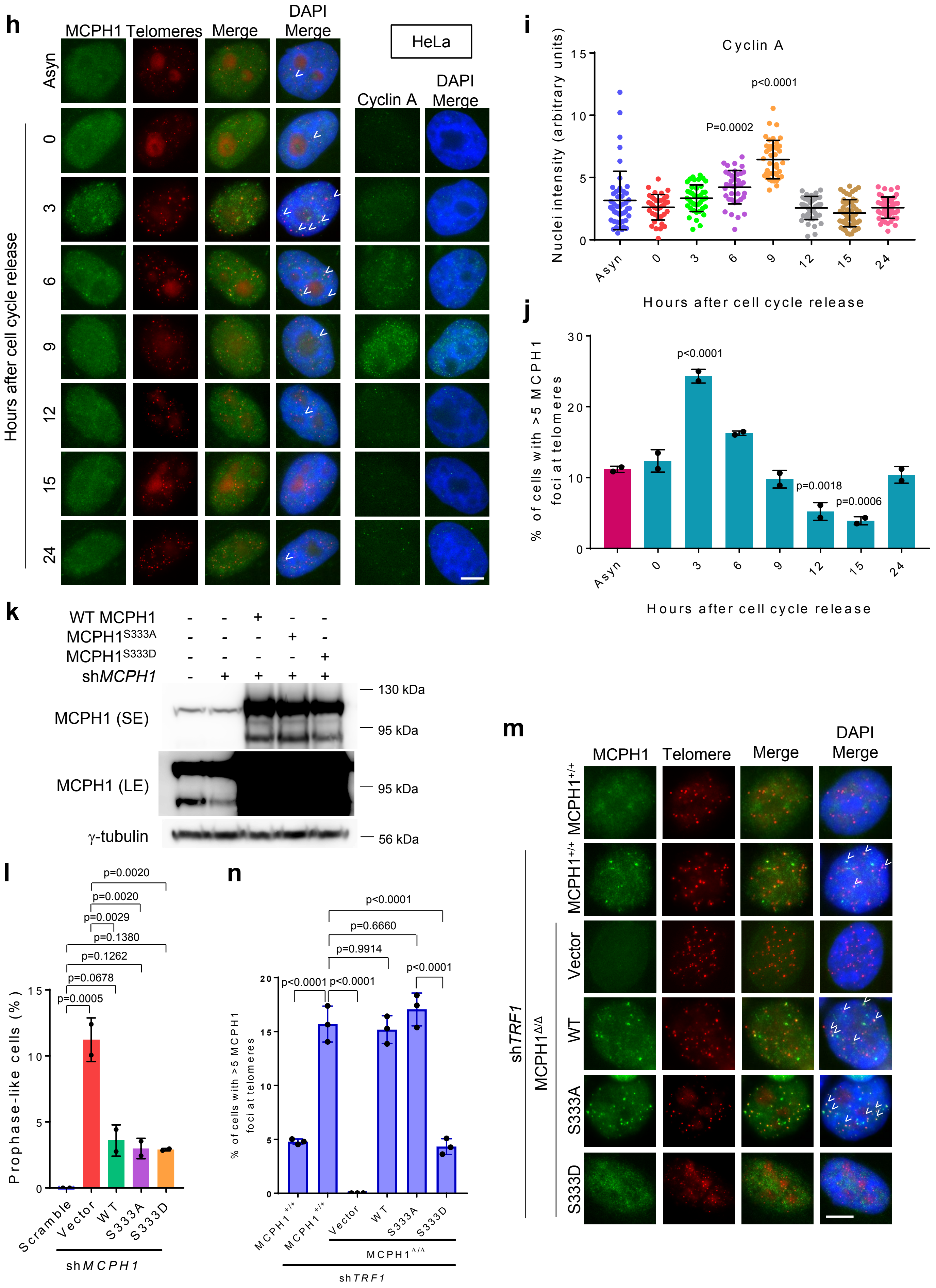
h
MCPH1
Telomeres
Merge
DAPI Merge
HeLa
Cyclin A
Asyn
Hours after cell cycle release
0
3
6
9
12
15
24
i
Cyclin A
Nuclei intensity (arbitrary units)
p<0.0001
P=0.0002
Hours after cell cycle release
j
% of cells with >5 MCPH1 foci at telomeres
p<0.0001
p=0.0018
p=0.0006
Hours after cell cycle release
k
WT MCPH1
MCPH1S333A
MCPH1S333D
shMCPH1
MCPH1 (SE)
MCPH1 (LE)
γ-tubulin
130 kDa
95 kDa
95 kDa
56 kDa
l
p=0.0020
p=0.0020
p=0.0029
p=0.1380
p=0.1262
p=0.0678
p=0.0005
Prophase-like cells (%)
Scramble
Vector
WT
S333A
S333D
shMCPH1
n
p<0.0001
p=0.6660
p=0.9914
p<0.0001
p<0.0001
p<0.0001
% of cells with >5 MCPH1 foci at telomeres
MCPH1+/+
MCPH1+/+
Vector
WT
S333A
S333D
MCPH1Δ/Δ
shTRF1
m
MCPH1
Telomere
Merge
DAPI Merge
MCPH1+/+
MCPH1+/+
Vector
WT
S333A
S333D
MCPH1Δ/Δ
shTRF1

Supplementary Figure 7. MCPH1 localizes at telomeres during S phase and in response to replication stress. (**a**) PNA-FISH to detect telomeres (red) on metaphase spreads of MCPH1$^{+/+}$, MCPH1$^{\Delta/\Delta}$ B2 and MCPH1$^{\Delta/\Delta}$ A5 HCT116. Arrowheads indicate MTS. The images are representative of four independent experiments. Scale bar: 5 µm. (**b**) Quantification of the percentage of multiple telomeric signals (MTS) on total chromosomes observed in **a**. Data represent the mean from four independent experiments ± SD. At least 40 metaphases were examined for each sample. One-way ANOVA followed by Tukey's multiple comparison test. (**c**) Cell-cycle analysis through Propidium Iodide flow cytometry of synchronized U2OS cells. Time points after release of the double thymidine block are indicated, with 0 hours corresponding to cells harvested before removing the block. For each time point the corresponding FSC-A vs. SSC-A gating plot is shown. (**d**) Cell-cycle analysis of MCPH1 localization in IMR-90 (top) and HeLa (bottom) cells expressing FLAG-TRF1. Expression of mKO1-hCDT1 (red) and mAG1-hGeminin (green) was used to distinguish G1 cells and S/G2 cells, respectively. Endogenous MCPH1 and FLAG-TRF1 were detected by immunostaining. Representative images from two independent experiments. Scale bar: 5 µm. Quantifications are shown in Figure 5d. (**e**) Immunostaining-PNA FISH analysis of MCPH1 telomeric localization in double thymidine synchronized IMR-90 cells using an anti-MCPH1 antibody (green) and a Cy3-OO-$(CCCTAA)_4$ PNA probe (red). Immunostaining for Cyclin A was used as a control of cell synchronization. The images are representative of two independent experiments. Scale bar: 5 µm. (**f**) Quantification of the intensity of Cyclin A staining in nuclei of synchronized IMR-90 from one of two independent experiments. The statistical analysis was performed using the Kruskal-Wallis test for non-parametric distributions followed by Dunn's multiple comparison test. P values are shown for those samples that displayed a statistically significant difference ($p<0.05$) with the 0-hour time point. (**g**) Quantification of the percentage of cells with >5 MCPH1 foci at telomeres observed in **e**. Data represent the mean of two independent experiments ± SD. At least 200 nuclei were scored for each

sample. Statistical significance was determined using one-way ANOVA with Tukey's multiple comparison test. P values are shown only for those samples that differ ($p<0.05$) from the 0-hour time point. (**h**) Immunostaining-PNA FISH analysis of MCPH1 telomeric localization in double thymidine synchronized HeLa cells. Cyclin A was used as a control of cell synchronization. Representative images from two independent experiments. Scale bar: 5 µm. (**i**) Quantification of the intensity of Cyclin A staining in HeLa from one of two independent experiments. The statistical analysis was performed using the Kruskal-Wallis test with Dunn's multiple comparison test. P values are shown for those samples that displayed a statistically significant difference ($p<0.05$) with the 0-hour time point. (**j**) Quantification of the percentage of cells with >5 MCPH1 foci at telomeres observed in **h**. Data represent the mean of two independent experiments ± SD. At least 200 nuclei were scored for each sample. Statistical significance was determined using one-way ANOVA with Tukey's multiple comparison test. P values are shown only for those samples that differ ($p<0.05$) from the 0-hour time point. (**k**) Immunoblot to assess MCPH1 depletion and expression of WT MCPH1, $MCPH1^{S333A}$ and $MCPH1^{S333D}$ in U2OS cells. The MCPH1 antibody used has been shown to recognize a non-specific band of approximately the same molecular weight of the full length MCPH1 in U2OS cells[1] (top band). The MCPH1 shorter isoform (bottom band) shows a substantial reduction after treatment with MCPH1 shRNA. γ-tubulin was used as loading control. SE: short exposure; LE: long exposure. Representative blot from two independent experiments. (**l**) Quantification of the percentage of U2OS cells showing prophase-like nuclei. The high number of positive cells upon *MCPH1* shRNA treatment confirms functional loss of MCPH1. Data represent the mean of two independent experiments ± SD. At least 300 nuclei were scored for each sample. One-way ANOVA with Tukey's multiple comparison test. (**m**) IF-FISH analysis of MCPH1 (green) telomeric localization in $MCPH1^{+/+}$ and $MCPH1^{\Delta/\Delta}$ cells reconstituted with either empty vector, WT MCPH1, $MCPH1^{S333A}$ and $MCPH1^{S333D}$ and treated with shTRF1. Telomeres were visualized with PNA-FISH (red). Representative images from three

independent experiments. Scale bar: 5 µm. (**n**) Quantification of the percentage of cells with >5 MCPH1-positive foci at telomeres observed in **m**. Data are representative of the mean of three independent experiments, and at least 200 nuclei were scored per experiment. One-way ANOVA followed by Tukey's multiple comparison test.

Supplementary Figure 8

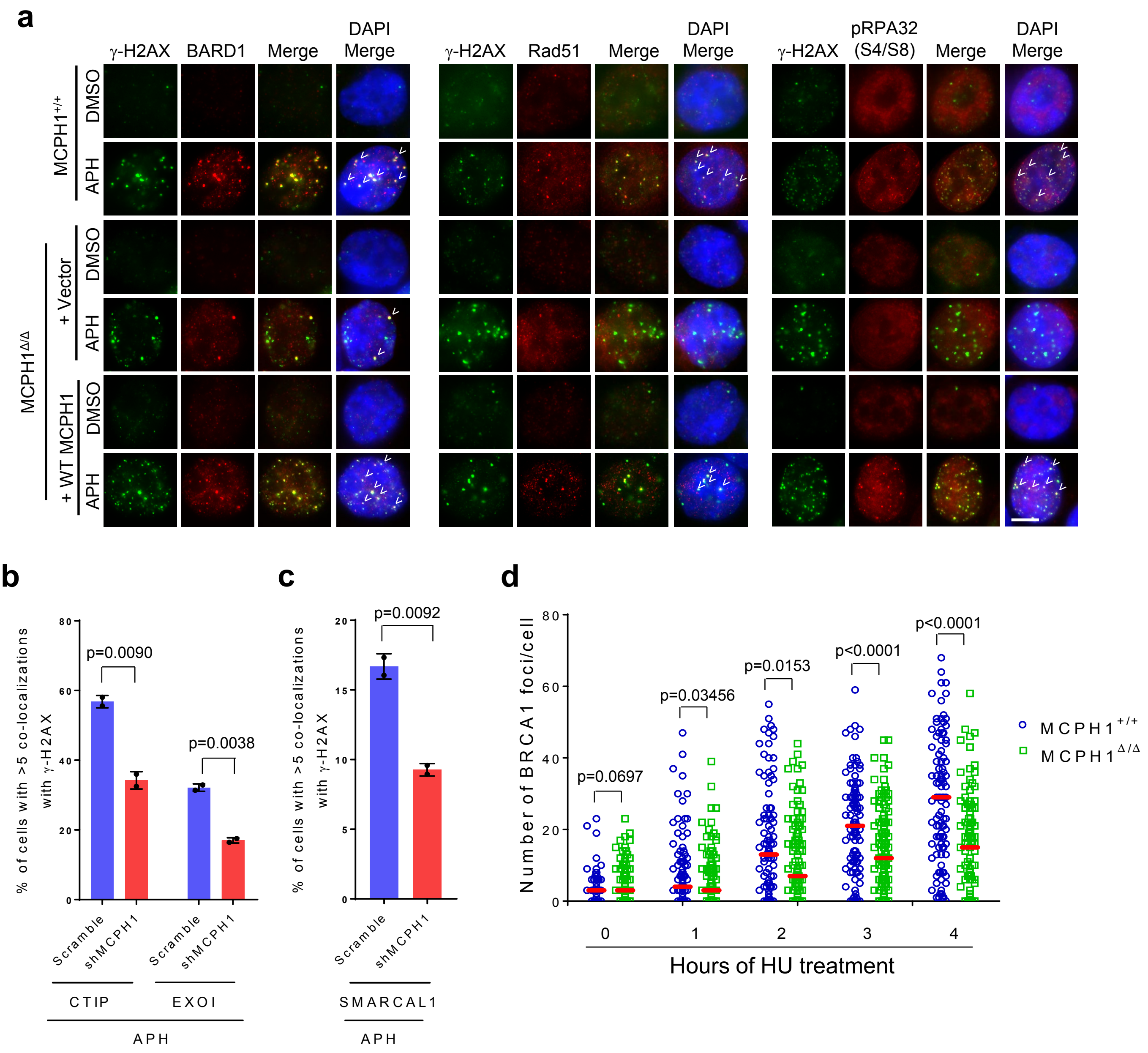

Supplementary Figure 8. MCPH1 promotes genomic DNA replication through recruitment of HDR factors at stalled replication forks. (**a**) Immunostaining for BARD1, RAD51 and p-RPA32 (S4/S8) (green) foci co-localizing with γ-H2AX (red) in MCPH1$^{+/+}$ and MCPH1$^{\Delta/\Delta}$ HCT116 reconstituted with either empty vector or WT MCPH1 and treated for 24 hours with either DMSO or 0.25 μM aphidicolin (APH). Nuclei were visualized with DAPI (blue). Quantifications are shown in Fig. 6e. Representative images from two independent experiments. (**b**) Quantification of the percentage of cells with >5 CTIP and EXOI foci co-localizing with γ-H2AX in U2OS cells transfected with either scrambled or *MCPH1* shRNA and treated for 24 hours with 0.25 μM APH. Data represent the mean from two independent experiments ± SD. At least 200 nuclei were scored per experiment. Two-sided Student's t test. (**c**) Quantification of the percentage of nuclei with >5 SMARCAL1 foci co-localizing with γ-H2AX in U2OS cells transfected with either scrambled or *MCPH1* shRNA and treated for 24 hours with 0.25 μM APH. Data represent the mean from two independent experiments ± SD. At least 200 nuclei were scored per experiment. Two-sided Student's t test. (**d**) Time course experiment to determine the accumulation of BRCA1 foci in MCPH1$^{+/+}$ and MCPH1$^{\Delta/\Delta}$ cells at the indicated time points after the addition of 2 mM hydroxyurea (HU) to the media. 0 hours of HU treatment correspond to untreated cells. Scatter plot represent the data from one of two independent experiments. The red line represents the median value for each sample. P values were determined using two-sided Mann-Whitney test for nonparametric distributions.

Supplementary Table 1

mM HU		MCPH1$^{+/+}$ + Scramble	MCPH1$^{+/+}$ + sh*BRCA1*	MCPH1$^{\Delta/\Delta}$ + Scramble
0	MCPH1$^{+/+}$ + sh*BRCA1*	p>0.9999		
	MCPH1$^{\Delta/\Delta}$ + Scramble	p>0.9999	p>0.9999	
	MCPH1$^{\Delta/\Delta}$ + sh*BRCA1*	p>0.9999	p>0.9999	p>0.9999
0.078	MCPH1$^{+/+}$ + sh*BRCA1*	p=0.6178		
	MCPH1$^{\Delta/\Delta}$ + Scramble	p=0.6563	p=0.9999	
	MCPH1$^{\Delta/\Delta}$ + sh*BRCA1*	p=0.0021	p=0.0093	p=0.0085
0.156	MCPH1$^{+/+}$ + sh*BRCA1*	p=0.0041		
	MCPH1$^{\Delta/\Delta}$ + Scramble	p=0.0018	p=0.8872	
	MCPH1$^{\Delta/\Delta}$ + sh*BRCA1*	p<0.0001	p=0.0086	p=0.0223
0.312	MCPH1$^{+/+}$ + sh*BRCA1*	p=0.3086		
	MCPH1$^{\Delta/\Delta}$ + Scramble	p=0.0014	p=0.0139	
	MCPH1$^{\Delta/\Delta}$ + sh*BRCA1*	p=0.0007	p=0.0056	p=0.8920
0.625	MCPH1$^{+/+}$ + sh*BRCA1*	p=0.3264		
	MCPH1$^{\Delta/\Delta}$ + Scramble	p=0.9182	p=0.1409	
	MCPH1$^{\Delta/\Delta}$ + sh*BRCA1*	p=0.8442	p=0.7465	p=0.5049
1.25	MCPH1$^{+/+}$ + sh*BRCA1*	p=0.0316		
	MCPH1$^{\Delta/\Delta}$ + Scramble	p=0.5907	p=0.1930	
	MCPH1$^{\Delta/\Delta}$ + sh*BRCA1*	p=0.0004	p=0.0254	p=0.0016
2.5	MCPH1$^{+/+}$ + sh*BRCA1*	p=0.0903		
	MCPH1$^{\Delta/\Delta}$ + Scramble	p=0.9229	p=0.2129	
	MCPH1$^{\Delta/\Delta}$ + sh*BRCA1*	p=0.0070	p=0.3087	p=0.0158
5	MCPH1$^{+/+}$ + sh*BRCA1*	p=0.0082		
	MCPH1$^{\Delta/\Delta}$ + Scramble	p=0.0054	p=0.9855	
	MCPH1$^{\Delta/\Delta}$ + sh*BRCA1*	p<0.0001	p=0.0077	p=0.0120

Supplementary Table 1. Significance of the differences in the survival curve showed in Figure 6h for each concentration of hydroxyurea (HU). The statistical significance between the samples in each concentration group was determined using one-way analysis of variance (ANOVA) followed by Tukey's multiple comparison test.

Supplementary Table 2

µM Olaparib		MCPH1$^{+/+}$ + Scramble	MCPH1$^{+/+}$ + sh*BRCA1*	MCPH1$^{\Delta/\Delta}$ + Scramble
0	MCPH1$^{+/+}$ + sh*BRCA1*	p>0.9999		
	MCPH1$^{\Delta/\Delta}$ + Scramble	p>0.9999	p>0.9999	
	MCPH1$^{\Delta/\Delta}$ + sh*BRCA1*	p>0.9999	p>0.9999	p>0.9999
0.312	MCPH1$^{+/+}$ + sh*BRCA1*	p=0.7554		
	MCPH1$^{\Delta/\Delta}$ + Scramble	p=0.7547	p>0.9999	
	MCPH1$^{\Delta/\Delta}$ + sh*BRCA1*	p=0.0036	p=0.0126	p=0.0127
0.625	MCPH1$^{+/+}$ + sh*BRCA1*	p=0.0295		
	MCPH1$^{\Delta/\Delta}$ + Scramble	p=0.1640	p=0.6303	
	MCPH1$^{\Delta/\Delta}$ + sh*BRCA1*	p=0.0002	p=0.0090	p=0.0021
1.25	MCPH1$^{+/+}$ + sh*BRCA1*	p=0.0585		
	MCPH1$^{\Delta/\Delta}$ + Scramble	p=0.4478	p=0.4662	
	MCPH1$^{\Delta/\Delta}$ + sh*BRCA1*	p=0.0051	p=0.3303	p=0.0407
2.5	MCPH1$^{+/+}$ + sh*BRCA1*	p=0.4140		
	MCPH1$^{\Delta/\Delta}$ + Scramble	p=0.1218	p=0.7866	
	MCPH1$^{\Delta/\Delta}$ + sh*BRCA1*	p=0.0011	p=0.0076	p=0.0264
5	MCPH1$^{+/+}$ + sh*BRCA1*	p=0.0736		
	MCPH1$^{\Delta/\Delta}$ + Scramble	p=0.0003	p=0.0078	
	MCPH1$^{\Delta/\Delta}$ + sh*BRCA1*	p=0.0003	p=0.0060	p=0.9964
10	MCPH1$^{+/+}$ + sh*BRCA1*	p=0.0254		
	MCPH1$^{\Delta/\Delta}$ + Scramble	p<0.0001	p=0.0004	
	MCPH1$^{\Delta/\Delta}$ + sh*BRCA1*	p<0.0001	p=0.0002	p=0.9290
20	MCPH1$^{+/+}$ + sh*BRCA1*	p=0.0669		
	MCPH1$^{\Delta/\Delta}$ + Scramble	p=0.0024	p=0.0001	
	MCPH1$^{\Delta/\Delta}$ + sh*BRCA1*	p=0.2194	p=0.0039	p=0.0370
40	MCPH1$^{+/+}$ + sh*BRCA1*	p=0.0185		
	MCPH1$^{\Delta/\Delta}$ + Scramble	p=0.0792	p=0.0006	
	MCPH1$^{\Delta/\Delta}$ + sh*BRCA1*	p=0.3726	p=0.2028	p=0.0076
80	MCPH1$^{+/+}$ + sh*BRCA1*	p=0.0939		
	MCPH1$^{\Delta/\Delta}$ + Scramble	p=0.1214	p=0.0031	
	MCPH1$^{\Delta/\Delta}$ + sh*BRCA1*	p=0.1073	p=0.9997	p=0.0035

Supplementary Table 2. Significance of the differences in the survival curve showed in Figure 6i for each concentration of Olaparib. Statistical significance between the samples at each concentration was determined using one-way ANOVA with Tukey's multiple comparison test.

Supplementary Table 3

Primer name	Primer sequence
MCPH1 sgRNA	5'-CACCGATCCCGCCGTCTGTCATGG-3'
MCPH1 RT-PCR set 1 fw	5'-ATGTAGTGGCCTATGTTGAAGTG-3'
MCPH1 RT-PCR set 1 rv	5'-CCACAAGCTGTGTTGTAAATGTC-3'
MCPH1 RT-PCR set 2 fw	5'-TACAACACAGCTTGTGGATATGG-3'
MCPH1 RT-PCR set 2 rv	5'-CGAGCTTTACGCCTCTCTTCT-3'
MCPH1 RT-PCR set 3 fw	5'-GTAGTCACCCCTGACCAAAAG-3'
MCPH1 RT-PCR set 3 rv	5'-GCAGCCTCGGCATGATAGA-3'
Actin fw	5’-CATGTACGTTGCTATCCAGGC-3’
Actin rv	5’-CTCCTTAATGTCACGCACGAT-3’

Supplementary Table 3. Sequence of the oligonucleotides used.

Supplementary References

1. Meyer SK, Dunn M, Vidler DS, Porter A, Blain PG, Jowsey PA. Phosphorylation of MCPH1 isoforms during mitosis followed by isoform-specific degradation by APC/C-CDH1. FASEB J 33, 2796-2808 (2019).
