# Supplementary material for: Microcephalin 1/BRIT1-TRF2 interaction promotes telomere replication and repair, linking telomere dysfunction to primary microcephaly
Source: Nat Commun. 2020 Nov 17;11:5861. doi: 10.1038/s41467-020-19674-0 (PMC7672075; doi:10.1038/s41467-020-19674-0)
Supplement: Supplementary file 2 — Reporting Summary [file 41467_2020_19674_MOESM2_ESM.pdf]

nature research

Corresponding author(s): Dr. Sandy Chang

Last updated by author(s): Oct 8, 2020

Reporting Summary

Nature Research wishes to improve the reproducibility of the work that we publish. This form provides structure for consistency and transparency in reporting. For further information on Nature Research policies, see our Editorial Policies and the Editorial Policy Checklist.

Statistics

For all statistical analyses, confirm that the following items are present in the figure legend, table legend, main text, or Methods section.

n/a	Confirmed	
☐	☒	The exact sample size (*n*) for each experimental group/condition, given as a discrete number and unit of measurement
☐	☒	A statement on whether measurements were taken from distinct samples or whether the same sample was measured repeatedly
☐	☒	The statistical test(s) used AND whether they are one- or two-sided<br>*Only common tests should be described solely by name; describe more complex techniques in the Methods section.*
☐	☒	A description of all covariates tested
☐	☒	A description of any assumptions or corrections, such as tests of normality and adjustment for multiple comparisons
☐	☒	A full description of the statistical parameters including central tendency (e.g. means) or other basic estimates (e.g. regression coefficient) AND variation (e.g. standard deviation) or associated estimates of uncertainty (e.g. confidence intervals)
☐	☒	For null hypothesis testing, the test statistic (e.g. *F*, *t*, *r*) with confidence intervals, effect sizes, degrees of freedom and *P* value noted<br>*Give P values as exact values whenever suitable.*
☒	☐	For Bayesian analysis, information on the choice of priors and Markov chain Monte Carlo settings
☐	☒	For hierarchical and complex designs, identification of the appropriate level for tests and full reporting of outcomes
☒	☐	Estimates of effect sizes (e.g. Cohen's *d*, Pearson's *r*), indicating how they were calculated

Our web collection on statistics for biologists contains articles on many of the points above.

Software and code

Policy information about availability of computer code

Data collection	Software NIS-Elements BR (Nikon) v3.22.11
Data analysis	ImageJ v1.51k (NIH)<br>GraphPad Prism v7.01<br>FlowJo v10

For manuscripts utilizing custom algorithms or software that are central to the research but not yet described in published literature, software must be made available to editors and reviewers. We strongly encourage code deposition in a community repository (e.g. GitHub). See the Nature Research guidelines for submitting code & software for further information.

Data

Policy information about availability of data

All manuscripts must include a data availability statement. This statement should provide the following information, where applicable:

- Accession codes, unique identifiers, or web links for publicly available datasets
- A list of figures that have associated raw data
- A description of any restrictions on data availability

Coordinate and structure factor have been deposited in the Protein Data Bank under accession code 7C5D (https://www.rcsb.org/structure/7C5D). The authors declare that all other data supporting the findings of this study are available within the paper and its supplementary information files. Source data are provided with this paper.

Field-specific reporting

Please select the one below that is the best fit for your research. If you are not sure, read the appropriate sections before making your selection.

☒ Life sciences ☐ Behavioural & social sciences ☐ Ecological, evolutionary & environmental sciences

For a reference copy of the document with all sections, see nature.com/documents/nr-reporting-summary-flat.pdf

Life sciences study design

All studies must disclose on these points even when the disclosure is negative.

Sample size	No statistical tests were performed to predetermine the sample sizes. Sample sizes were chosen based on previous publications and a sufficient number of replicates was performed to allow calculations of statistical significance.
Data exclusions	No data were excluded from the analysis.
Replication	All the experiments were successfully replicated either two or three times. Details on the number of replicates for each experiment can be found in the associated figure legend. When possible, alternative methods were used to confirm the results.
Randomization	Allocation of samples into the experimental groups was random and the experiments were carried out in parallel.
Blinding	Investigators were blinded to group allocation during both data collection and data analysis for all the experiments involving fluorescence microscopy. Data interpretation of other experiments was based on appropriate controls.

Reporting for specific materials, systems and methods

We require information from authors about some types of materials, experimental systems and methods used in many studies. Here, indicate whether each material, system or method listed is relevant to your study. If you are not sure if a list item applies to your research, read the appropriate section before selecting a response.

Materials & experimental systems

n/a	Involved in the study	
☐	☒	Antibodies
☐	☒	Eukaryotic cell lines
☒	☐	Palaeontology and archaeology
☒	☐	Animals and other organisms
☒	☐	Human research participants
☒	☐	Clinical data
☒	☐	Dual use research of concern

Methods

n/a	Involved in the study	
☒	☐	ChIP-seq
☐	☒	Flow cytometry
☒	☐	MRI-based neuroimaging

Antibodies

Antibodies used

Microcephalin-1/BRIT1 rabbit monoclonal antibody (Cell Signaling Technology), cat #4120, clone D38G5, lot 2. Dilution 1:1000
Phospho-Histone H2AX (S139) mouse monoclonal antibody (Millipore), cat #05-636, clone JBW301, lot 3108494. Dilution 1:1000
53BP1 rabbit polyclonal antibody (Santa Cruz), cat #sc-22760, clone H-300, lot G1114. Dilution 1:1000
BARD1 rabbit polyclonal antibody (Santa Cruz), cat #sc-11438, clone H-300, lot K2211. Dilution 1:1000
BRCA1 mouse monoclonal antibody (Santa Cruz), cat #sc-6954, clone D-9, lot B0414. Dilution 1:1000
RAD51 rabbit polyclonal antibody (Santa Cruz), cat #sc-8349, clone H-92, lot J2014. Dilution 1:500
Phosphorylated RPA32 (S4/S8) rabbit polyclonal antibody (Bethyl), cat #A300-245A, lot 5. Dilution 1:500
Phosphorylated RPA32 (S33) rabbit polyclonal antibody (Bethyl), cat #A300-246A, lot 8. Dilution 1:1000
CTIP rabbit polyclonal antibody (Santa Cruz), cat #sc-22838, clone H-300, lot G0207. Dilution 1:1000
EXOI rabbit polyclonal antibody (Santa Cruz), cat #sc-33194, clone H-300, lot L2208. Dilution 1:500
SMARCAL1 mouse monoclonal antibody (Santa Cruz), cat #sc-376377, clone A-2, lot C1218. Dilution 1:500
TRF2 mouse monoclonal antibody (Millipore), cat #05-521, clone 4A794, lot 3250331. Dilution 1:1000
Cyclin A mouse monoclonal antibody (Santa Cruz), cat #sc-239, clone BF683, lotG2314. Dilution 1:500
FLAG M2 mouse monoclonal antibody (Sigma), cat #F3165, lot SLBT6752. Dilution 1:2000
Myc mouse monoclonal antibody (Millipore), cat #05-724, clone 4A6, lot 3095953. Dilution 1:2000
Gamma-tubulin mouse monoclonal antibody (Sigma), cat# T6557, clone GTU-88, lot 049M4786V. Dilution 1:5000
BrdU mouse monoclonal antibody (BD Biosciences), cat #347580, clone B44. Dilution 1:50
BrdU rat monoclonal antibody (Abcam), cat #Ab6326, clone BU1/75(ICR1), lot GR3289291-1. Dilution 1:50
Biotinylated anti-streptavidin goat antibody (Vector Laboratories), cat #BA-0500, clone ZC0613. Dilution 1:50

Secondary antibodies:
Peroxidase-linked anti-mouse IgG (Amersham), cat #NXA931V, lot 16964893. Dilution 1:5000

Peroxidase-linked anti-rabbit IgG (Amersham), cat #NA934V, lot 16991099. Dilution 1:5000
Alexa Fluor 488 goat anti-mouse (Invitrogen), cat #A11001, lot 2140660. Dilution 1:2000
Alexa Fluor 568 goat anti-mouse (Invitrogen), cat #A11004, lot 927620. Dilution 1:2000
Alexa Fluor 488 goat anti-rabbit (Invitrogen), cat #A11008, lot 2018309. Dilution 1:2000
Alexa Fluor 594 goat anti-rabbit (Invitrogen), cat #A11012, lot 1892265. Dilution 1:2000
Cy3.5-conjugated goat anti-mouse (Abcam), cat #ab6946, lot GR37758-28. Dilution 1:250
Cy5-conjugated goat anti-rat (Abcam), cat #ab6565, lot GR313470-2. Dilution 1:250

Validation

Information on the validation for the antibodies used can be found in the manifacturer's websites.

The Microcephalin-1/BRIT1 antibody (4120) was validated by Meyer SK, Dunn M, Vidler DS, Porter A, Blain PG, Jowsey PA. Phosphorylation of MCPH1 isoforms during mitosis followed by isoform specific degradation by APC/C-CDH1. FASEB J 33, 2796-2808 (2019). Manifacturer's website: https://www.cellsignal.com/products/primary-antibodies/microcephalin-1-brit1-d38g5-rabbit-mab/4120?Ntk=Products&Ntt=4120.

Phospho-Histone H2AX (S139) (05-636): validation can be found in the manifacturer's website https://www.emdmillipore.com/US/en/product/Anti-phospho-Histone-H2A.X-Ser139-Antibody-clone-JBW301,MM_NF-05-636. Used in several studies for immunostaining experiments: d'Adda di Fagagna F., et al. (2003) Nature: 426:194-8. Lin J.R., et al. (2015) Nat. Commun. 6:8390. Rai R., et al. (2019) Cell Rep. 29(11):3708-3725.e5.

53BP1 antibody (sc-22760): https://www.scbt.com/p/53bp1-antibody-h-300. Validated for wester blot and immunostaining studies in human and mouse cells: Bekker-Jensen S., et al. (2006) J. Cell Biol. 173: 195-206. Li B., et al. (2008) Mol. Cell. Biol. 28: 1892-1904. Rai R., et al. (2019) Cell Rep. 29(11):3708-3725.e5.

BARD1 antibody (sc-11438): https://www.scbt.com/p/bard1-antibody-h-300. Validated for western blot and immunostaining studies in human and mouse cells. Wei L., et al. (2008) Mol. Cell. Biol. 28: 7380-7393. Zimmermann M., et al. (2013) Science 339: 700-4. Rai R., et al. (2019) Cell Rep. 29(11):3708-3725.e5. Rai R., et al. (2016) Nature Commun. 7: 10881.

BRCA1 antibody (sc-6954): https://www.scbt.com/p/brca1-antibody-d-9. Validated for western blot analysis and immunostaining in human cell lines. Kim J., et al. (2019) Nat. Struct. Mol. Biol. 26: 213-219. Rai R., et al. (2019) Cell Rep. 29(11):3708-3725.e5. Jayavaradhan R., (2019) Nat Commun. 10: 2866. Gupta R., et al. (2018) Cell. 173: 972-988.e23.

RAD51 antibody (sc-8349): https://www.scbt.com/p/rad51-antibody-h-92. Validated for immunostaining in human and mouse cell lines. Nicolae C.M., et al. (2015) Nucleic acids research. 43: 3143-53. Rai R., et al. (2016) Nature communications. 7: 10881. Pathania S., et al. (2014) Nature Commun. 5: 5496.

Phosphorylated RPA32 (S4/S8) antibody (A300-245A): https://www.bethyl.com/product/A300-245A/Phospho+RPA32+(S4+S8)+Antibody. Validated for Western blot and immunostaining of both tissues and cultured human cells. Pan X., et al. (2019) Scientific Reports 9:19110. Rai R., et al. (2019) Cell Rep. 29(11):3708-3725.e5. Kim W., et al. (2019) Nat. Commun. 10:5304.

Phosphorylated RPA32 (S33) antibody (A300-246A): https://www.bethyl.com/product/A300-246A/Phospho+RPA32+(S33)+Antibody. Validated for Western blot in human cells. Several research articles show immunostaining data obtained with this antibody. Silva B., et al. (2019) Nat. Commun. 10: 2253. Wang Y.H., et al. (2017) Nat. Commun. 8: 2118. Zhang B., et al. (2014) J Biol. Chem. 289(49): 34284-34295.

CTIP antibody (sc-22838): https://www.scbt.com/p/ctip-antibody-h-300. Validated for Western blot and immunostaining in both human tissues and human cell lines. Rai R., et al. (2019) Cell Rep. 29(11):3708-3725.e5. Wu M., et al. (2007) Mol. Cancer Res. 5: 1285-1295.

EXOI antibody (sc-33194): https://datasheets.scbt.com/sc-33194.pdf. Validated for Western blot and immunostaining in human cell lines. Rai R., et al. (2019) Cell Rep. 29(11):3708-3725.e5.

SMARCAL1 antibody (#sc-376377): https://www.scbt.com/p/smarcal1-antibody-a-2. Validated for Western blot and immunostaining of human cell lines and human tissues. Rai R., et al. (2019) Cell Rep. 29(11):3708-3725.e5. Nazeer R., et al. (2019) J Virol. 93(13): e00402-19.

TRF2 antibody (05-521): https://www.emdmillipore.com/US/en/product/Anti-TRF2-Antibody-clone-4A794,MM_NF-05-521. Validated by immunoblot on RIPA lysate of human Jurkat cells and HeLa nuclear extract. Rai R., et al. (2019) Cell Rep. 29(11):3708-3725.e5. Wakai M., et al. (2014) PloS One 9: e88530. Delbarre E., et al. (2013) Genome Res. 23(3):440-51.

Cyclin A antibody (#sc-239): https://www.scbt.com/p/cyclin-a-antibody-bf683. Validated for western blot in human cell line lysates. Rai R., et al. (2017) Mol Cell 65(5): 801–817.e4. Pattschull G., et al. (2019) Cell Rep. 27: 3533-3546.e7.

FLAG M2 antibody (F3165): https://www.sigmaaldrich.com/catalog/product/sigma/f3165?lang=en&region=US. Validated for Western blot and immunostaining for the detection of FLAG tag. Rai R., et al. (2019) Cell Rep. 29(11):3708-3725.e5. Sriramachandran A. M., et al. (2019) Nat Commun. 15;10(1):3678. Doyle S. L., et al. (2012) Nat Commun. 28;3:707.

Myc antibody (05-724): https://www.emdmillipore.com/US/en/product/Anti-Myc-Tag-Antibody-clone-4A6,MM_NF-05-724. Validated for use in ChIP, IC, IF, IP, WB for the detection of Myc Tag. Rai R., et al. (2019) Cell Rep. 29(11):3708-3725.e5. Zhang S., et al. (2015) Nat Neurosci. 18(3):386-92.

Gamma-tubulin antibody (T6557): https://www.sigmaaldrich.com/catalog/product/sigma/t6557?lang=en&region=US. Validated for Western blot and immunostaining in human cell lines. Rai R., et al. (2019) Cell Rep. 29(11):3708-3725.e5. Rai R., et al. (2017) Mol Cell 65(5): 801–817.e4. Rai R., et al. (2016) Nature Commun. 7: 10881.

BrdU mouse monoclonal antibody (347580): https://www.bdbiosciences.com/us/applications/research/apoptosis/purified-

antibodies/purified-mouse-anti-brdu-b44/p/347580. Validated for both flow cytometry and immunofluorescence in human cells. This antibody was used previously for Single Molecule Analysis of Replicated DNA: Pan X., et al. (2017) Proc Natl Acad Sci USA 18;114(29):E5940-E5949. Nieminuszczy J., et al. (2016) Methods 108:92-8. Gali H., et al. (2019) Methods Mol Biol. 1999:319-325.

BrdU rat monoclonal antibody (Ab6326): https://www.abcam.com/brdu-antibody-bu175-icr1-proliferation-marker-ab6326.html. Validated for immunofluorescence in human cell lines and tissues and for flow cytometry. This antibody was used previously for Single Molecule Analysis of Replicated DNA: Nieminuszczy J., et al. (2016) Methods 108:92-8. Gali H., et al. (2019) Methods Mol Biol. 1999:319-325.

Biotinylated anti-streptavidin antibody (BA-0500): https://vectorlabs.com/biotinylated-anti-streptavidin-antibody.html#biozbadges. Validated for immunofluorescence and in-situ hybridization. This antibody has been used before for Single Molecule Analysis of Replicated DNA. Pan X., et al. (2017) Proc Natl Acad Sci USA 18;114(29):E5940-E5949. Gali H., et al. (2019) Methods Mol Biol. 1999:319-325.

Eukaryotic cell lines

Policy information about cell lines

Cell line source(s)	HCT116: ATCC (CCL-247); 293T: ATCC (CRL-3216); U-2 OS: ATCC (HTB-96); WT MEFs and H2AX -/- MEFs: generated in Sandy Chang lab; IMR-90: ATCC (CCL-186); HeLa: ATCC (CCL-2).
Authentication	These cell lines are routinely used in our lab and we constantly monitor their morphology.
Mycoplasma contamination	All cell lines tested negative for mycoplasma contamination by PCR.
Commonly misidentified lines (See ICLAC register)	No commonly misidentified cell lines were used in this study.

Flow Cytometry

Plots

Confirm that:

☒ The axis labels state the marker and fluorochrome used (e.g. CD4-FITC).

☒ The axis scales are clearly visible. Include numbers along axes only for bottom left plot of group (a 'group' is an analysis of identical markers).

☒ All plots are contour plots with outliers or pseudocolor plots.

☒ A numerical value for number of cells or percentage (with statistics) is provided.

Methodology

Sample preparation	U-2 OS cells were fixed in 70% ice-cold ethanol for at least 24 hours at -20°C, washed twice with PBS and then resuspended in 1 ml PBS containing 50 µg/ml of Propidium Iodide and 100 µg/ml of RNAse A. After incubation at 4°C overnight, the samples were analyzed by flow cytometry.
Instrument	BD LSR Fortessa cytometer
Software	FlowJo (FlowJo LLC)
Cell population abundance	2000000 cells for each sample.
Gating strategy	All samples were gated through SSC-A/FSC-A gating. A figure demonstrating the gating strategy has been provided in the Supplementary Figures.

☒ Tick this box to confirm that a figure exemplifying the gating strategy is provided in the Supplementary Information.
