# Supplementary material for: Microcephalin 1/BRIT1-TRF2 interaction promotes telomere replication and repair, linking telomere dysfunction to primary microcephaly
Source: Nat Commun. 2020 Nov 17;11:5861. doi: 10.1038/s41467-020-19674-0 (PMC7672075; doi:10.1038/s41467-020-19674-0)
Supplement: Supplementary file 3 — Source Data [file 41467_2020_19674_MOESM3_ESM.zip › Extended comments checklist.docx]

Your manuscript has been checked for clarity and against journal policies and formatting style. The issues listed below must be addressed; failure to do so will cause delays in acceptance.

For further information, please see our [formatting instructions](https://www.nature.com/documents/ncomms-formatting-instructions.pdf).

Please highlight all changes in the manuscript text file, either using the track changes feature in Microsoft Word or coloured highlighting in LaTeX.

Please include your response to these requests in the space provided and return this checklist with your final submission.

| **EDITORIAL REQUESTS:** | **AUTHOR RESPONSE:** |
| --- | --- |
| The main manuscript file must be provided in Microsoft Word or LaTeX format. | The manuscript has been provided in Microsoft Word format. |
| **POLICIES AND CHECKLISTS** | **POLICIES AND CHECKLISTS** |
| An updated editorial policy checklist must be completed and uploaded as a related manuscript file with the revised manuscript. All points on the policy checklist must be addressed; if needed, please revise your manuscript in response to these points. Please note that this form is a dynamic 'smart pdf' and must therefore be downloaded and completed in Adobe Reader, instead of opening it in a web browser. https://www.nature.com/authors/policies/Policy.pdf | We have included the updated editorial policy checklist. |
| Please also find below a list of comments requesting additional information in the figure legends, text, and methods section to comply with our reporting policies. |  |
| **TITLE PAGE (page 2 of our formatting instructions)** | **TITLE PAGE (page 2 of our formatting instructions)** |
| Please ensure that all affiliations are in the correct sequential order according to their position in the author list. Affiliation 1 must be the first affiliation associated with the first author. Please see this article as an example: https://www.nature.com/articles/s41467-020-16621-x.pdf | The order of the affiliation has been corrected. |
| **LANGUAGE AND STYLE (page 6 of our formatting instructions)** | **LANGUAGE AND STYLE (page 6 of our formatting instructions)** |
| Please remove phrases such as 'new', 'novel', 'for the first time', 'unprecedented', etc., as novelty is clear from the context. Please also remove exaggerated language such as 'extremely', 'outstanding', etc. | We removed those phrases as requested. |
| Please do not use italics, bold font, underlining or speech marks unless required for technical terms (in both the main text and the display items). | We used italics when referencing gene names, since this is the conventional way to write them. |
| **METHODS AND DATA (page 3 of our formatting instructions)** | **METHODS AND DATA (page 3 of our formatting instructions)** |
| Sufficient details of the experiments must be provided in the Methods section such that they could be reproduced without reference to published papers. Use of the term 'as described previously' is not encouraged. | Details have been added for all experimental procedure that were not described. |
| A complete list of all primers used, including the names and sequences, must be supplied as a Supplementary Table, which must be cited once in the Methods section. | A list of the primers used has been included as Supplementary Table 3. |
| Centrifugation speeds should be described in xg, not rpm. | Centrifugation speed is indicated in xg. |
| Your manuscript does not include a Data Availability section, which is a requirement for publication in the journal. Please see the guidance below and ensure your revised manuscript contains this section. | We included the Data Availability section. |
| All manuscripts must include a Data Availability statement as a separate section after the Methods section but before the References. For acceptable examples, see: https://www.nature.com/documents/nr-data-availability-statements-data-citations.pdf The Data Availability statement should include:  - Accession codes with hyperlinks for deposited data   - Other unique identifiers (such as DOIs and hyperlinks for any other datasets)   - At a minimum, a statement confirming that all relevant data are available from the authors   - If applicable, a statement regarding data available with restrictions  - If a dataset has a Digital Object Identifier (DOI) as its unique identifier, we strongly encourage including this in the Reference list and citing the dataset in the Data Availability Statement |  |
| In particular, please release all raw data including PDB and sequencing data which has not yet been publically released. | We wrote to the PDB staff to release the crystal structure in the next available release date. |
| Nature Research policies (https://go.nature.com/data-availability-AIP) strongly encourage deposition of research data in public repositories. In some cases this is mandatory, and you may have been previously advised if that was the case. If you need help depositing and curating your research data (including raw and processed data, text, video, audio and images) you should consider: - Contacting Springer Nature’s Research Data Helpdesk (https://go.nature.com/helpdesk-AIP) for advice - Finding a suitable data repository (https://go.nature.com/RD-policies-AIP) for your data - Uploading your data to Springer Nature’s Research Data Support service (https://go.nature.com/RDS-AIP) Research Data Support is an optional Springer Nature service. There are fees (https://go.nature.com/RDS-pricing-AIP) for using this service, however, if you receive funding from the Wellcome Trust or are affiliated to a Wellcome Centre you can use Research Data Support at no cost. See https://go.nature.com/wellcome-RDS-AIP for more information. If you choose to use Research Data Support, please do not submit your revised manuscript until you have been supplied with the DOI for your data by the Research Data Support team. Please also ensure that you update your Data Availability statement with this DOI and the information provided by the team. Please provide a unique identifier for the data (for example a DOI or a permanent URL) in the data availability statement, if possible. If the repository does not provide identifiers, we encourage authors to supply the search terms that will return the data. For data that have been obtained from publicly available sources, please provide a URL and the specific data product name in the data availability statement. Data with a DOI should be included in the reference list and cited where relevant. Alternatively, include the data in the Supplementary Information. For datasets for which mandatory deposition is not required and the data can only be shared on request, please explain why in your Data Availability Statement and in your response here.  Please refer to our data policies here: http://www.nature.com/authors/policies/availability.html | The TRF2-TRFH/MCPH1-TBM crystal structure has been deposited in the PDB website and the corresponding accession code and hyperlink have been included in the Data Availability section. All the other data supporting the study are included in the manuscript, in the Supplementary Information or in the Source Data file. |
| All accession codes must be accompanied with their hyperlinks throughout (for example, "5XRN [http://doi.org/10.2210/pdb5XRN/pdb]", "1483958 [https://doi.org/10.5517/ccdc.csd.cc1lt5m6]", "SRP109982 [https://www.ncbi.nlm.nih.gov/sra/?term=SRP109982]", "GSE101099 [https://www.ncbi.nlm.nih.gov/geo/query/acc.cgi?acc=GSE101099]" or "NQLW00000000 [https://www.ncbi.nlm.nih.gov/assembly/GCA_002312845.1/]"). | The hyperlink to the crystal structure in PDB has been included in the Data Availability section. |
| A reference to the source data file should be added in the 'Data Availability' section, using the text “Source data are provided with this paper.” | We included this reference. |
| **DISPLAY ITEMS (pages 4 and 5 of our formatting instructions)** | **DISPLAY ITEMS (pages 4 and 5 of our formatting instructions)** |
| The use or adaptation of previously published images is strongly discouraged. If this is unavoidable, please request the necessary rights documentation to re-use such material from the relevant copyright holders and return this to us when you submit your revised manuscript. Please check whether your manuscript or Supplementary Information contain third-party images, such as figures from the literature, stock photos, clip art or commercial satellite and map data. | We did not use previously published images. |
| Figure legends should not exceed 350 words. Please shorten by removing detailed methodological information and/or interpretation, or, if appropriate, consider splitting the affected figures in two. Note that we allow up to 10 display items (figures and tables) in the main manuscript. | We shortened the figure legends to remain in the 350 words limit. |
| Any abbreviations, symbols or colours present in your figures must be defined in the associated legends. | All the abbreviation, symbols and colours are defined in the figure legends. |
| Please remove the tables from the figures and supply as separate tables. All tables may need to be renumbered so that they appear in numerical order in the main text. | The table presented in panel g of the former Figure 1 has been removed from the figure and is now indicated as Table 2. |
| Tables must be black and white, and data must be free from bold/italic formatting unless this has been clearly defined in the footnote. Tables must not include vertical/horizontal/diagonal dividing lines to separate text within the same cell. Text should be split into separate cells and aligned using the appropriate columns and rows (e.g. to put data on a second line). A table must have the same length and width throughout. If not, please ensure that they are made into separate tables and numbered separately. Please format your tables accordingly. | Tables have been formatted as requested. |
| Tables must be editable and prepared using the table menu in Word or the table environment in LaTeX. | Tables have been prepared in Word using the table menu. |
| All tables must be cited in the order in which they appear in the text, figures, tables and boxes. If a table needs to be referred to out of order, please use the text 'see below' instead of a citation. | Tables are cited and numbered in the order they appear in the text. |
| **SUPPLEMENTARY INFORMATION (page 5 of our formatting instructions)** | **SUPPLEMENTARY INFORMATION (page 5 of our formatting instructions)** |
| We do not edit Supplementary Information files; they will be uploaded with the published article as they are submitted with the final version of your manuscript. Any tracked changes should be removed from the file and the file should be provided as a PDF file. Supplementary Figures do not need to be provided separately. | The Supplementary Information file is provided in pdf, it does not contain tracked changes and it includes the Supplementary Figures. |
| Please ensure that a Source Data file is included with your resubmission. Within the Source Data file, the relevant raw data from each figure or table (in the main manuscript and in the Supplementary Information) should be represented by a single sheet in an Excel document, or a single .txt file or other file type in a zipped folder. Uncropped blots and gel images should be pasted in and labelled with the relevant panel and identifying information such as the antibody used. An example of the Source Data file is available demonstrating the correct format: https://www.nature.com/documents/ncomms-example-source-data.xlsx The file should be labelled 'Source Data', with the title and a brief description included in your response here, and should be mentioned in all relevant figure legends using the template text below: "Source data are provided as a Source Data file." | We included a Source Data excel file containing raw data and uncropped blots.  Since the space in the figure legends was limiting, we mentioned the Source Data file in the methods section, when describing the experiments for which raw data are provided. |
| **PUBLICATION** | **PUBLICATION** |
| Your paper will be accompanied by a two-sentence Editor's summary, of between 250-300 characters including spaces, when it is published online. I have drafted the summary below. If you would like to make changes to this, please provide me with a suitably edited version. | We approve the summary that was provided. |
| Primary microcephaly is a clinical feature of several human telomere disorder syndromes. Here the authors reveal a role of Microcephalin 1 in promoting telomere replication and repair. |  |
| As part of our efforts to communicate our content to a wider audience, we endeavour to highlight papers published in Nature Communications on the journal’s Twitter account (https://twitter.com/NatureComms). If you would like us to mention authors, institutions or lab groups in these tweets, please provide the relevant twitter handles. |  |

|  | **EDITORIAL REQUESTS:** | **AUTHOR RESPONSE:** |
| --- | --- | --- |
| **1.** | **Data presentation:** Please ensure that data presented in a plot, chart or other visual representation format shows data distribution clearly (e.g. dot plots, box-and-whisker plots). When using bar charts, please overlay the corresponding data points (as dot plots) whenever possible and always for n ≤ 10. (Please see the following editorial for the rationale behind this request and an example <https://www.nature.com/articles/s41551-017-0079>). | |
|  | **Panels requiring revision:**  Please note that data presentation has to be revised to comply with our policy in figure 4c; supplementary figures 4i; 6d. | We revised the requested figures. Dot plots corresponding to data points have been overlaid in all the bar charts we presented. |
| **2.** | **Statistics**: Wherever statistics have been derived (e.g. error bars, box plots, statistical significance) the legend needs to provide and define the n number (i.e. the sample size used to derive statistics) as a precise value (not a range), using the wording “n=X biologically independent samples/animals/cells/independent experiments/n= X cells examined over Y independent experiments” etc. as applicable. | |
|  | **Legends requiring revision:**   1. Please note that this information is missing in the legend of figure 5f. | We have included this information in the figure since the space in the figure legend was limited. |
| **3.** | Statistics such as error bars, significance and p values cannot be derived from n<3 and must be removed from all such cases.  Please note that statistics such as error bars must be removed if n<3, as applicable from the figures 3d-g, I; 4b-c, e; 5b, d; 6b, d-e; supplementary figures 2d, f, h, j; 3c, f; 4c, m-o; 5a, c, e, g, I, k, m; 6b, d; 7b, g, j, m; 8b-c. Please note that this should be rectified for these figures. | |
|  | We strongly discourage deriving statistics from technical replicates, unless there is a clear scientific justification for why providing this information is important. Conflating technical and biological variability, e.g., by pooling technically replicates samples across independent experiments is strongly discouraged. (For examples of expected description of statistics in figure legends, please see the following <https://www.nature.com/articles/s41467-019-11636-5> or <https://www.nature.com/articles/s41467-019-11510-4>). | |
|  | All error bars need to be defined in the legends (e.g. SD, SEM) together with a measure of centre (e.g. mean, median). For example, the legends should state something along the lines of “Data are presented as mean values +/- SEM” as appropriate.  All box plots need to be defined in the legends in terms of minima, maxima, centre, bounds of box and whiskers and percentile. | |
| **4.** | The figure legends must indicate the statistical test used. Where appropriate, please indicate in the figure legends whether the statistical tests were one-sided or two-sided and whether adjustments were made for multiple comparisons.  For null hypothesis testing, please indicate the test statistic (e.g. F, t, r) with confidence intervals, effect sizes, degrees of freedom and P values noted.  Please provide the test results (e.g. P values) as exact values whenever possible and with confidence intervals noted. | |
|  | **Legends requiring revision:**   1. Please note that the information on whether the statistical test used was one-sided or two-sided, where appropriate, is missing in the legends of figures 5d, f, h; 6d, g; supplementary figures 2d, f, h; 3c; 4c; 5a, c; 7f, i; 8b-d. 2. Please note that the exact p value should be provided, when possible, in the legends of figures 2g, d; 3b, d-g, i; 4e; 5b, d, h; 6b, d, e, g; supplementary figures 2d, f, h, j; 3c, f; 4c, i, j-k, m-o; 5a, c, e, g, i, k, m; 6b; 7b, f, g, i-j, l, n; 8b-c; supplementary tables 1; 2. | We included the missing information on the statistical tests.  The exact p values (to the fourth decimal digit) were provided in the figures due to limited space in the figure legends. |
| **5.** | **Reproducibility:** Please state in the legends how many times each experiment was repeated independently with similar results. This is needed for all experiments, but is particularly important wherever results from representative experiments (such as micrographs) are shown. If space in the legends is limiting, this information can be included in a section titled “Statistics and Reproducibility” in the methods section. | |
|  | **Legends requiring revision:**  Please note that this information is missing in the legends of figures 2b-c, f; 3a, c, h; 4a, d; 5a, c, e, g; 6a, c, f; supplementary figures 2-c, e, g, i; 3a, b, e, d; 4a, b, f, g, h, l, e; ; 5a, c, e, g, i, k, m; 6a-c; 7b, g, i-j, l, n; 8b-c. | We have included this information in all the figure legends requested. |
| **6.** | **Data availability:** This journal strongly supports public availability of data and custom code associated with the paper in a persistent repository where they can be freely and enduringly accessed or as a supplementary data file when no appropriate repository is available. If data and code can only be shared on request, please explain why in your data Availability Statement, and also in the correspondence with your editor. For more information, please refer to <https://www.nature.com/nature-research/editorial-policies/reporting-standards#availability-of-data> | |
|  | Please ensure that datasets deposited in public repositories are now publicly accessible, and that accession codes or DOI are provided in the "Data Availability" section. As long as these datasets are not public, we cannot proceed with the acceptance of your paper. For data that have been obtained from publicly available sources, please provide a URL and the specific data product name in the data availability statement. Data with a DOI should be further cited in the methods reference section. | We requested PDB to release the crystal structure at the earliest release date. Accession code and hyperlink are indicated in the Data Availability section. |
| **7.** | **Gels and Blots:** Quantitative comparisons between samples on different gels/blots are discouraged; if this is unavoidable, the figure legend must state that the samples derive from the same experiment and that gels/blots were processed in parallel.  Vertically sliced images that juxtapose lanes that were non-adjacent in the gel must have a clear separation or a black line delineating the boundary between the gels. Loading controls (e.g. GAPDH, actin) must be run on the same blot.  Sample processing controls run on different gels must be identified as such in the figure legends, and distinctly from loading controls.  All blots and gels must be accompanied by the locations of molecular weight/size markers. Blots should be cropped such that at least one marker position is present.  Please also supply uncropped and unprocessed scans of the most important blots in the Source Data file or as a supplementary figure in the Supplementary Information. This should be cited once in the Methods section. For an example of presentation of full scan blots, see the Source Data file of <https://www.nature.com/articles/s41467-020-16984-1#Sec35> and for more information, please refer to <https://www.nature.com/nature-research/editorial-policies/image-integrity> | |
|  | **Panels requiring revision:**   1. Molecular weight markers are missing for panels 2b; 5c; supplementary figures 2a, b; 3a, d; 4a, e; 6a, c. 2. Full scans are missing for panels 2b; 5c; supplementary figures 2a, b; 3a, d; 4a, e; 6a, c. | Molecular weight markers have been included in all the presented western blot. The uncropped scans are provided in the Source Data file. |
| **8.** | Micrographs: Please ensure that all micrographs include a scale bar and this scale bar is defined on the panels or in the figure legends. | |
|  | **Panels requiring revision:**   1. Please note that scale bar is missing for figures 2c, f; 3a, c, h; 4a, d; 5a, e, g; 6a, c, f; supplementary figures 2c, e, g, i; 3b, e; 4b, f, g, h, l; 5b, d, f, h, j, l; 6d, f, g, h; 7a, d, e, h, m; 8a. | We added the scale bars and defined them in the figure legends. |
| **9.** | **Flow cytometry data:** Please provide a Supplementary Figure to graphically account for all FACS sequential gating/sorting strategies, or provide gating/sorting strategies in-figure. If the former, please be sure to indicate, in the Supplementary Figure legend, which gating panel(s) correspond to which FACS data panel(s) in the manuscript figures. (For an example, please see <https://www.nature.com/articles/ncomms15067#supplementary-information>). | |
